# Investigating and Quantifying Molecular Complexity Using Assembly Theory and Spectroscopy

**DOI:** 10.1021/acscentsci.4c00120

**Published:** 2024-04-18

**Authors:** Michael Jirasek, Abhishek Sharma, Jessica R. Bame, S. Hessam M. Mehr, Nicola Bell, Stuart M. Marshall, Cole Mathis, Alasdair MacLeod, Geoffrey J. T. Cooper, Marcel Swart, Rosa Mollfulleda, Leroy Cronin

**Affiliations:** † School of Chemistry, The University of Glasgow, University Avenue, Glasgow G12 8QQ, U.K.; ‡ University of Girona, Campus Montilivi (Ciencies), c/M.A. Capmany 69, 17003 Girona, Spain; § ICREA, Pg. Lluis Companys 23, 08010 Barcelona, Spain

## Abstract

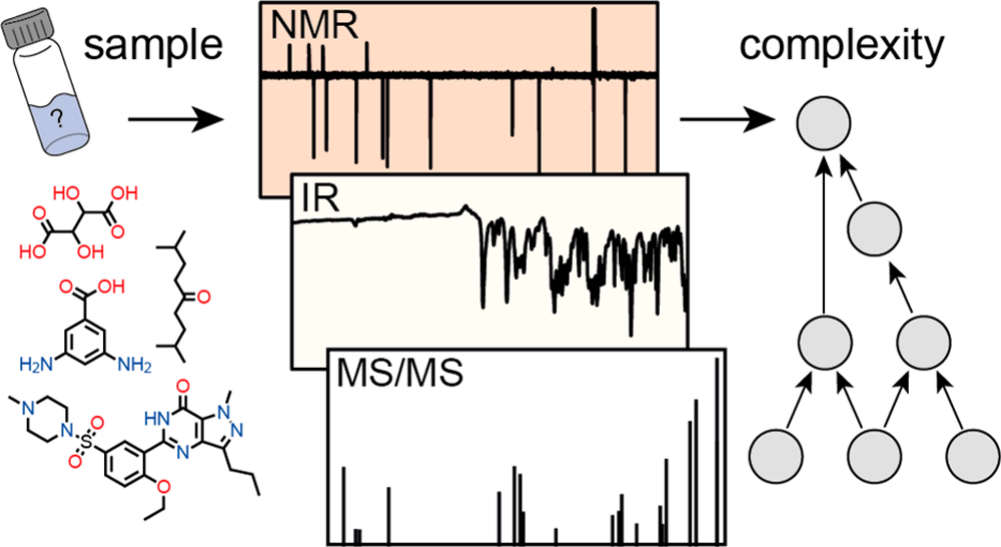

Current approaches to evaluate molecular complexity use algorithmic complexity, rooted in computer science, and thus are not experimentally measurable. Directly evaluating molecular complexity could be used to study directed vs undirected processes in the creation of molecules, with potential applications in drug discovery, the origin of life, and artificial life. Assembly theory has been developed to quantify the complexity of a molecule by finding the shortest path to construct the molecule from building blocks, revealing its molecular assembly index (MA). In this study, we present an approach to rapidly infer the MA of molecules from spectroscopic measurements. We demonstrate that the MA can be experimentally measured by using three independent techniques: nuclear magnetic resonance (NMR), tandem mass spectrometry (MS/MS), and infrared spectroscopy (IR). By identifying and analyzing the number of absorbances in IR spectra, carbon resonances in NMR, or molecular fragments in tandem MS, the MA of an unknown molecule can be reliably estimated. This represents the first experimentally quantifiable approach to determining molecular assembly. This paves the way to use experimental techniques to explore the evolution of complex molecules as well as a unique marker of where an evolutionary process has been operating.

## Abstract 2

This study introduces a novel approach to experimentally measure molecular complexity (MA) using nuclear magnetic resonance, tandem mass spectrometry, and infrared spectroscopy.

## Introduction

The exploration of chemical space reveals the striking fact that most molecules greater than the molecular weight of 300 Da, which are not simple oligomers or composed of heavy atoms, are all connected to the existence of life on Earth.^1^ It has been shown that complex molecules such as natural products^2^ are too complex to form by chance in any detectable abundance and, therefore, can only be made by the complex biochemical pathways found in biological cells. Currently, the exploration of complex chemical space is done *in silico*,^3,4^ and this focuses on chemical structure,^5^ topological features,^6^ application-specific physicochemical descriptors, and graph theory and tends to explore medicinal chemical space for drug discovery and development.^7^ In this regard pharmaceutical products can also be considered to be biosignatures, or more specifically technosignatures, since many are complex and would not have been made without humans using technology.^8−10^ In addition to targeted selectivity, synthetic accessibility is important to explore the complexity of the molecule.^11^ There are many competing notions of molecular complexity,^12^ which have led to different algorithmic methodologies being developed using metrics based on molecular weight, counting chiral centers or primarily focusing on substructure properties, etc.^13−15^ However, with the recent development in algorithmic chemical exploration,^16^ a proxy for complexity is required that is fast to estimate molecular complexity directly from the acquired experimental data, instead of performing complete structure elucidation. Additionally, for biosignature detection,^17^ it is important that the complexity metric can be estimated directly from the experimental data without any assumptions about the local environment or chemistry due to the minimalistic information available for an unknown sample.

**Figure 1:**
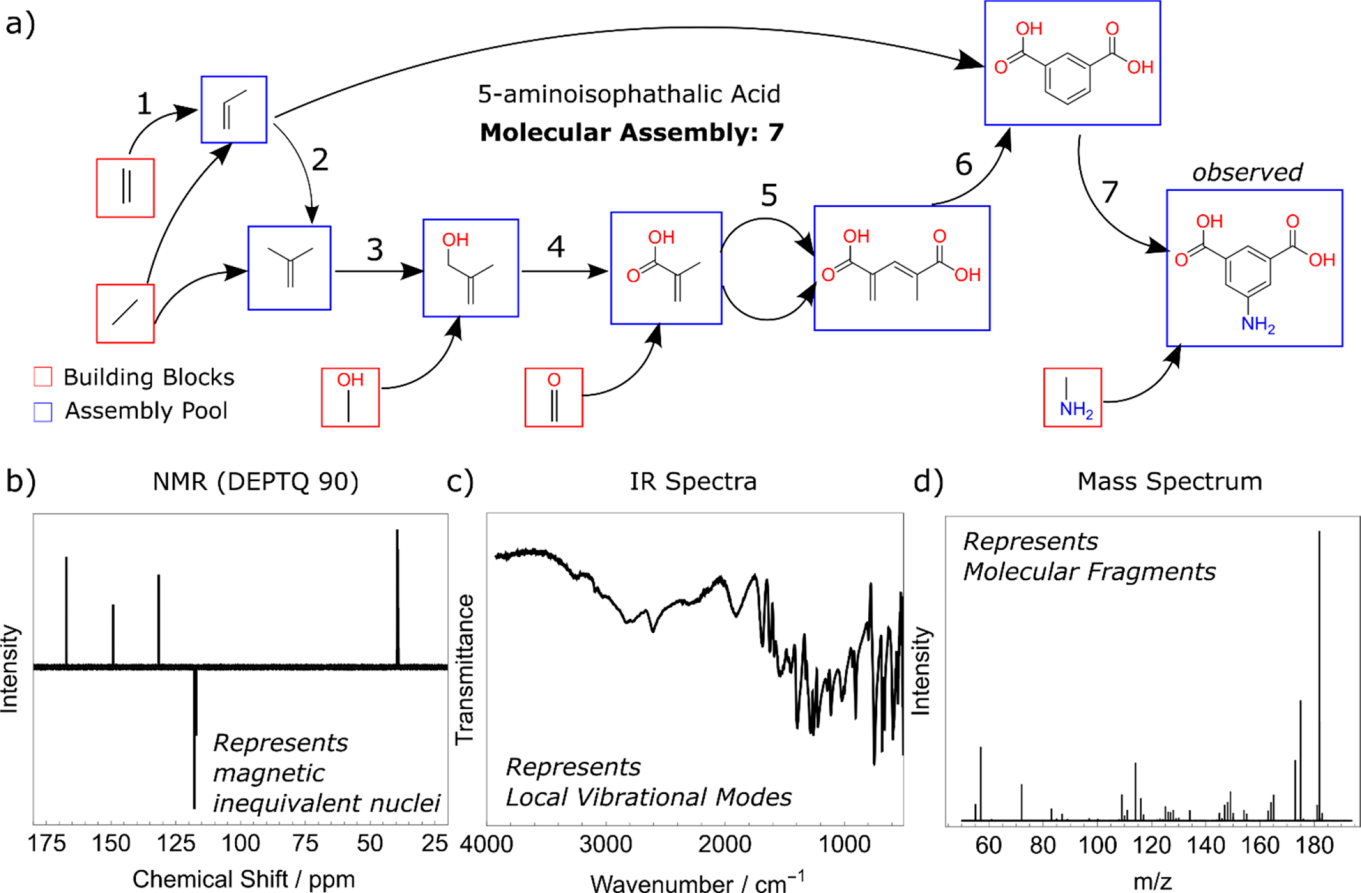
Molecular assembly of 5-aminoisophathalic acid. (A) Molecular assembly pathway of 5-aminoisophathalic with a total of 7 steps. The various chemical bonds are considered as fundamental building blocks (shown in red), and the substructures (shown in blue) along the pathways constitute the assembly pool. (B–D) Experimental NMR, IR, and MS^2^ spectra of 5-aminoisophathalic acid highlighting different features of the molecule from which the molecular constraints and the MA can be inferred (see Figures 3, 4, and 6).

Recently, we developed a novel approach to quantify and explore the complexity of molecules using assembly theory (AT).^18^ Assembly theory estimates the complexity of a molecule by quantifying the minimum constraints required to construct an object from the building blocks. The assembly pathway gives the shortest path to create an object in the absence of physical constraints and reuse of the substructures formed along the pathway. The complexity of an object is therefore defined by the number of steps along the assembly pathway and is called the assembly index,^19^ which for molecules is called molecular assembly (MA). To date, all other approaches to experimentally address molecular complexity require the formula and connectivity of the molecule to be known.^20^ The MA^21,22^ for a molecule is computed by representing the molecule as a graph and performing an algorithmic search to find the shortest pathway to construct the graph by reusing previously made structures along the pathway; see Figure 1A. Thus, various constraints in the molecular graph are found along the pathway to quantify the complexity of the molecule.

In previous work, we used tandem mass spectrometry (MS/MS) for the experimental measurement of MA and were able to rank molecules in order of their complexity by placing them on a scale where molecules with experimentally measured MA greater than 15 were shown to be consistent biosignatures for life-detection, and the greater this number, the greater the likelihood that the molecule could only have been produced by biological or technology. Experimentally, over a range of high MA molecules, it was demonstrated that there exists a correlation between MS^2^ peaks and computed MA values.^23^ Herein, we developed experimental measurement strategies to infer molecular complexity using MA by using IR and NMR spectroscopies and expanded our understanding of inferring MA from mass spectrometry by using a new algorithm. Using both simulated and experimental data, we demonstrate that MA can be experimentally inferred over a wide range of complex molecules as well as mixtures. Additionally, we demonstrate that by combining multiple spectroscopic techniques into one measure, the MA prediction can be improved further.

Infrared spectroscopy (IR) is routinely used to confirm the presence of specific bond types in molecules by observing their characteristic vibrational energies in higher energy ranges (1500–3600 cm^–1^). Vibrational motion corresponding to those absorption bands is typically of a local nature, for example, a stretch vibration of one bond. In contrast, the lower energy (fingerprint) region 400–1500 cm^–1^ typically possesses a plethora of absorption bands, without direct easy interpretation toward structure elucidation.^24^ These modes include various collective motions, bending vibrations, and coupled modes. Since the number of different substructures increases with molecular complexity, we hypothesize that the number of unique absorption bands in the fingerprint region can be used to infer the complexity of organic molecules. Moreover, IR has previously been used to fingerprint complex molecular ensembles in their native natural environment.^25^

Nuclear magnetic resonance (NMR) spectroscopy provides resonance frequencies of magnetically inequivalent atom nuclei in the structure. The exact chemical shift of each nucleus of the same element depends on the effective magnetic field experienced by it, strongly influenced by its chemical microenvironments (affected by, for example, bond correlation via scalar coupling, or through space (de)shielding effects).^26^ NMR has been used in the past to analyze chemical space for fragment screening in drug discovery^27−29^ and characterizing structural complexity of compound classes.^30^ Advantageously, NMR has minimal solvent limitation, allowing the sample to remain in its native state/solvent if desired. NMR is uniquely equipped to address the structural diversity, as symmetric (magnetically equivalent) units in an isotropic environment (e.g., in a homogeneous solution) possess the same chemical shift (thus not creating duplicated resonances). Further, from the perspective of molecular assembly, the effect of symmetry and bond rotation on NMR spectra was hypothesized to provide near-equivalent resonances for duplicated fragments, even if not magnetically equivalent as a result of very similar chemical microenvironment experiences by the fragment. This represents the fact that assembled fragments may be utilized in multiple symmetric positions in a structure without having to “rebuild” them each time. Therefore, we hypothesize that the number of observed NMR resonances will reflect a degree of structural complexity.

Both IR and NMR will agnostically indicate the complexity of a molecule defined by MA since assembly theory states that MA utilizes unique irreducible motifs to construct the molecule that are indicated by the observed spectral features. This suggests that spectroscopy techniques that can quantify the properties of unique environments and molecular substructures should in principle produce a good correlation with MA. Thus, we hypothesize that with more unique bond types and atomic environments for a given molecule, the larger the number of peaks that should be found in IR and NMR spectra for that molecule; see Figure 1.

## Calculating Assembly Index from a Molecular Graph

The assembly index and associated minimal assembly pathways are calculated using an algorithm written in the Go programming language. In prior work,^23^ the assembly index was calculated using a serial algorithm written in C++ and yielded the “split-branch” assembly index, an approximation that provides a reasonably tight upper bound for the assembly index. In the split-branch approximation, it is not possible for a nonbasic object to contribute to the formation of multiple structures in an assembly pathway. That restriction allowed for a more efficient algorithm that could partition the molecules into separate parts and deal with them independently. The Go algorithm, subsequently developed and used in this work, is a faster algorithm that incorporates concurrency and can provide the exact assembly index (as opposed to the split-branch upper bound) if it can be calculated in a reasonable time. The process can also be terminated early to provide the lowest assembly index found so far, which has been found to be a good approximation of the assembly index in most cases.

**Figure 2:**
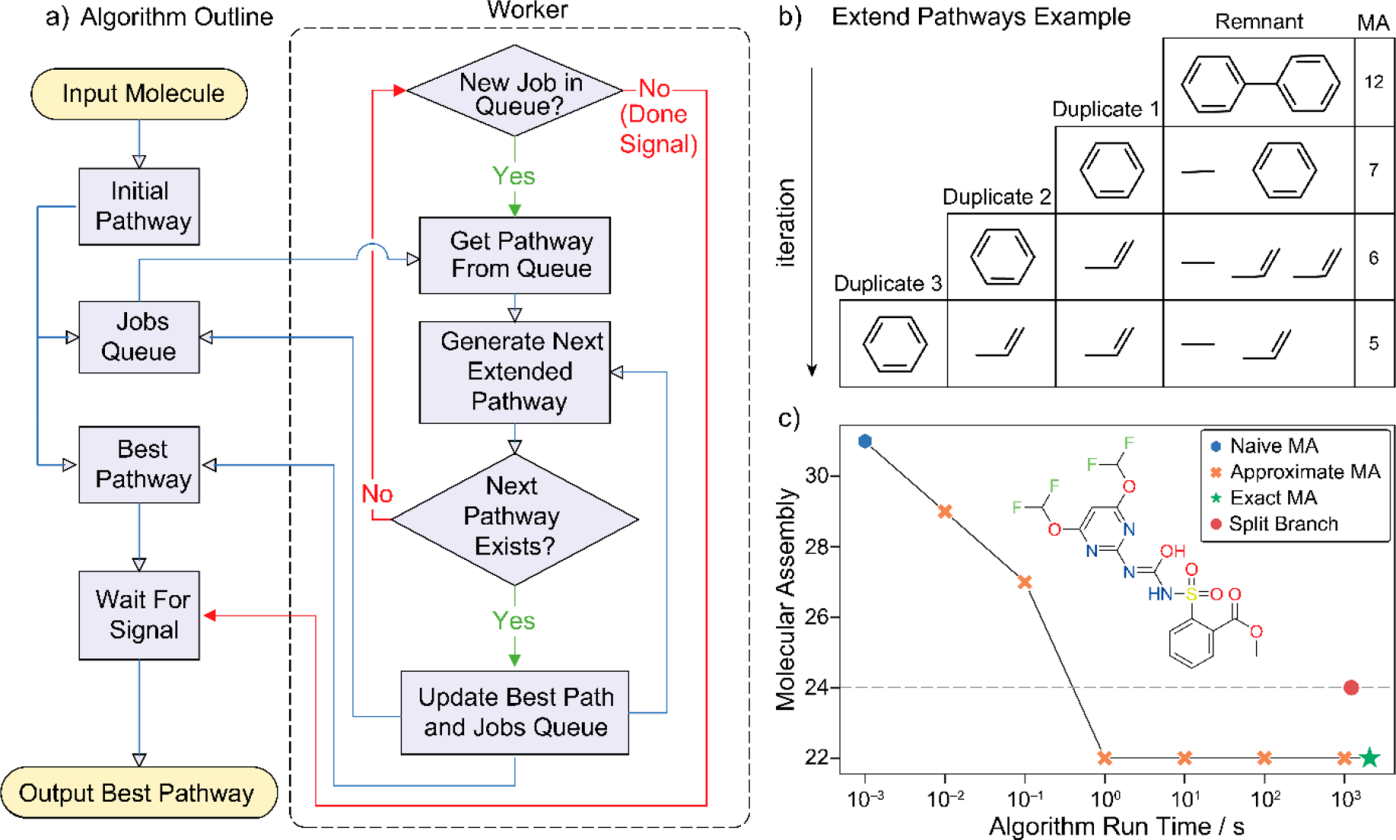
(a) The general structure of the Go assembly algorithm with a pool of workers extending pathways by queuing the pathways to be checked as jobs. Some features are omitted for brevity, such as branch and bound methods to improve efficiency. (b) A sequence of assembly pathways as processed by the Go algorithm. The top pathway is the starting pathway for the molecule shown, and each subsequent pathway is extended from the pathway above. Pathways are generally extended in multiple ways, and only one such sequence of extensions is shown here. (c) An example of MA values found over time for primisulfuron-methyl, run to completion, and approximated by stopping early at various stages prior. The new algorithm found pathways at the correct MA of 22 by 10 s, significantly before completion at ∼2064 s. The red circle shows split branch algorithm performance on the same molecule. The naïve MA (blue hexagon) is calculated trivially for pathways in which one bond is added at a time (placed illustratively at 10^–3^ s, as 0 s cannot be represented on the logarithmic scale).

The assembly index is calculated by iterating over subgraphs within a molecular graph and finding duplicates of that subgraph within the remainder of the molecule. For each of the matching subgraphs found, an assembly pathway can be represented by a duplicate structure and a *remnant* structure (for more details see the Supporting Information Section 1). The remnant structure comprises the original structure with one *duplicate* removed and the other “broken off”, which ensures that all structures on an assembly pathway that are duplicated will be first constructed. The process can then be repeated recursively with the remnant structure as an input, which may result in more pathways containing two duplicate structures and a smaller remnant. Thus, each pathway is represented by a sequence of duplicated structures and a remnant structure (Figure 2).

In this regard, it is important to point out that molecular assembly uses bonds as building blocks and not atoms. In order to determine the assembly index, we consider that a molecular graph with *N* bonds could be constructed in *N* – 1 steps by adding one bond at a time (the naive MA, or MA_naive_). Each duplicate structure of size *N*_dup_ allows us to add that structure in one step, reducing the number of steps compared to MA_naive_ by *N*_dup_ – 1. Thus, the MA for a particular pathway is MA_naive_ – ∑_dup_(*N*_dup_ – 1). For more details, see SI Section 1.

## Inferring Molecular Assembly Using Infrared Spectroscopy

**Figure 3:**
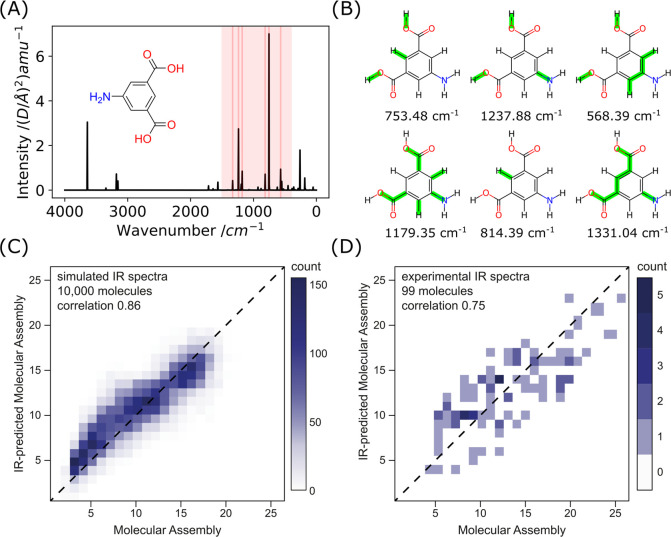
Inferring molecular assembly from infrared spectroscopy. (A) xTB-calculated IR spectrum of 5-aminoisophthalic acid with highlighted fingerprint region (400–1500 cm^–1^). (B) Example of the six most intense vibrational bands in the fingerprint region, demonstrating its collective-motion nature. (C) Molecular assembly vs IR-inferred molecular assembly estimated from the number of IR peaks in the fingerprint region (400–1500 cm^–1^) based on xTB calculation on 10,000 molecules (see eq 1). Correlation between the predicted and expected molecular assemblies is 0.86. (D) Molecular assembly vs IR-inferred molecular assembly estimated from the number of IR peaks in the fingerprint region (400–1500 cm^–1^) based on the experimental measurement on 99 molecules (see eq 2). Correlation between the predicted and expected molecular assemblies is 0.75.

As a first step, we computationally explore the potential for inferring molecular assembly from IR absorption. A set of 10,000 molecules was chosen uniformly from the data set published with a previous study^23^ of approximately 10^6^ molecules with MA. The new algorithm vastly speeded up the calculation, and we were able to sample chemical space by calculating the MA (previously called pathway assembly, calculated using a split-branch algorithm). This was done so that we calculated MA for ca. 650 molecules at each MA unit between 2 and 23 MA for each molecule with the new implementation.^31^ We calculated the IR spectra of the molecules using an extended semiempirical tight binding model implemented in xTB software including geometry optimization and calculating frequency resonances (see SI Section 2.3).^32,33^ For each spectrum, we estimated the total number of peaks in the fingerprint region (400–1500 cm^–1^) assuming a resolution of 2 cm^–1^. The number of peaks correlated significantly (Pearson correlation coefficient of 0.86) with the calculated MA, yielding a simple prediction function that was phenomenologically derived via linear regression: MA = 0.21 × *n*_peaks_ – 0.15 (eq 1). This observation corroborated our hypothesis that the number of absorption peaks in the IR fingerprint region reflects molecular complexity; see Figure 3.

Further, we expanded the study with experimental validation, using a set of 99 compounds selected to cover a large MA distribution (4–26). The experiments were performed by using diamond-attenuated total reflectance IR spectroscopy with a resolution of 2 cm^–1^. The obtained spectra were processed at 50% sensitivity and up to 80% transmittance threshold for selecting peaks using OMNIC software as the coarse filter against low-intensity noise in real spectra. The total number of IR peaks in the fingerprint region (400–1500 cm^–1^) correlated well with the compounds’ MA with a 0.75 correlation coefficient. This provided a handle for inferring the molecular assembly from an experimental IR using a simple linear function: MA = 0.45 × *n*_peaks_ – 2.3 (eq 2). For more details, see SI Section 3.

## Inferring Molecular Assembly from NMR Spectra

Most organic molecules (by definition) are composed of mainly carbon and hydrogen atoms; we hypothesized that ^13^C NMR is a practical technique to infer the molecular assembly of organic molecules. This was because the computation of molecular assembly is based upon bonds as building blocks, and NMR will be uniquely able to explore the connectivity within complex organic molecules by exploring and quantifying the types of carbon atoms present such as CH_3_, CH_2_, CH, and C, along with their relative connectivities. For the experimental measurement, the spectral width within which typically observed ^13^C nuclei resonances are found is relatively broad (∼200 ppm), and it is reasonable to assume that inequivalent nuclei of sufficiently different microenvironments would rarely possess the same resonance frequency within a resolution of 0.5 ppm. Further, we expect that magnetically nonequivalent, yet structurally very similar subunits with the exact environment in the nuclei vicinity will be found within the resolution width (see SI Section 2.2). Observing such overlap will reflect the unit’s similarity, and the peaks will not be overcounted as the corresponding substructures likely share the assembly space (the space of motifs that are used to construct the target) and do not contribute to the molecular assembly (e.g., repeating units of the polymer chain such as −CH_2_−).

Further information that can be experimentally extracted from the ^13^C NMR spectrum is the classification of the carbon nuclei by the number of attached hydrogens. Based on assembly theory, we hypothesize that the presence of carbons with no attached hydrogens (for clarity will be referred to as *quaternary*, note that this name will not be used exclusively for carbons with four different substituents, but for any carbon without connected hydrogen) reports most significantly on the molecular complexity, as such centers are highly connected to four different atoms but also can be connected to a range of different heteroatoms. Thus, these centers are hard to produce and require many constraints to construct them. Analogously, we hypothesize that the more hydrogens attached to the carbon, the less localized information it stores and, hence, contributes less to the molecular assembly. From the experimental point of view, the classification of carbon nuclei by the number of attached hydrogens can be experimentally achieved using standard DEPTQ-90 and -135 routines, which provide information about the number of hydrogens attached to the carbon via the hydrogen–carbon coupling.^34,35^

## Theoretical Investigation

**Figure 4:**
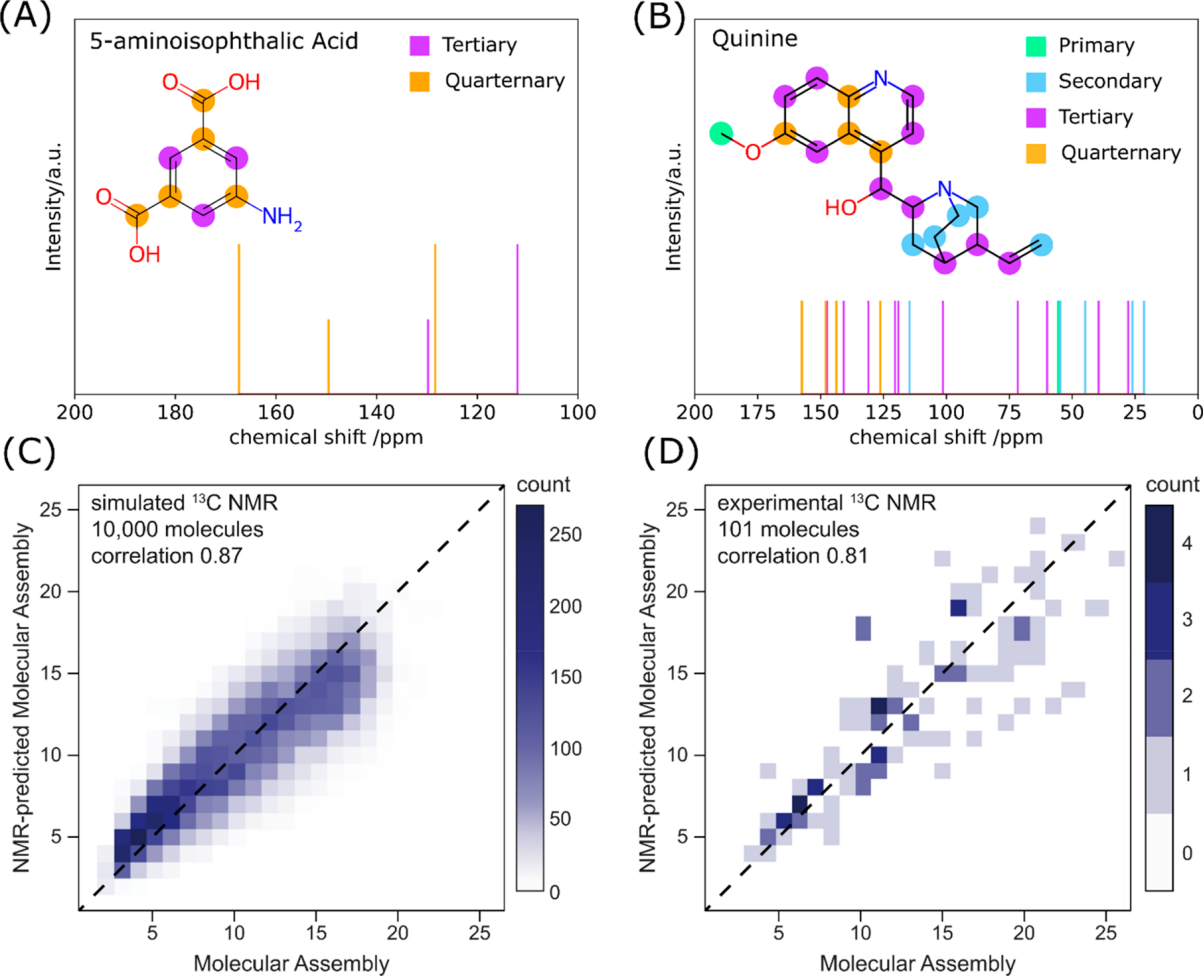
Inferring molecular complexity from ^13^C NMR spectra. (A and B) Predicted ^13^C NMR spectrum of 5-aminoisophathalic acid and quinine, with highlighted different types of carbons. (C) Molecular assembly vs NMR-inferred molecular assembly estimated from the number of different types of carbons (see eq 3) based on NMRshiftDB calculation on 10,000 molecules. The correlation between the predicted and expected molecular assembly is 0.87. (D) Molecular assembly vs NMR-inferred molecular assembly was estimated from the number of different types of carbons experimentally on 101 molecules, using the same model as in the theoretical set. The correlation between the predicted and expected molecular assemblies is 0.81.

To test our hypothesis, we examined a set of predicted ^13^C NMR spectra of 10,000 molecules (the same set as in the case of theoretical IR investigation). We have used the established predicting tool NMRShiftDB employing the hierarchical organization of spherical environments (HOSE) method.^36^ An example of NMR prediction for two molecules (5-aminoisophathalic acid (MA = 7) and quinine (MA = 16)) with various carbon atoms labeled is shown in Figure 4A,B. We classified the carbons by the number of hydrogens attached to them and summed the number of predicted peaks of a certain type assuming a bin width of 0.5 ppm. We performed multivariate linear regression (weighing out differently different types of carbons) and provided a model with a good correlation of 0.87; see Figure 4C. The formula for inferring the molecular assembly from the number of found peaks associated with individual carbon types was phenomenologically derived via linear regression to be MA = 1.3 × C + 0.8 × CH + 0.6 × CH_2_ + 0.3 × CH_3_ + 2.1 (eq 3), where C (quaternary), CH (tertiary), CH_2_ (secondary), and CH_3_ (primary) are the number of binned (by 0.5 ppm) ^13^C resonances of carbons with none, one, two, or three hydrogens attached, respectively. This observation on a large data set significantly corroborates our prediction that quaternary carbons possess the highest degree of constraints and have the highest potential to report on molecular complexity.

## Experimental Validation

For experimental validation, we have assessed 101 compounds, chosen to cover a range of molecular assembly (3–26) and structural diversity while being suitable for NMR measurement. We have acquired ^13^C NMR spectra and experimentally assigned the carbon type (C, CH, CH_2_, and CH_3_) via DEPTQ-90 and DEPTQ-135. The correct assignment was further cross-validated with ^1^H–^13^C HSQC since occasionally postprocessing of DEPTQ spectra can result in the inversion of the peaks’ phase. As the number of peaks is a simple and reliable measure directly comparable to the experimental observable property, we could test the trained model (eq 3) directly on independently chosen experimental molecules.

Testing the trained model provided a good correlation of 0.81; see Figure 4D. Allowing the change in the multivariate regression on the experimental set could provide an even better correlation of 0.86 (see SI Section 4); however, we have considered using the model train on a large data set as the more robust model, less biased by the sampling of the chemical space.

## Determining Molecular Assembly Using Tandem Mass Spectrometry

In our previous study, we demonstrated that tandem mass spectrometry can be used to estimate the molecular assembly of molecules directly from the peak count of MS^2^ spectra.^23^ As a generalized extension, herein, inspired by the construction of the assembly pathway of a molecule, we develop a new and robust algorithm to estimate molecular assembly utilizing multiple fragmentations of the molecule. The key idea is to construct a hierarchical tree structure by matching the fragment masses in the MS^*n*^ data with nodes representing the molecular fragments. This is analogous to the assembly contingent pathways^19^ representing the steps to construct the molecule from the molecular bonds as the fundamental building blocks. It is important to note that the tree structure does not necessarily represent the shortest path within the assembly space; however, it is crucial for inferring molecular assembly accurately from the mass spectrum. Within the tree structure of the fragmentation spectra, we compute the molecular assembly of all of the fragments from the mass and combine them to estimate the molecular assembly of the parent molecule. In the next sections, we explore the relationship between molecular weight and molecular assembly over a large data set and utilize a recursive algorithm to compute the molecular assembly of the molecule.

## Relation of Molecular Assembly and Molecular Weight

**Figure 5:**
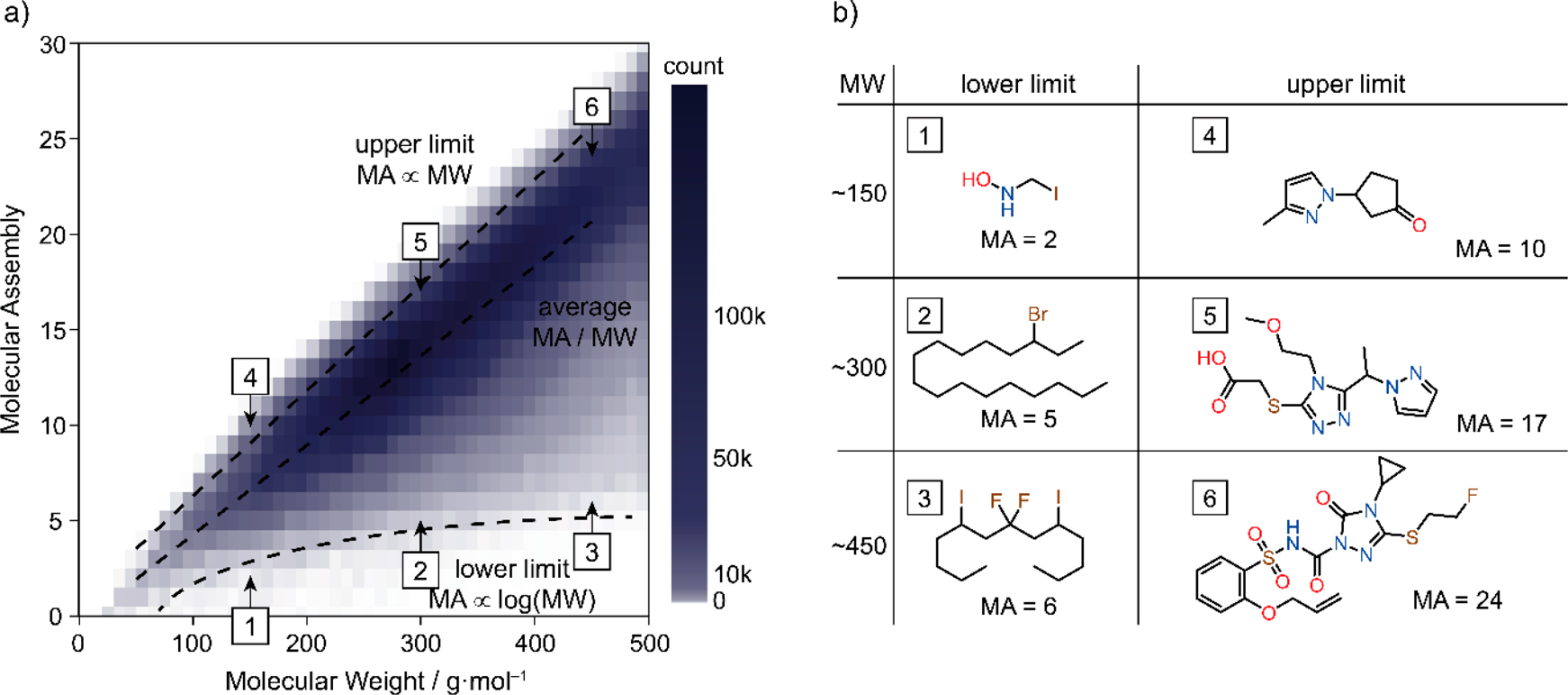
(a) Distribution of MA against molecular mass, based on 16.5M molecules sampled from the PubChem database. The upper limit is linear and the lower limit is approximately logarithmic in nature. The theoretical MA values were calculated with a 10 s cutoff. (b) Sample illustrating features of molecules in the MA/MW ranges. The characteristic features of lower MA molecules at a given molecular weight include the presence of periodic units, heavy elements, or both. The high MA molecules usually comprise higher heterogeneity with atoms of similar atomic weights.

Since both molecular mass and molecular assembly increase with an increasing number of construction steps along an assembly pathway, one intuitively anticipates that heavier molecules are likely to have a higher MA. On a large data set of 16.7 million molecules, sampled from the PubChem database,^37^ we compute MA using the assembly algorithm with a short cutoff of 10 s. In the absence of any further information such as MS^*n*^ spectra, the correlation between the MA and MW (MA = 0.047 × MW – 0.4) can be considered as a first-order approximation, suitable for inferring MA of heterogeneous organic molecules (Figure 5a). This proxy for the MA inference provides a reliable prediction for heterogeneous, nonsymmetric organic molecules, but not a general solution for molecules with repeating units or heavy elements. The empirical distribution of MA per MW follows an approximate skew-normal distribution, which can be described with fitted parameters as location parameter *loc* = 0.0539 × MW – 0.406, skewness α = −0.0083 × MW + 0.1, and scale *s* = 0.0074 × MW + 0.511 (see SI Section 7.1 for more details). The upper limits of the MA within the sample could be bound as an empirically found 99% percentile fit (MA_max_ = 0.055 × MW + 0.9), interpretable as a naïve MA of a molecule constructed from lighter elements with relatively similar atomic weights such as C, N, and O. On the other hand, a lower limit could be defined, in the case of a polymer constructed from single-type monomeric units, to be approximately proportional to the logarithm of the MW as MA_min_ ∝ log_2_(MW).^19^ Yet, given the heterogeneity of the chemical space and the presence of heavy atoms significantly lowering the expected MA, the lower limit is not strictly logarithmic. As clear from the sample, the low MA of molecules within each distribution (i.e*.*, with the same molecular weight) can be attributed to the presence of heavy atoms, or repeating units, i.e. structural reuse (Figure 5b).

## Inferring Molecular Assembly from Tandem Mass Spectrometry

**Figure 6:**
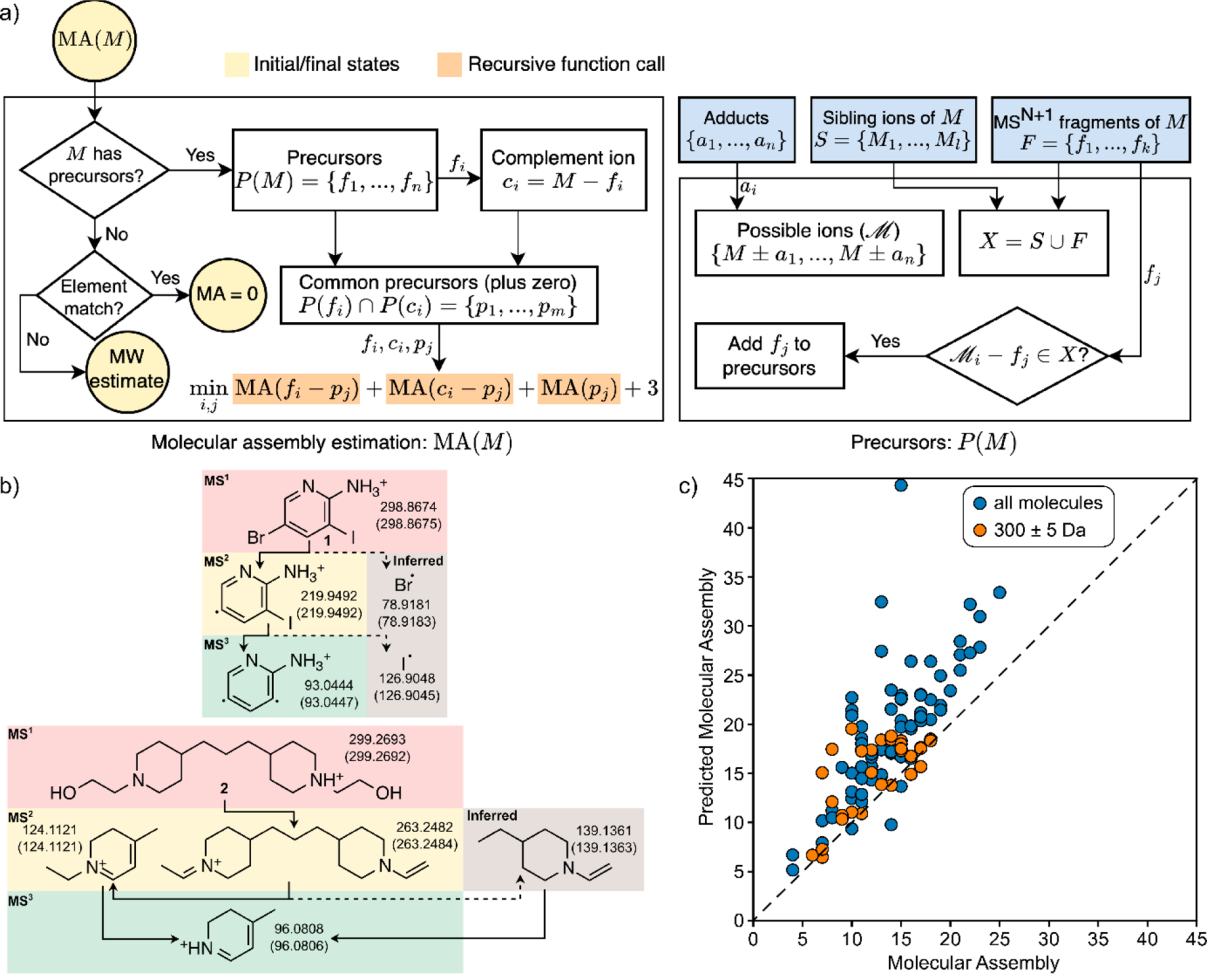
(a) The recursive algorithm for estimating molecular assembly from tandem mass spectrometry data. (b) Application of the recursive MA algorithm to resolve MA differences between molecules with similar molecular mass. Example of reduced MA based on the presence of heavy elements (bromine and iodine); example of reduced MA based on the presence of repeated structural features. (c) Molecular assembly vs recursive-MS^*n*^-inferred molecular assembly. Blue points represent the data set of 71 molecules sampled across the MA values. The orange points are 30 molecules, specifically chosen to cover a large range of MA, but within a very narrow molecular weight (300 ± 5 g/mol). The correlation coefficient is 0.73.

Multiple-level tandem mass spectrometry provides structured information about molecular fragments, which can be mapped to contingent pathways in the assembly space.^19^ Considering this, we developed a new recursive algorithm that combines molecular fragments based on their masses to create a tree and compute the MA of the molecule. As an input, we provide a mass spectrum, with multiple fragmentation events (MS^*n*^, where *n* indicates the number of consecutive ion fragmentations). The MA of a given ion is calculated by inferring all possible pathways to construct it from its daughter ions, applying the same process to each daughter ion. The chain of recursive search terminates whenever a daughter’s mass matches the monoisotopic mass of an element (MA = 0) or when there are no daughters present for a given ion, in which case the molecular mass approximation is used (Figure 6a). The range of possible MA of a fragment is predicted as a sample from normal distribution with location parameter *loc* = 0.074 × MW – 1.4 and scale *s* = 0.0074 × MW + 0.511. The scale parameter of the distribution was fitted on the large data set from the PubChem database (see previous section), and the *loc* parameter was parametrized to describe well small molecular fragments (see SI Section 7.2 for further discussion). To test the recursive fragmentation algorithm, we experimentally assessed 101 molecules selected to cover a wide range of MA (4–24) while suitable for MS measurement. This set included 30 molecules, selected to have nearly the same MW (300 ± 5 g/mol) and various MA in the range of 5 to 17. Similar MW molecules were chosen to demonstrate the capability of the algorithm to distinguish high and low MA molecules using MS^*n*^ with similar characteristic MW values. The fragmentation events were carried out up to MS^5^, where possible. The test sample of molecules with similar MW is a particularly difficult task for inferring MA by MS, as the MW prediction would fail to distinguish the difference in complexity among the samples. Examples of determining the molecule MA from this set based on matching fragments or finding heavy elements are highlighted in Figure 6b. The correlation coefficient between the predicted and expected values on the total sample was found to be 0.73 (Figure 6c).

## Combining Analytical Techniques for Molecular Assembly Inference

**Figure 7:**
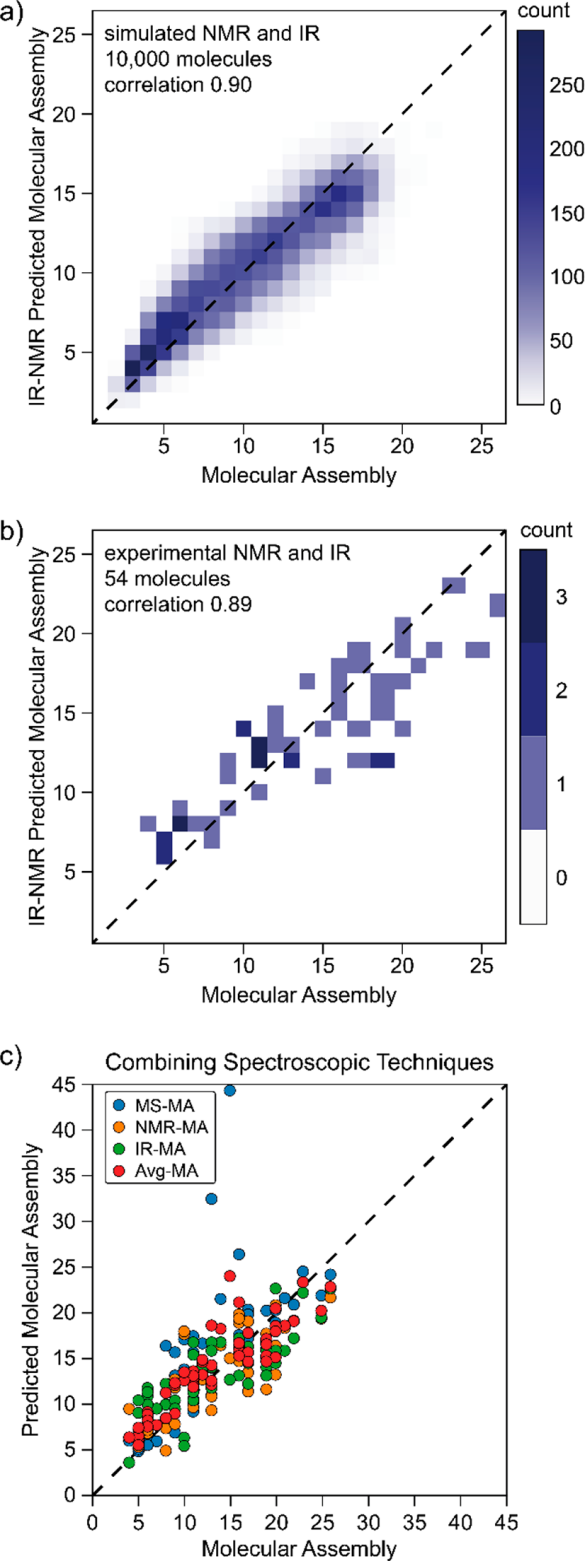
(A) Molecular assembly vs combined IR- and NMR-inferred molecular assembly (using weights of 0.55 and 0.45 from NMR and IR, respectively) based on 10,000 calculated spectra showing an increased correlation of 0.90. (B) Molecular assembly vs combined IR- and NMR-inferred molecular assembly (using weights of 0.7 and 0.3 from NMR and IR, respectively) based on 54 experimental spectra showing an increased (relative to the individual components) correlation of 0.89. (C) Molecular assembly vs individual and combined IR-, NMR-, and MS-inferred molecular assembly based on the 54 molecules. Note that due to experimental limitations, multilevel MS fragmentation data were available for only 10 compounds; for the rest the MS part of the MA inference was performed based solely on the exact ion mass approximation. The correlation coefficient for the combined techniques is 0.88.

Molecular constraints are probed by different physical interactions depending on the spectroscopic techniques, which independently have been shown to correlate with MA. In general, due to different limitations in the considered techniques (NMR, IR, and MS), individual spectral features of a molecule of unknown origin can be biased. For example, MS/MS fragmentation is biased by the strengths of the different chemical bonds, which the molecular assembly calculation does not consider. Similarly, ^13^C NMR spectroscopy might not fully reflect the MA should the constraints be realized through heteroatoms; further diastereotopic carbons can be overcounted although considered equivalent. The IR fingerprint region can contain overtones of the functional groups, causing the peak overcount. All examples listed herein responsible for variance in the correlation with the MA have principally different physical interactions. We, therefore, hypothesized that a combination of the analytical techniques can increase confidence in the MA inference; see Figure 7.

On the set of 10,000 calculated NMR and IR spectra, we have examined our hypothesis that combined information can provide a more reliable MA prediction. We have used the same models (eqs 1 and 3) for the individual spectroscopic techniques and allowed them to optimize for their relative weighting. The combined model provided a higher correlation of 0.91 using the weighted average of 0.55 × NMR and 0.45 × IR inferred MA; see Figure 7A. Further, we have validated this approach on the available intersection of the experimental NMR and IR data, comprising 54 molecules. The combined model provided a higher correlation of 0.89 using a combination of 0.7 × NMR and 0.3 × IR inferred MA; see Figure 7B. Lastly, we have explored the combination of all three techniques to infer MA. Where the experimental data set was available, we used a recursive algorithm applied to MS^*n*^ fragmentation data to infer MA as accurately as possible. In cases in which the data were not available, we approximated inference by the linear correlation to the exact mass of the molecule (Figure 7C). Although the average value might not always provide a better estimate than certain individual components, it provides a more robust prediction, should no information about the sample be available. Such a difficult case is for symmetric polyaromatic heterocycles, where building blocks are reused, yet the strength of the multiple aromatic bonds does not provide (under the condition of collision-induced fragmentation used in this study) useful fragments to report on it. Spectroscopic techniques, on the contrary, will provide correct prediction, as the symmetry will be reflected in simplified spectra. This observation highlights the utility of inferring the MA of unknown species using multiple experimental techniques and acquiring an average of their MA predictions.

An additional challenge for inferring the complexity of an unknown sample is to consider a mixture of compounds. To address this issue with spectroscopy, we demonstrated the use of ^13^C DOSY spectroscopy to experimentally deconvolute the mixture of chemical resonances to their individual components. We investigated ^13^C DOSY spectroscopy on mixtures, separating individual compounds via their diffusion coefficient.^38^ Together with the experimental assignment of the carbon types, we could predict the molecular assembly of each component in the mixture using the same logic as for the individual compounds.

An example of such a workflow is assessing a mixture of 5-aminoisophathalic acid and quinine, which yielded a prediction of the molecular assembly to be 8 and 19, in reasonably good agreement with the expected real value of 7 and 16, respectively. For more details, see SI Section 6.1. The work here shows that the general concept of measuring molecular complexity as a function of the number of different parts in a molecule using spectroscopic measurements gives a very strong correlation with the theoretical assembly complexity. This is important since it means we can use experimental measurements on environmental samples to read out the amount of selection and evolution that the samples have been subjected to, making this approach suitable for the search for new life on and beyond Earth.

## Conclusion

We have demonstrated on a set of 10,000 simulated and approximately 100 experimental IR and NMR spectra that it is possible to predict the MA of compounds without structural elucidation. On a set of approximately 100 molecules, we have demonstrated a novel algorithm interpreting multilevel tandem mass spectrometry to infer MA. This experimental data set included 30 molecules with nearly the same molecular mass, testing the algorithm to differentiate MA based solely on the MS^*n*^. All of the above-mentioned methods are particularly useful for molecules of unknown origin and cases when a fast metric for probing complexity is required. In the case of IR, the constraints and molecular complexity are reflected by the number of peaks in the fingerprint region, and their simple summation can be used to predict molecular complexity. In NMR, we have shown that the weighted sum of the number of carbon resonances, sorted by the number of hydrogens attached to them, provides a good prediction of MA. We found that the fewer hydrogens attached to the carbon, the higher the weight that it possesses for the MA prediction. This finding corroborates our interpretation based on assembly theory that the quaternary carbons effectively encode the most information, whereas the primary carbons, which have more hydrogen atoms, are least encoded and hence contribute less to the molecular assembly. Furthermore, we provided a new algorithm examining multilevel tandem mass spectrometry to infer MA as a function of matching fragments or identifying the presence of heavy elements. Finally, we have demonstrated the possibility of addressing the complexity of the components in mixtures using ^13^C DOSY, deconvoluting the ^13^C NMR signals to their individual compounds. These findings are of particular significance for the development of missions exploring the extent of life on Earth and in our solar system.^39^ For example NASA has already managed to put several mass spectrometers on Mars,^40^ and several mass specs have been in the solar system including on the Cassini probe, which visited Saturn and Enceladus.^41^ Dragonfly is set to visit Titan, launching in 2026 and arriving in 2034, which is important since it will be a mobile mass spectrometer that flies around Titan.^42^ In the area of functional molecules discovery for drug discovery and new materials, maximizing molecular complexity holds a whole host of new opportunities for molecular design of molecules that combine multiple functional motifs capable of many different jobs, including the encoding of information.

## Experimental Section

### Infrared Experimental Setup

IR spectra were acquired on a Thermo Scientific Nicolet iS5 with a Specac Golden Gate Reflection Diamond ATR System. All data were processed with a Thermo Scientific OMNIC 8.3.103. All samples were measured in the native state at room temperature (solid state, unless liquid at room temperature).

### NMR Experiment Setup

NMR data were acquired on a Bruker Ascend Aeon 600 MHz NMR spectrometer with a DCH cryoprobe (^13^C + ^1^H channels) at 300 K unless otherwise stated, in which case a room temperature BBFO probe head (^1^H + ^19^F–^183^W channels) was used. ^1^H NMR spectra were acquired using 16 scans, a spectral width of 20 ppm, and a relaxation delay of 2 s. Spectra on the ^13^C channel were acquired with a spectral width of 200 ppm. The ^13^C NMR spectra were acquired by using 16 scans and a relaxation delay of 0.8 s. The DEPTQ routines were carried out using 16 scans and a relaxation delay of 1 s. The ^13^C DOSY spectra were acquired using 256 scans, a relaxation delay of 8 s, and a 500–1000 μs gradient pulse. All spectra were processed using Bruker Topspin 3.6 and Mestrenova 14.1.1. The spectra were phase and baseline corrected and calibrated relative to the residual solvent peak. Residual solvent peaks were not included in resonance counts. Unless otherwise stated, samples were prepared in DMSO-*d*_6_ at the concentration stated in the Supporting Information.

### MS Experiment Setup

Tandem mass spectrometry experiments were carried out up to the MS^5^ level on a Thermo Scientific Orbitrap Fusion Lumos Tribrid system via direct injection of samples dissolved in acetonitrile (details in SI). Following the acquisition, raw vendor outputs were converted to mzML using ProteoWizard (no filters were applied) for consumption by the recursive MA algorithm.

## Data Availability

The molecular assembly calculator called *AssemblyGo* was written in GO programming language (https://github.com/croningp/assembly_go). The codes used for processing data and further details can be found on Github https://github.com/croningp/molecular_complexity and https://github.com/croningp/RecursiveMA.

## Supporting Information Available

The algorithm for calculating molecular assembly for molecules, the details of the theoretical calculations for NMR, infrared (xTB and DFT simulations), sample preparations for the experimental data collection, experimental IR and NMR data, the regression analysis, mixture analysis (PDF)
Transparent Peer Review report available (PDF)
