# Supplementary material for: Investigating and Quantifying Molecular Complexity Using Assembly Theory and Spectroscopy
Source: ACS Cent Sci. 2024 Apr 18;10(5):1054–64. doi: 10.1021/acscentsci.4c00120 (PMC11117308; doi:10.1021/acscentsci.4c00120)
Supplement: Supplementary file 1 — oc4c00120_si_001.pdf [file oc4c00120_si_001.pdf]

Supplementary Information for:

Investigating & Quantifying Molecular Complexity using Assembly Theory and Spectroscopy

Michael Jirasek[†1], Abhishek Sharma[†1], Jessica R. Bame[†1], S. Hessam M. Mehr[1], Nicola Bell[1], Stuart M. Marshall,[1] Cole Mathis[1], Alasdair Macleod,[1] Geoffrey J. T. Cooper[1], Marcel Swart[2,3], Rosa Mollfulleda[2], Leroy Cronin*[1]

**Lee.Cronin@glasgow.ac.uk*

[1] *School of Chemistry, The University of Glasgow, University Avenue, Glasgow G12 8QQ, UK.*

[2] *University of Girona, Campus Montilivi (Ciencies), c/M.A. Capmany 69, 17003 Girona Spain*

[3] *ICREA, Pg. Lluis Companys 23, 08010 Barcelona, Spain*

Contents

1 Calculating Assembly Index From Molecular Graph S2
  1.1 Algorithm Description S2
2 Theoretical calculations for IR and NMR S4
  2.1 Database and sampling S4
  2.2 NMR Prediction S8
  2.3 Infrared Spectroscopy – xTB simulations S10
  2.4 Infrared Spectroscopy – DFT simulations S17
3 Experimental Infrared Spectroscopy S22
4 Experimental NMR S26
  4.1 Sample Preparation details S26
  4.2 NMR Experimental Parameters S26
  4.3 Classification of the Carbon Types S27
5 Combing IR and NMR data S32
6 Mixture Analysis S36
  6.1 NMR S36
7 Mass Spectrometry S38
  7.1 Theoretical Calculations S38
  7.2 Recursive MA algorithm S41
  7.3 Experimental Mass Spectrometry S44
8 References S46

1 Calculating Assembly Index From Molecular Graph

1.1 Algorithm Description

The assembly index, and associated minimal assembly pathways, are calculated using an algorithm written in the Go programming language. In prior work,(1) the assembly index was calculated using a serial algorithm written in C++, and yielded the "split-branch" assembly index, an approximation that provides a reasonably tight upper bound for the assembly index. The Go algorithm used in this work is a faster algorithm that incorporates concurrency, and can provide the exact assembly index if it can be calculated in a reasonable time. The process can also be terminated early to provide the lowest assembly index found so far, which has been found to be a good approximation for the assembly index in most cases.

The assembly index is calculated by iterating over subgraphs within a molecular graph, and finding duplicates of that subgraph within the remainder of the molecule. For each of the matching subgraphs found an assembly pathway can be represented by a duplicate structure and a remnant structure. The remnant structure comprises the original structure with one duplicate removed, and the other "broken off", which ensures that all structures on an assembly pathway that are duplicated will be first constructed (**Fig. S1**).

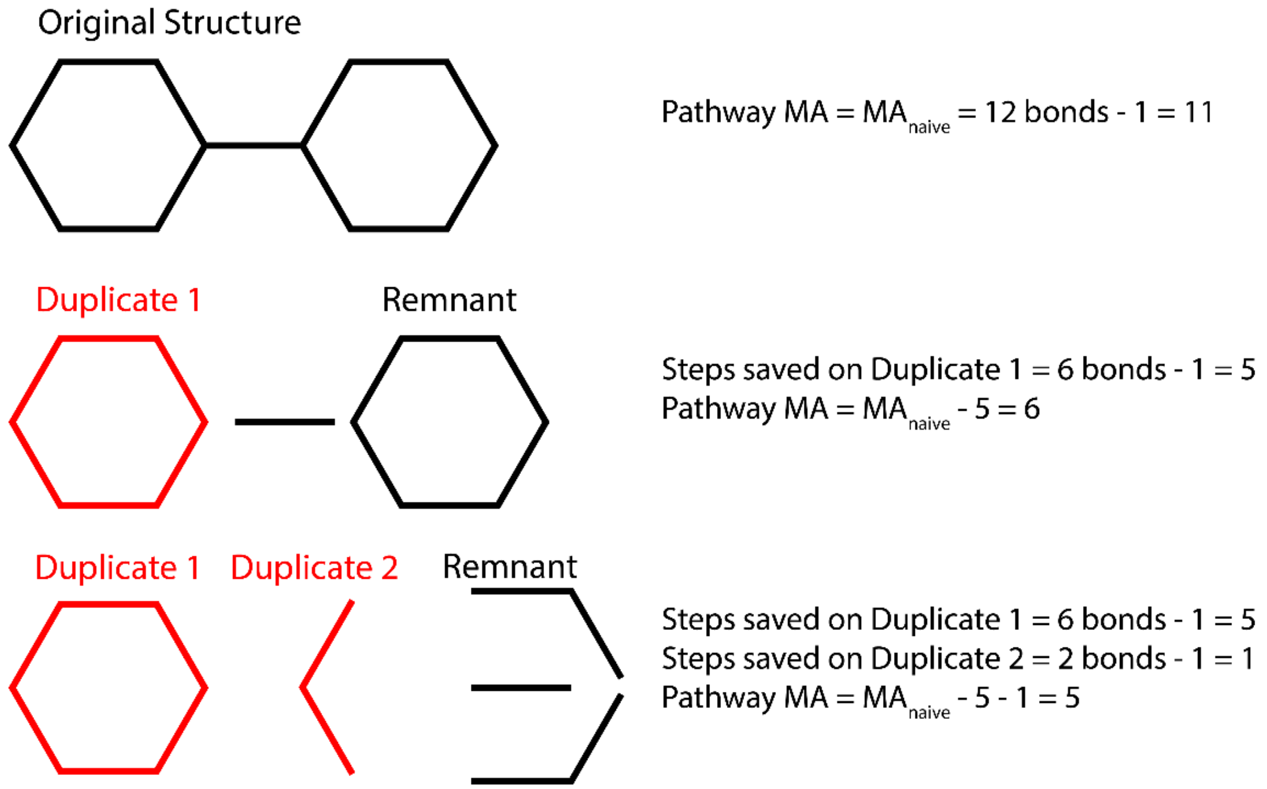

Fig. S1. Illustration of the process used by the assembly algorithm to extend an assembly pathway. At each step (top to bottom) a duplicate is found (red) and stored separately. The remnant is the remaining part of the structure, but with the matching duplicates separated. The process can then be repeated recursively on the remnant.

The process can then be repeated recursively with the remnant structure as an input, which may result in more pathways containing two duplicate structures and a smaller remnant. Thus each pathway is represented by a sequence of duplicated structures and a remnant structure. In order to determine the assembly index, we consider that a molecular graph with N bonds could be constructed in $N-1$ steps by adding one bond at a time (the naive MA, or MA_{naive}). Each duplicate structure of size N_{dup} allows us to add that structure in one step, reducing the number of steps compared to MA_{naive} by $N_{dup}-1$. Thus the MA for a particular pathway is $MA_{naive} - \sum_{dup}(N\_dup - 1)$.

Concurrency is implemented through a worker pool, with each worker iterating over the subgraphs of a particular pathway and placing generated extended pathways into a jobs queue to be picked up and extended by other workers. In order to prevent unbounded resource use, the jobs queue size is limited, and if full a worker will process generated pathways in a depth-first fashion until there is space in the queue, before resuming the breadth-first search. The algorithm has some branch and bound methods to reduce the search space (it will not extend pathways that cannot have lower MA than the lowest found so far), and can be terminated early to output the best pathway found so far. The approximation through stopping early has been found to output values at or close to the actual assembly index fairly quickly (**Fig. S2**).

The subgraph iteration process is based on,(2) and the subgraph matching functions are based on processes used in Nauty.(3) The overall algorithm concept is similar to the exact MA algorithm we published previously,(4) but with substantial improvements in terms of performance.

Molecular assembly can be expected to correlate with molecular weight. This is because there are upper and lower bounds for molecular assembly indices that scale with the number of bonds in the molecule. The trivial upper bound for the assembly index relates to pathways where one bond is joined at a time without any leverage of duplication (so the assembly index is equal to the number of bonds minus one). A basic lower bound can be determined by considering that the quickest way to increase the size of a structure using an assembly pathway is to take the largest structure created so far and combine it with itself, essentially doubling the size at each step. For example, a structure of 8 bonds cannot be made in less than 3 joining operations, and in general, a structure of N bonds has a lower bound on the assembly index of $\log_2(N)$. Both these bounds increase with the number of bonds, and since the number of atoms and hence the molecular weight tends to increase with the number of bonds, we can expect the assembly index to increase with the molecular weight.

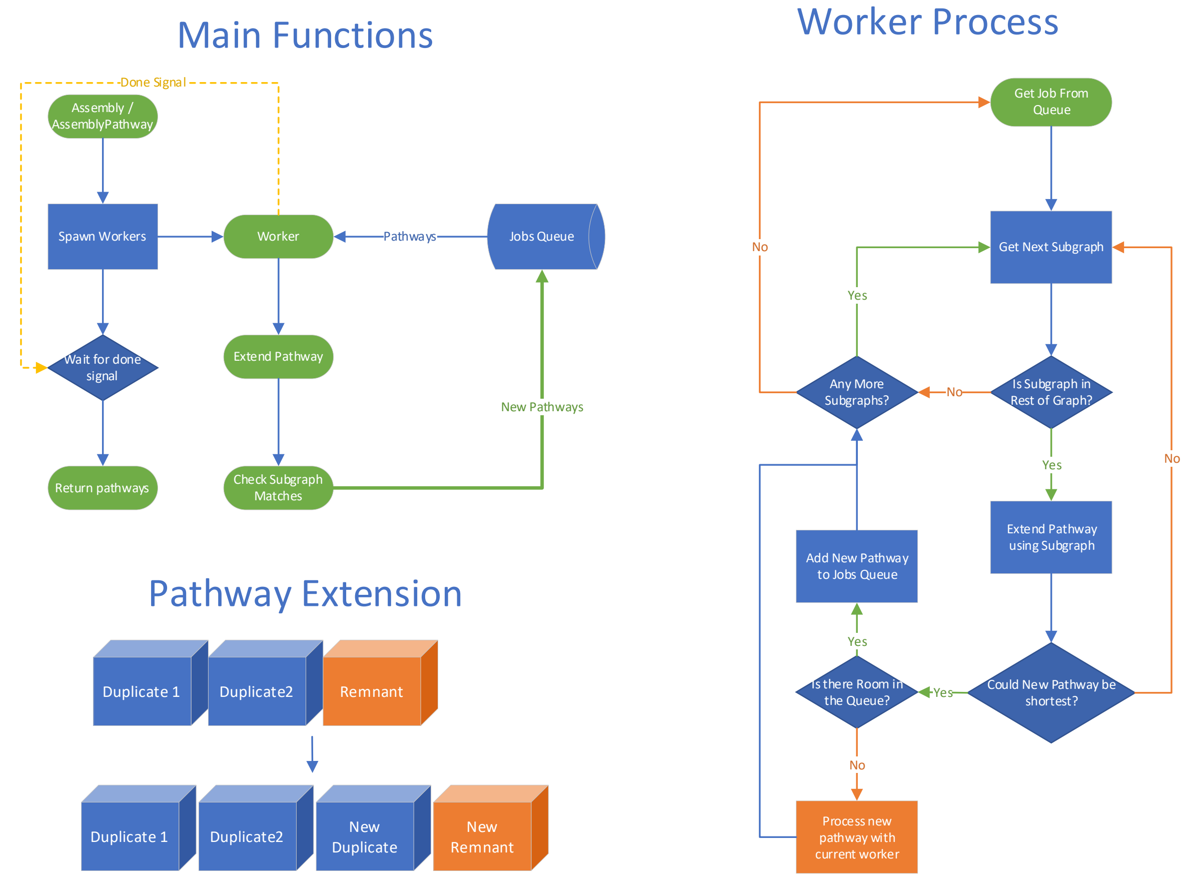

Fig. S2: Outline of algorithm process (top left). Illustration of extension of a single pathway (bottom left). Outline of process for single worker (right).

2 Theoretical calculations for IR and NMR

2.1 Database and sampling

The calculation and theoretical basis for Molecular Assembly (MA) calculation are described in detail in our previous work.(1, 5) Most of the analysis was performed using Python 3 and Mathematica 12. The Molecular Assembly calculator called *AssemblyGo* was written in GO programming language (https://github.com/croningp/assembly_go). The codes used for processing data and further details can be found at https://github.com/croningp/molecular_complexity.

To study the relationship between the MA and physically measurable properties, we used a previously published database of compounds for which the MA was calculated (~2.2M compounds). In order to address the molecular complexity of organic molecules, and given that we try to address the molecular complexity through carbon-sensitive ^{13}C NMR, hence, a relatively high abundance of carbon is essential. We filtered the compounds to have at least 50% and not more than 85% of the heavy atoms as carbons and must contain at least 4 carbon atoms. Such a filtered database contained *ca.* 0.77 million compounds. The range of the previously calculated Molecular Assembly (called Pathway

Assembly, using the split-branch algorithm) was found in between 3–25. The distribution of MA in the database is not uniform with the highest counts between 8-12 see **Fig. S3**.

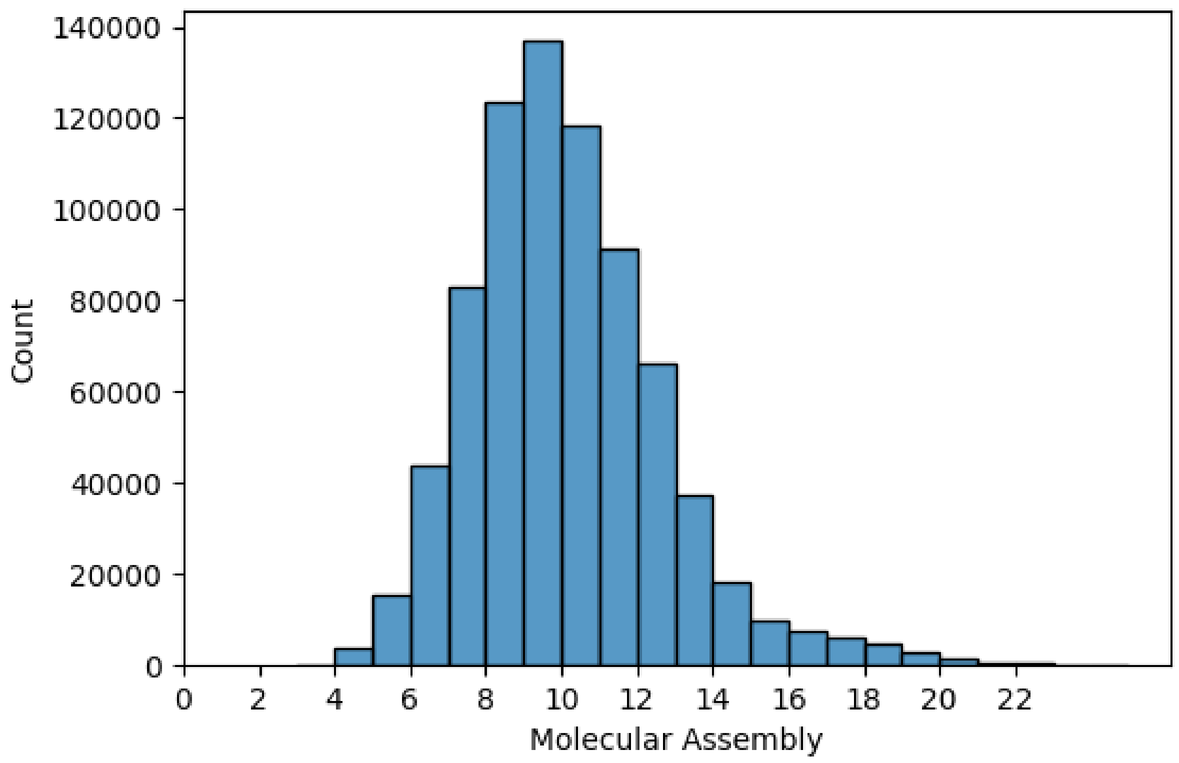

Fig. S3. Distribution of molecules over the Molecular Assembly from the previous dataset. The Fig shows the histogram of MA (previously called Pathway Assembly) distribution in the available dataset, containing ~0.77 million compounds with 50–85% carbons as heavy atoms, originating from the previously published dataset.(1)

The MA distribution of the compounds filtered from the previously published(1) database reflects synthetic availability (as the compounds originate from the published Reaxys database) and our previous capacity to reliably calculate MA using the split-branch algorithm (compounds for which it was assumed it would be impossible (at the time) to calculate MA reliably were rejected from the database). To assess the characteristic relationship between the MA with the spectroscopic techniques, we sampled 10,000 compounds uniformly across the MA range, up to 629 compounds per MA. The sampling was performed on the subset of which both theoretical NMR and IR data could be calculated using the simulation tools discussed in the later sections. For those compounds, the MA was recalculated using the newly developed assembly algorithm AssemblyGo which provides more accurate estimates at faster timescales, generally leading to estimating the assembly index to be lower by 1 or 2 relative to the original value (previously calculated Pathway Assembly). The distribution of the molecules over the MA range with newly calculated MA is shown in **Fig. S4**.

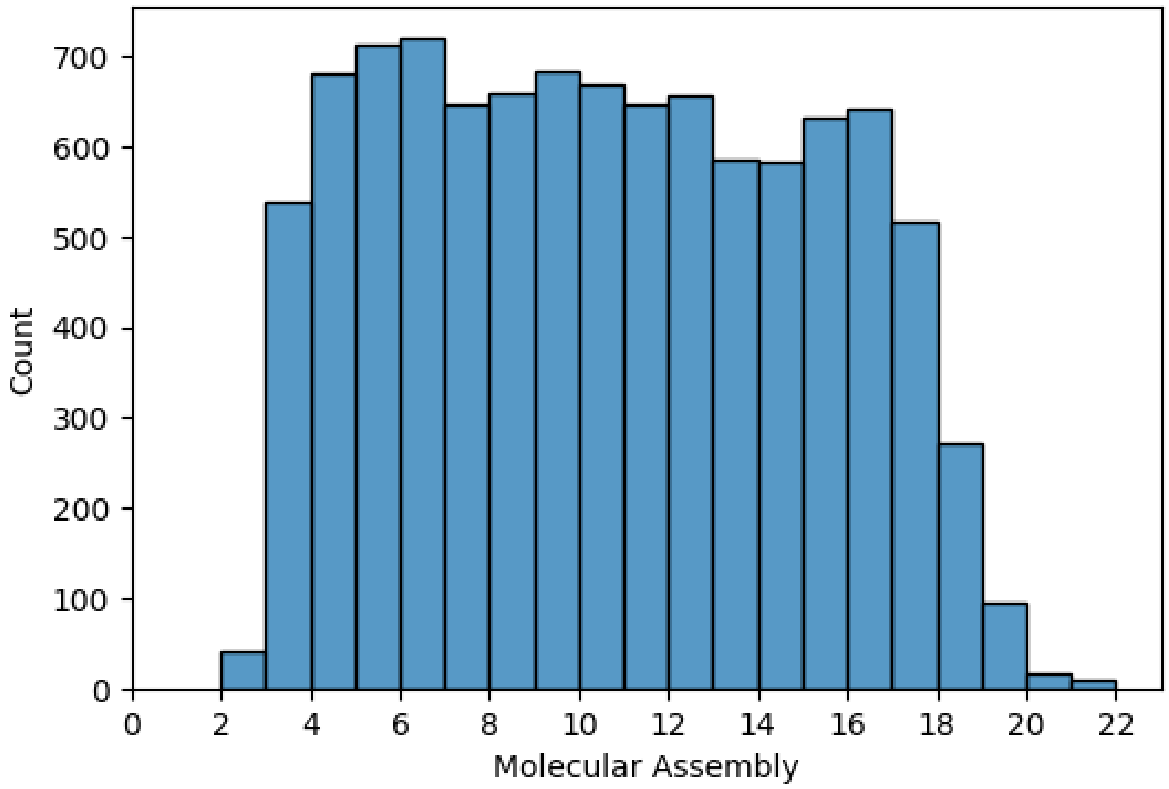

Fig. S4. Histogram of distribution in the sample of 10,000 molecules to cover uniformly the range of MA (recalculated values using a more accurate algorithm).

A representative subset of the molecules in the dataset over the range of MA values is shown in **Fig. S5** on the following page.

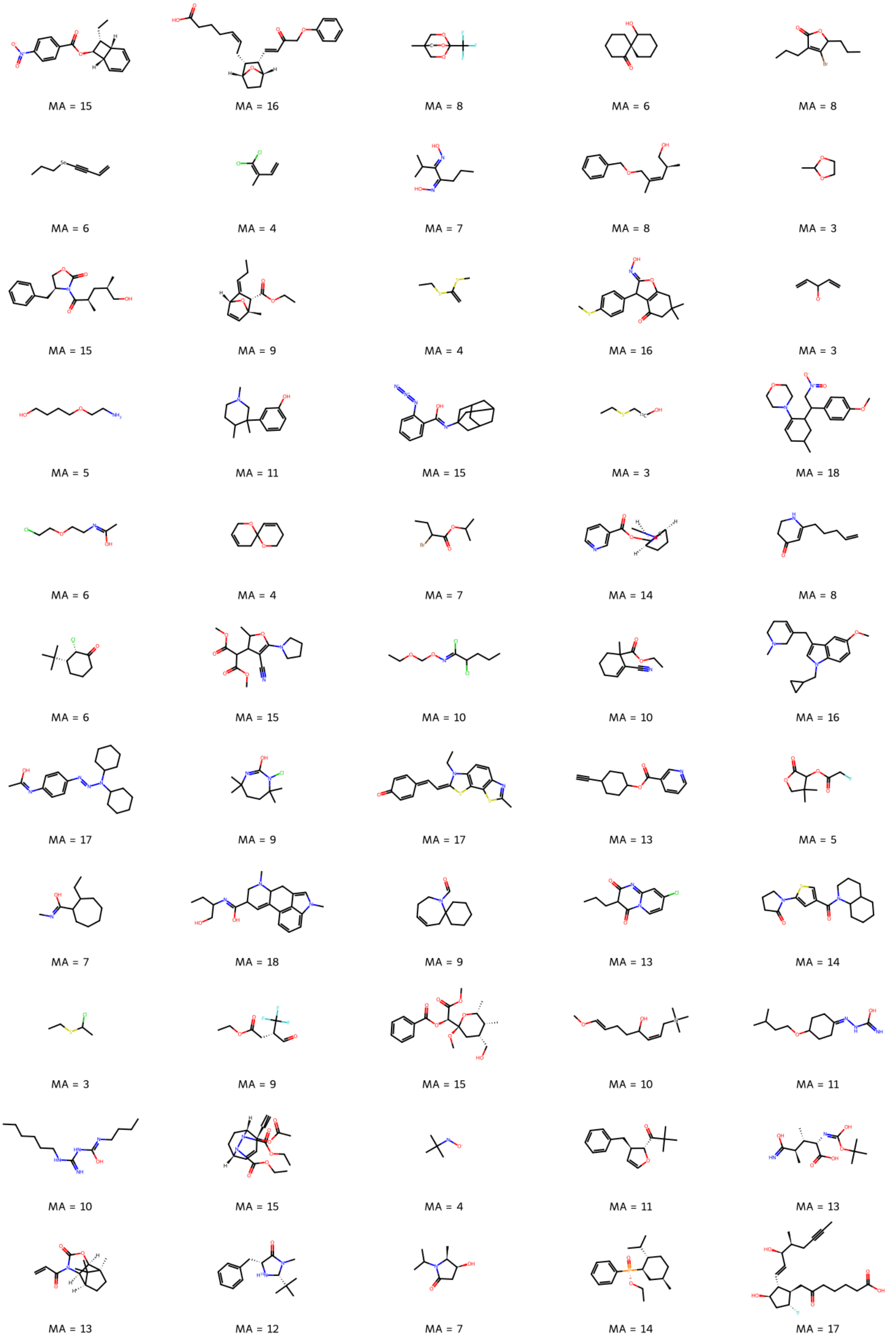

Fig. S5. Example of 55 compounds sampled from the database of 10,000 compounds used in the theoretical study.

2.2 NMR Prediction

The ^{13}C NMR spectra were predicted using *nmrshiftdb2* tool.(6) The corresponding chemical shifts were grouped by the type of carbon (primary (CH_3), secondary (CH_2), tertiary (CH) and quarternary (C)). Such sorting was performed by Python script using the rdkit(7) tool to estimate the number of hydrogen atoms attached. Further, the number of chemical shifts was binned, applying the minimum 0.5 ppm chemical shift difference (i.e. resonances within the 0.5 ppm were considered as a single peak for analysis).

The importance of an actual ^{13}C NMR measure/prediction instead of the sole counting of the number of chemically non-equivalent carbons in structure can be demonstrated on a large dataset of ~1.1 million compounds (allowing all compounds with more than 4 carbons and no constraints on the C content). This set was analysed by both NMR prediction, as well as by counting the number of nonequivalent carbons (for simplicity, the carbons were not classified by the type) (**Fig. S6**). Also, note that considered assembly index values are based on the old database that used the previous algorithm which is relatively less accurate. The potential outliers deviating from the linear trends highlight the utility of the actual NMR measure (as an oriented oligomer possesses plenty of non-equivalent carbons, yet of very similar chemical shift).

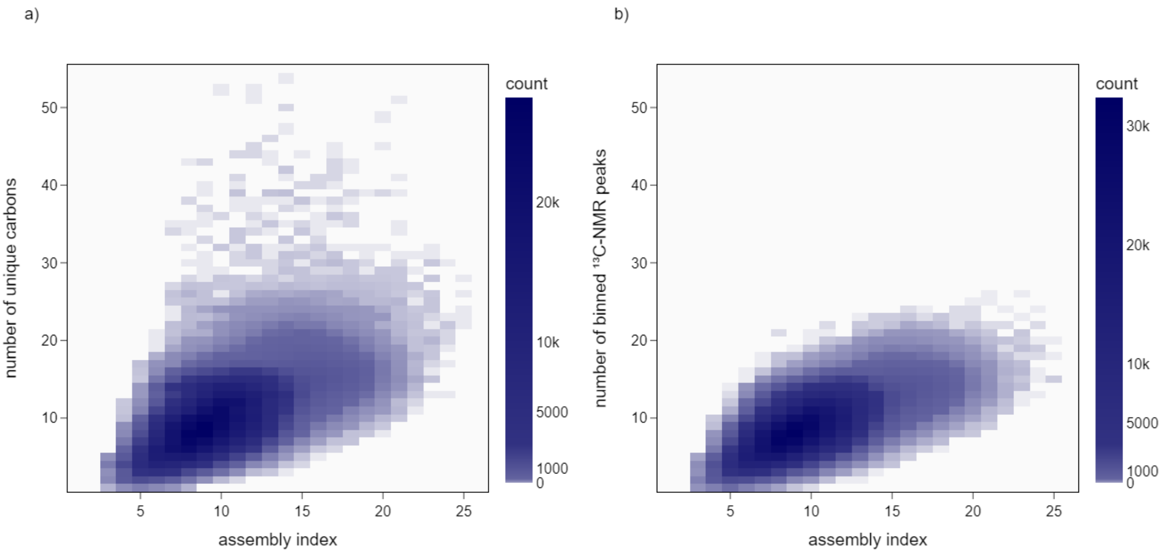

Fig. S6. **Analysis of *ca.* 1.1 million compounds.** a) Molecular Assembly (assembly index) *vs.* the number of unique carbons. b) MA *vs.* the number of predicted 0.5 ppm binned ^{13}C NMR resonances. Note that the z-axis (histogram count) is scaled logarithmically with base 10 to emphasise even the very uncommon cases.

On the sample of 10,000 molecules, whose MA was recalculated using the new algorithm, we used multivariate fit of the number of 0.5 ppm binned resonances of C, CH, CH_2 and CH_3 carbon resonances. For the fit, the *statsmodels.api.OLS* module in Python was used (**Fig. S7**).(8)

```
                    Results: Ordinary least squares
==================================================================
Model:              OLS              Adj. R-squared:     0.753
Dependent Variable: y                AIC:                44452.0003
Date:               2023-02-07 15:52 BIC:                44488.0520
No. Observations:   10000            Log-Likelihood:     -22221.
Df Model:           4                F-statistic:        7607.
Df Residuals:       9995             Prob (F-statistic): 0.00
R-squared:          0.753            Scale:              4.9869
----------------------------------------------------------------------
           Coef.     Std.Err.       t       P>|t|     [0.025    0.975]
----------------------------------------------------------------------
x1         1.3172      0.0135    97.2754    0.0000    1.2907    1.3438
x2         0.7996      0.0113    70.5589    0.0000    0.7774    0.8218
x3         0.6451      0.0118    54.6230    0.0000    0.6220    0.6683
x4         0.2620      0.0254    10.3091    0.0000    0.2122    0.3119
const      2.1549      0.0612    35.2323    0.0000    2.0350    2.2748
------------------------------------------------------------------
Omnibus:             269.066      Durbin-Watson:         1.546
Prob(Omnibus):       0.000        Jarque-Bera (JB):      292.973
Skew:                0.399        Prob(JB):              0.000
Kurtosis:            3.260        Condition No.:         16
==================================================================
```

Fig. S7. Print output from the multivariate fit of MA = x_1×C + x_2×CH + x_3×CH_2 + x_4×CH_3 + *const.*; using *statsmodels.api.OLS* in python.(8)

The best prediction of MA based on the NMR data is given by:

$$\mathrm{MA} = 1.32 \times \mathrm{C} + 0.80 \times \mathrm{CH} + 0.65 \times \mathrm{CH_2} + 0.26 \times \mathrm{CH_3} + 2.15 \qquad \textbf{(1)}$$

where C, CH, CH_2 and CH_3 are the number of calculated unique (binned with 0.5 ppm resolution) ^{13}C resonances corresponding to carbons with 0, 1, 2 and 3 attached hydrogens, respectively. The distribution of MA *vs.* NMR-predicted MA is visualised as a histogram is shown in **Fig. S8**.

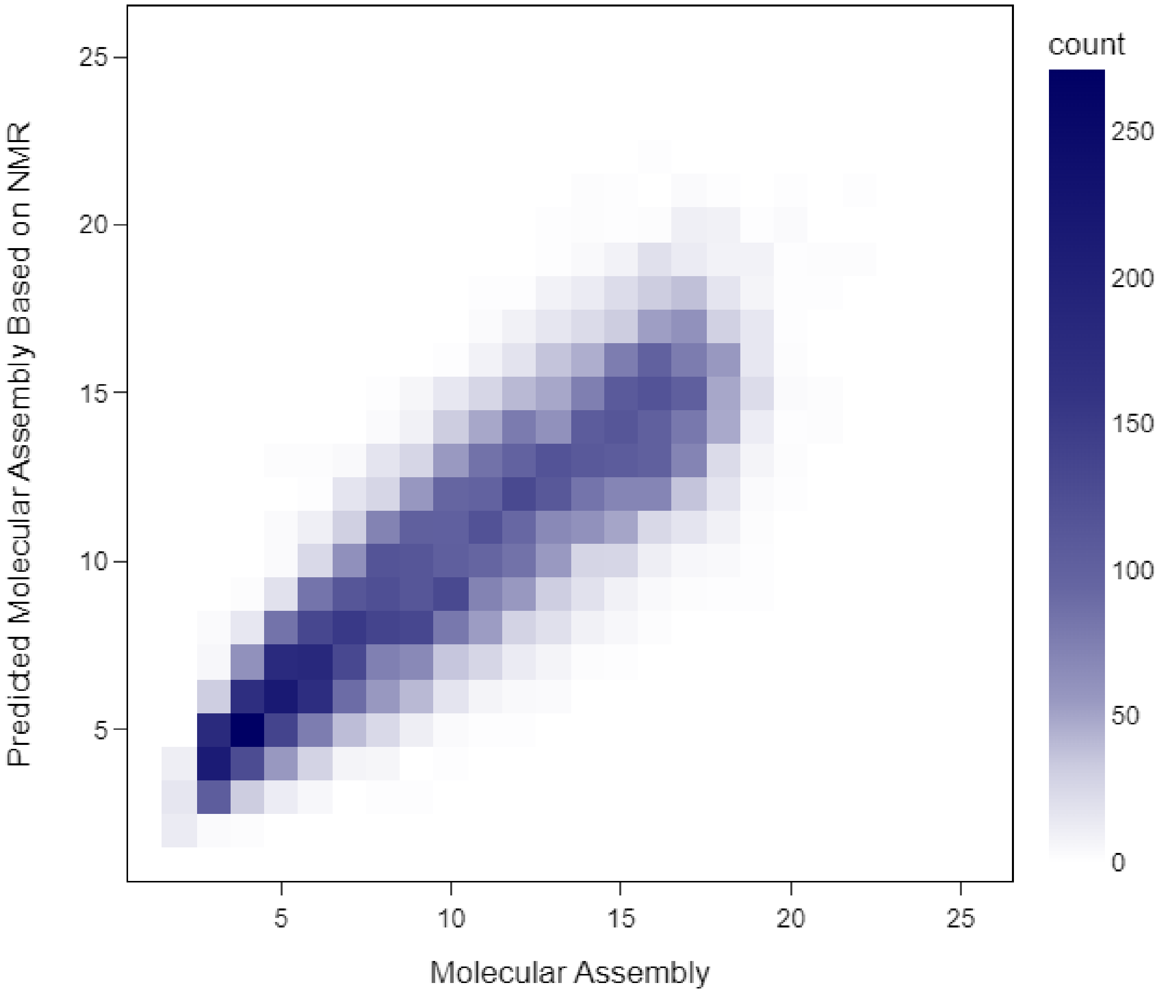

Fig. S8. Histogram of predicted MA (based on the **Eq. 1**) *vs.* MA on 10,000 compounds sample.

2.3 Infrared Spectroscopy – xTB simulations

To predict the IR spectra of the sampled molecules, we have used the xTB-service tool(9, 10) for faster prediction over a large dataset. The provided Python interface(11) was used with the default setting, using 100 seconds as timeout for the geometry optimisation using the GFNFF forcefield. The default gaussian broadening was not applied to the observed intensity. Using the default threshold checks, calculated spectra assumed for the interpretation were based on the molecule with no large imaginary frequency (set as maximum $i \cdot 10$ cm^{-1}, although many structures possess small imaginary frequencies). The peaks in the range of 400–1500 cm^{-1} were counted with a threshold of 0.0005 (D/Å)^2 · amu^{-1} to not consider signals with 0 oscillatory strength and binned together peaks within 2 cm^{-1}. The coefficients for the simple linear function of the number of IR peaks were fit using the *statsmodels.api.OLS* module in python (**Fig. S9**).(8)

```
                     Results: Ordinary least squares
====================================================================
Model:              OLS              Adj. R-squared:     0.739
Dependent Variable: y                AIC:                45000.1544
Date:               2023-02-07 15:54 BIC:                45014.5751
No. Observations:   10000            Log-Likelihood:     -22498.
Df Model:           1                F-statistic:        2.826e+04
Df Residuals:       9998             Prob (F-statistic): 0.00
R-squared:          0.739            Scale:              5.2695
----------------------------------------------------------------------
            Coef.    Std.Err.     t        P>|t|     [0.025    0.975]
----------------------------------------------------------------------
x1          0.2076     0.0012   168.0982   0.0000    0.2052    0.2100
const      -0.1454     0.0654    -2.2246   0.0261   -0.2735   -0.0173
--------------------------------------------------------------------
Omnibus:              38.032        Durbin-Watson:           1.439
Prob(Omnibus):        0.000         Jarque-Bera (JB):        47.476
Skew:                 0.066         Prob(JB):                0.000
Kurtosis:             3.311         Condition No.:           151
====================================================================
```

Fig. S9. Print output from the fit of MA = $x_1 \times n_{peaks}$ + *const.*; using *statsmodels.api.OLS* in python.(8)

The best prediction of MA based on the xTB-based IR predicted data is thus:

$$\mathrm{MA} = 0.21 \times n_{\mathrm{IR\_peaks}} - 0.15 \qquad \textbf{(2)}$$

where $n_{\mathrm{IR\_peaks}}$ is the number of IR peaks in the region of 400–1500 cm^{-1} with intensity above 0.0005 $(D/Å)^2 \cdot amu^{-1}$. The distribution of MA *vs*. IR-predicted MA is visualised as a histogram in **Fig. S10**.

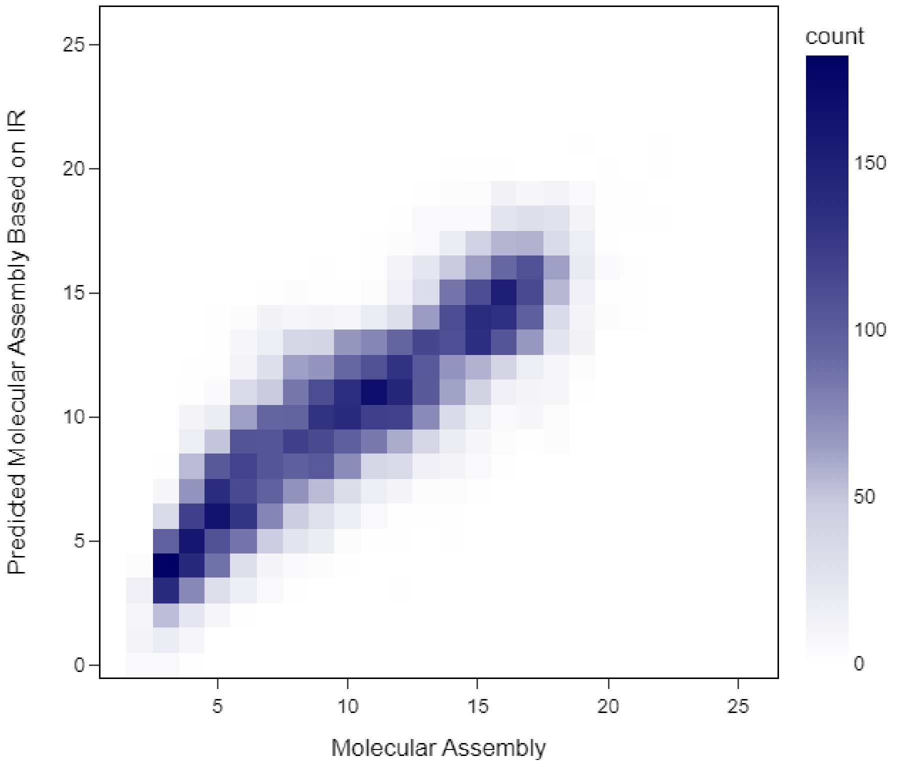

Fig. S10. Histogram of predicted MA (based on **Eq. 2**) *vs.* MA on 10,000 compounds dataset.

Our general hypothesis is that modes in the IR fingerprint region could be associated largely with collective motions, involving bonds from the various subgraphs of the whole structure. Therefore, from the number of the total modes in the fingerprint region, the overall molecular complexity could be inferred. To illustrate that on a simple and a complex molecule, vibrational modes in the fingerprint region (400–1500 cm^{-1}) above the set intensity threshold of 0.0005 $(D/Å)^2 \cdot amu^{-1}$ for chemical structures of 5-aminoisopthalic acid (**Fig. S11**.) and quinine (

Fig. S12–**Fig. S15**) are visualised. On the molecular structure, bonds involved in the vibrational modes are highlighted.

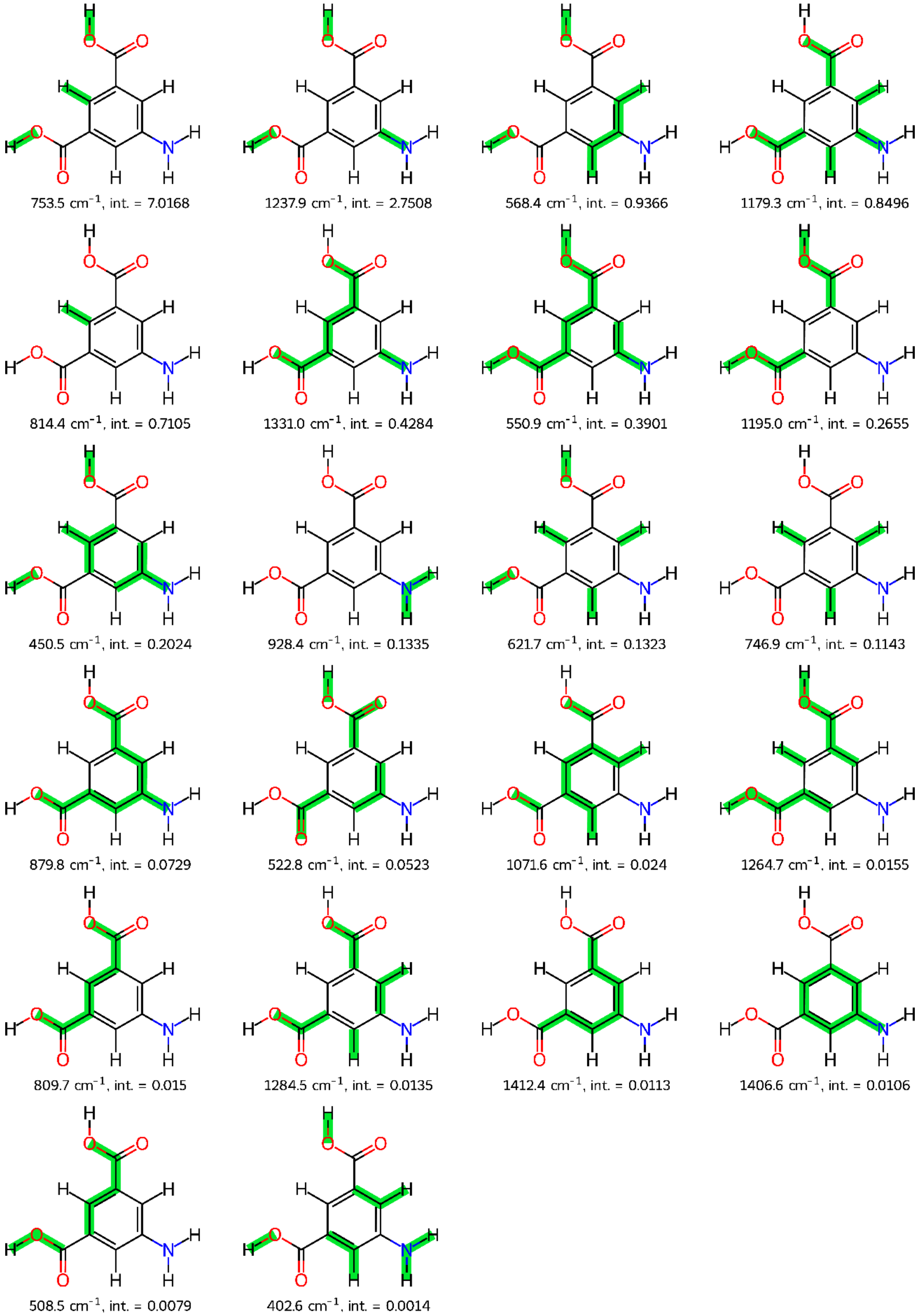

Fig. S11. Example of all vibrational bands of 5-aminoisopthalic acid in the fingerprint region demonstrating its collective-motion nature. Vibrational modes are ordered by intensity as calculated by xTB software.

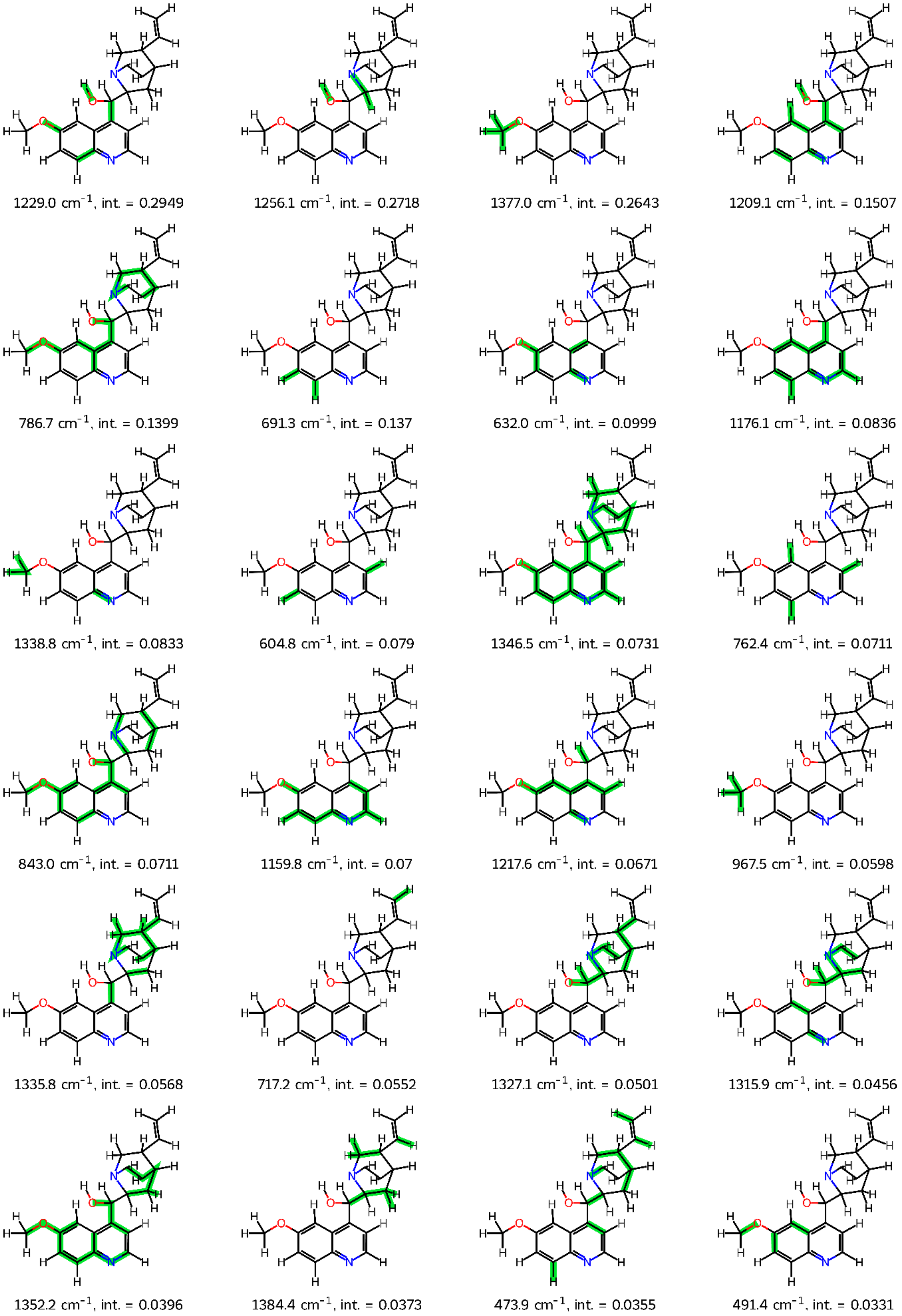

Fig. S12. Example of all vibrational bands of quinine in the fingerprint region demonstrating its collective-motion nature. Vibrational modes are ordered by intensity as calculated by xTB. (part 1)

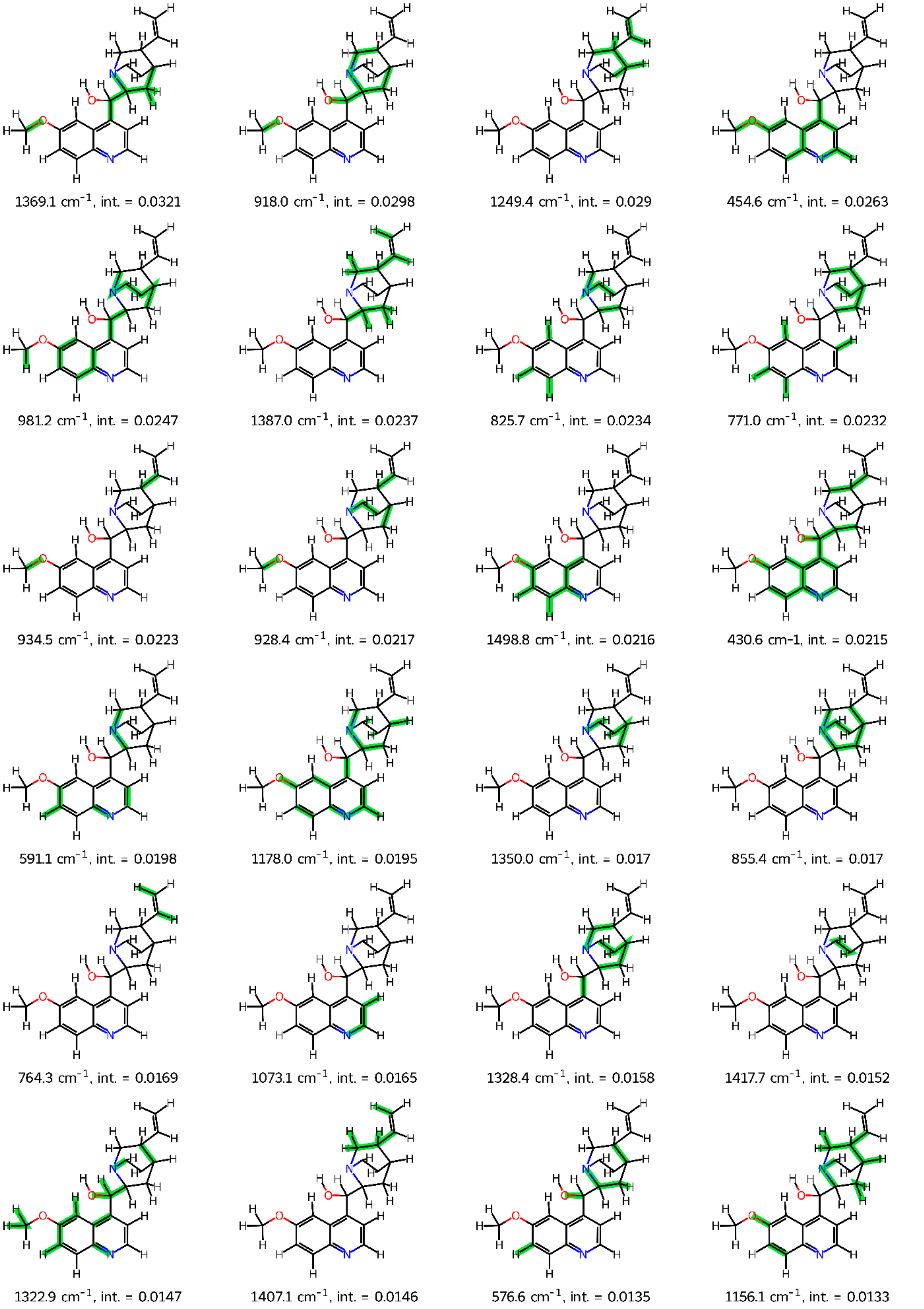

Fig. S13. Example of all vibrational bands of quinine in the fingerprint region demonstrating its collective-motion nature. Vibrational modes are ordered by intensity as calculated by xTB. (part 2)

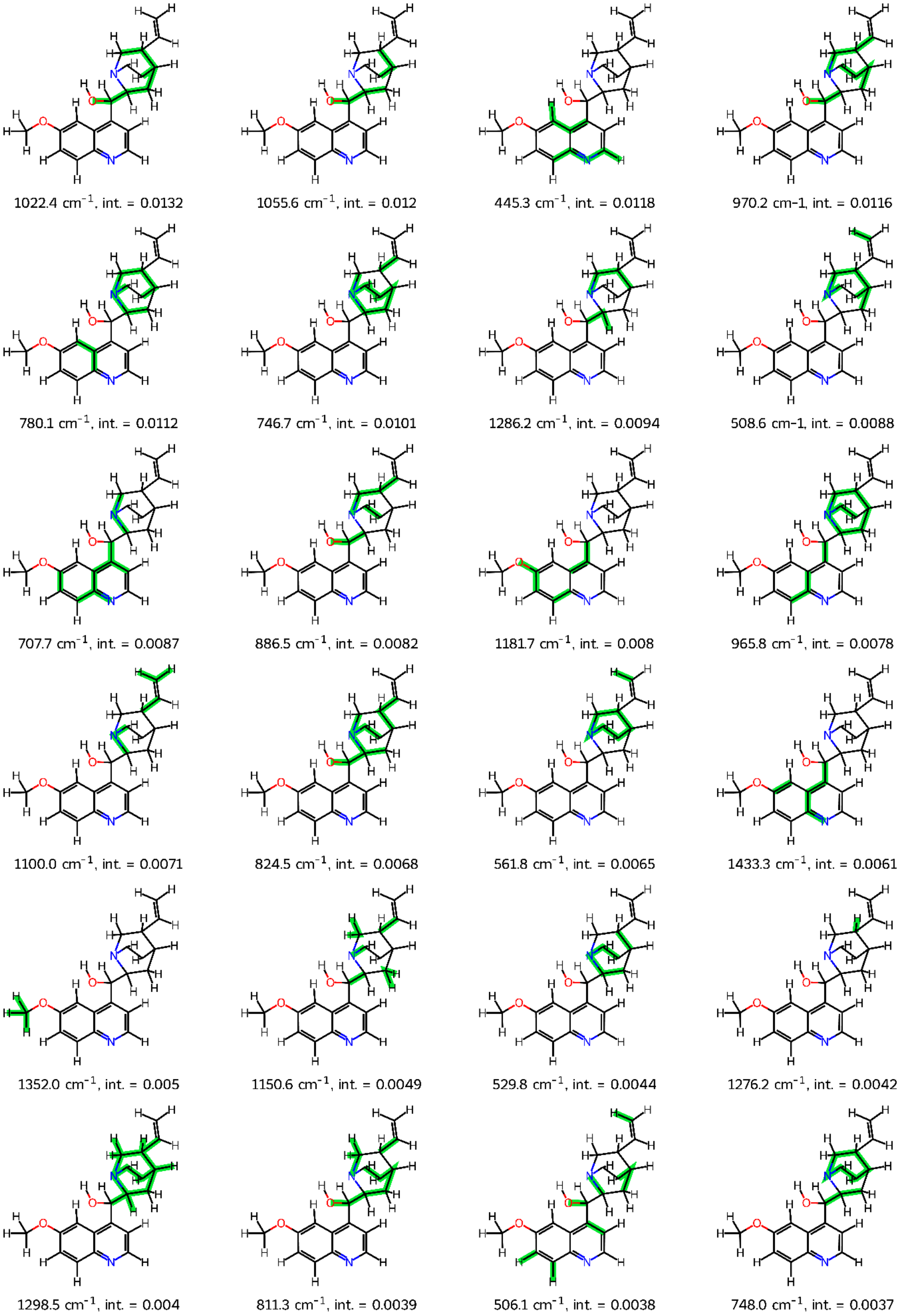

Fig. S14. Example of all vibrational bands of quinine in the fingerprint region demonstrating its collective-motion nature. Vibrational modes are ordered by intensity as calculated by xTB. (part 3)

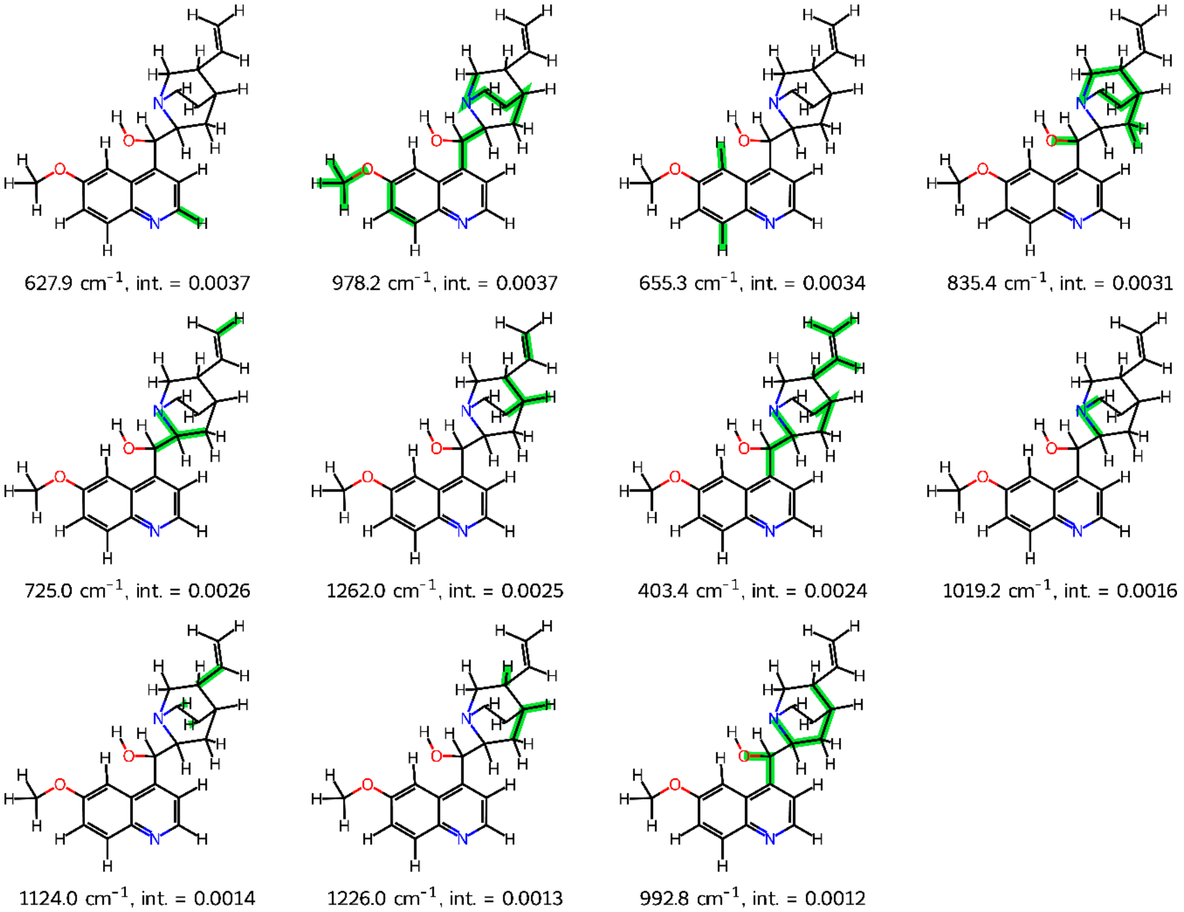

Fig. S15. Example of all vibrational bands of quinine in the fingerprint region demonstrating its collective-motion nature. Vibrational modes are ordered by intensity as calculated by xTB. (part 4)

2.4 Infrared Spectroscopy – DFT simulations

To validate potential inaccuracies in the predicted frequency spectra using the semiempirical method, a detailed rigorous analysis over a limited set of 101 compounds was performed. The theoretical approach has been used as described in the work by Swart and colleagues.(12) Detailed quantum chemical simulations were performed using Amsterdam Density Functional (ADF 2017)(13) software. In this study, QUILD(14) (Quantum-regions interconnected by Local descriptions) program was used with delocalized coordinates for the optimization of equilibrium structures until the maximum gradient component was less than 10^{-4} a.u. Energies, gradients and Hessians for vibrational frequencies including Raman intensities were calculated using BP86-D3(15–17) with a triple/double-zeta valence plus polarization basis set (TZP for metals, DZP for other elements). In all cases, these calculations included solvation effects through the COSMO(18) dielectric continuum model with appropriate parameters for solvent, and scalar relativistic corrections through Zeroth Order Regular Approximation (ZORA).[11]

The number of peaks in the fingerprint region (400–1500 cm^{-1}) above an intensity threshold (25 km·mol^{-1}) was found to be 0.76 (**Fig. S16**). The histogram of calculated MA *vs.* the expected is depicted in **Fig. S17**.

```
                 Results: Ordinary least squares
=================================================================
Model:              OLS              Adj. R-squared:     0.570
Dependent Variable: y                AIC:                627.4633
Date:               2023-02-08 15:07 BIC:                632.9003
No. Observations:   112              Log-Likelihood:     -311.73
Df Model:           1                F-statistic:        148.3
Df Residuals:       110              Prob (F-statistic): 4.06e-22
R-squared:          0.574            Scale:              15.592
--------------------------------------------------------------------
         Coef.     Std.Err.       t       P>|t|     [0.025    0.975]
--------------------------------------------------------------------
x1       0.4859      0.0399    12.1773    0.0000    0.4068    0.5650
const    5.6173      0.8135     6.9053    0.0000    4.0051    7.2294
-----------------------------------------------------------------
Omnibus:              4.323       Durbin-Watson:          1.179
Prob(Omnibus):        0.115       Jarque-Bera (JB):       4.310
Skew:                 0.474       Prob(JB):               0.116
Kurtosis:             2.842       Condition No.:          45
=================================================================
```

Fig. S16. Print output from the fit of MA = $x_1 \times n_{\mathrm{IR\_peaks}}$ + *const.*; using *statsmodels.api.OLS* in Python.(8)

The list of chemical structures of all compounds used for the DFT study is in **Fig. S18**–**Fig. S20**.

The best model for inferring the MA based on the DFT-predicted IR spectra is thus:

$$\mathrm{MA} = 0.49 \times n_{\mathrm{IR\_peaks}} + 5.6 \qquad \textbf{(3)}$$

where $n_{\mathrm{IR\_peaks}}$ is the number of IR peaks in the region of 400–1500 cm^{-1} with intensity above 25 km·mol^{-1}.

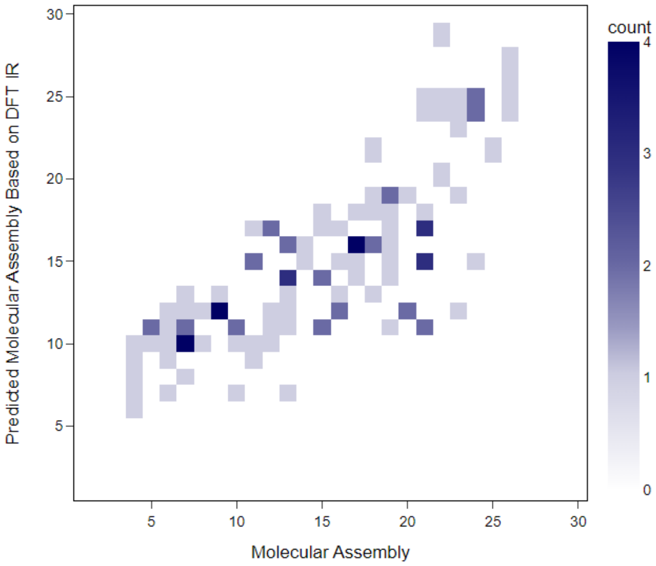

Fig. S17. Histogram of predicted MA (based on the **Eq. 3**) *vs.* MA on 112 compounds dataset of DFT calculated IR peaks in the range of 400–1500 cm^{-1} above intensity 25 $km{\cdot}mol^{-1}$.

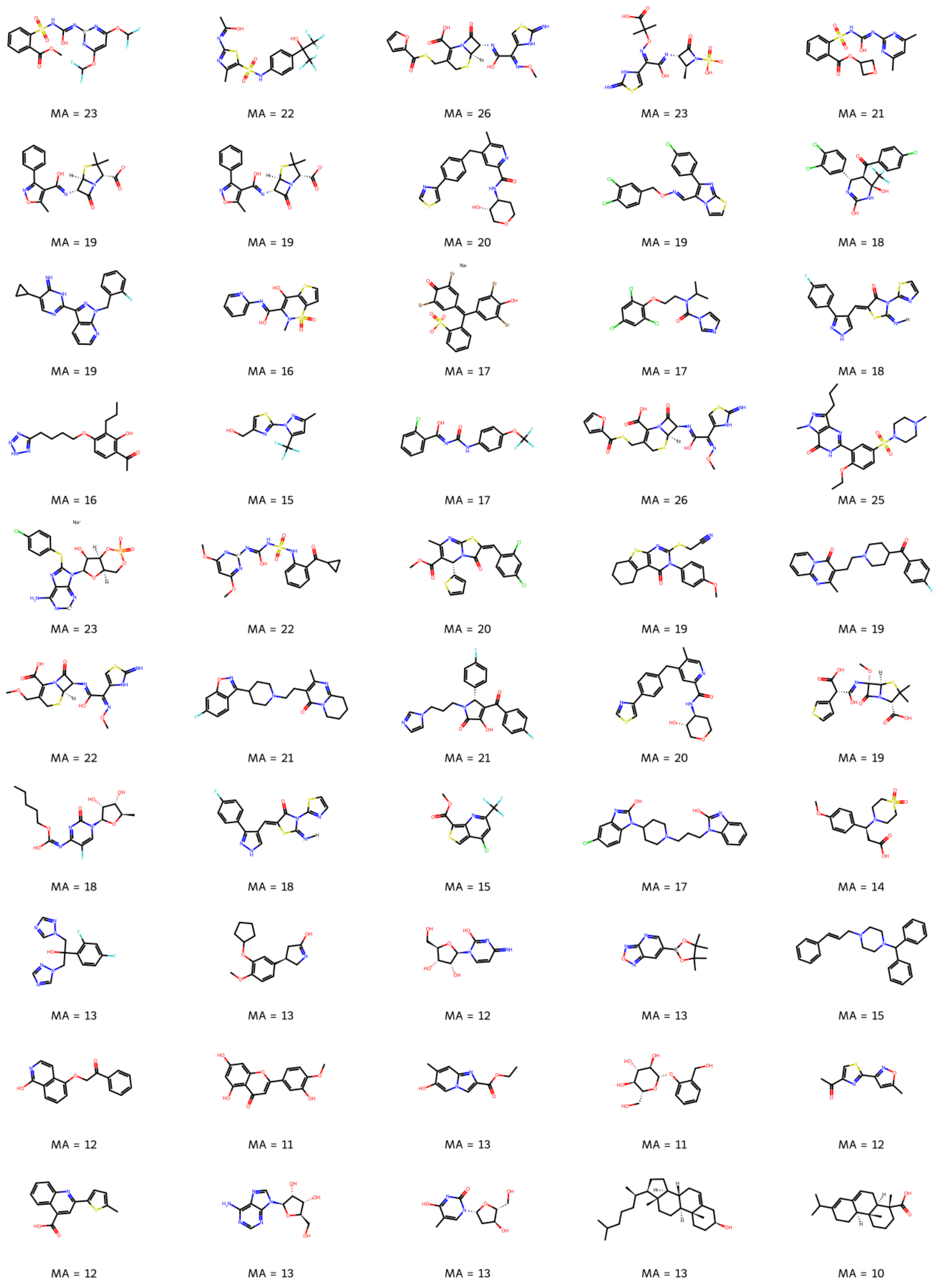

Fig. S18. Molecular structures with calculated molecular assembly (MA) were used in the DFT-calculated IR study (**Part 1**).

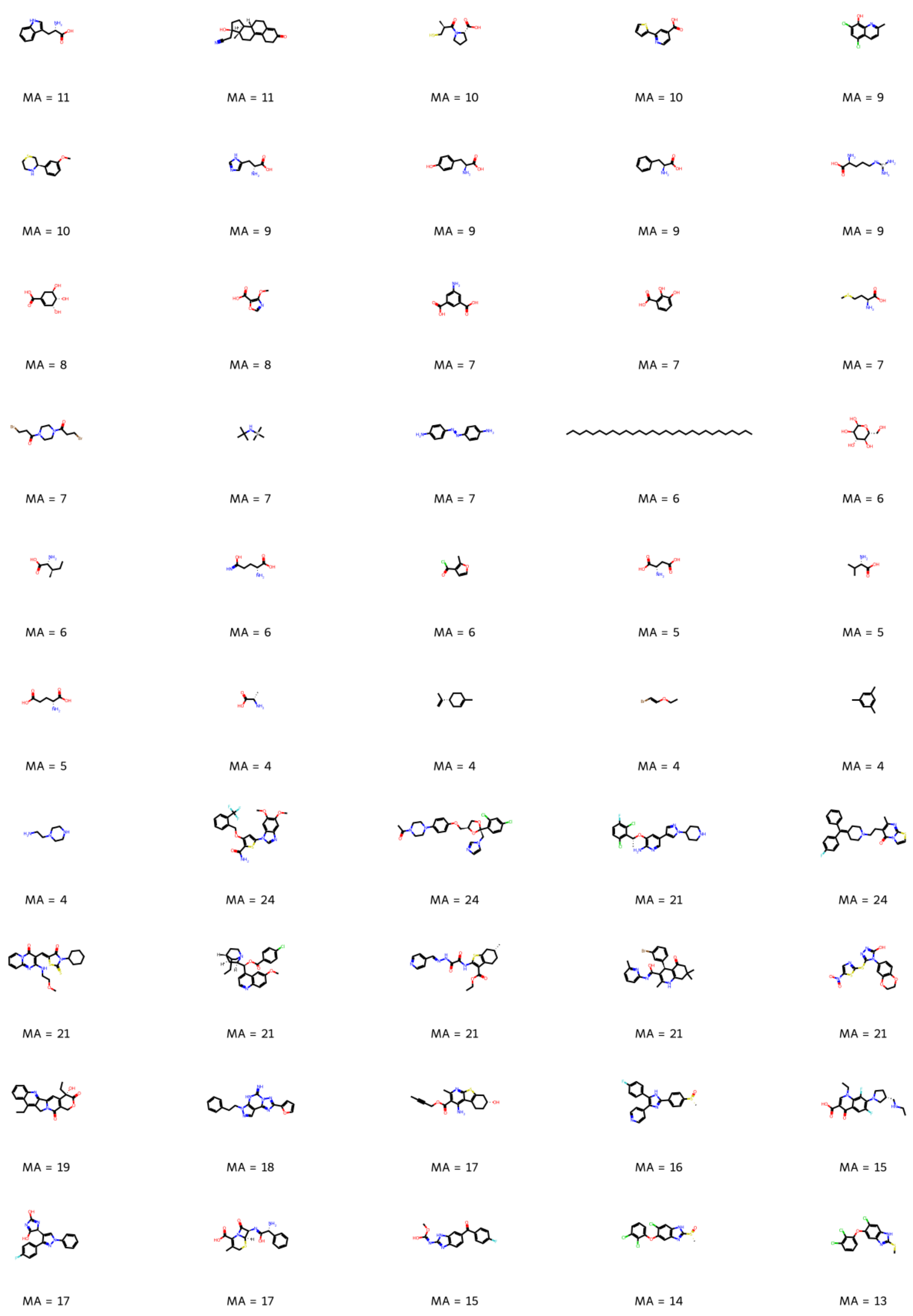

Fig. S19. Structures with calculated molecular assembly (MA) used in the DFT calculated IR study (**Part 2**)

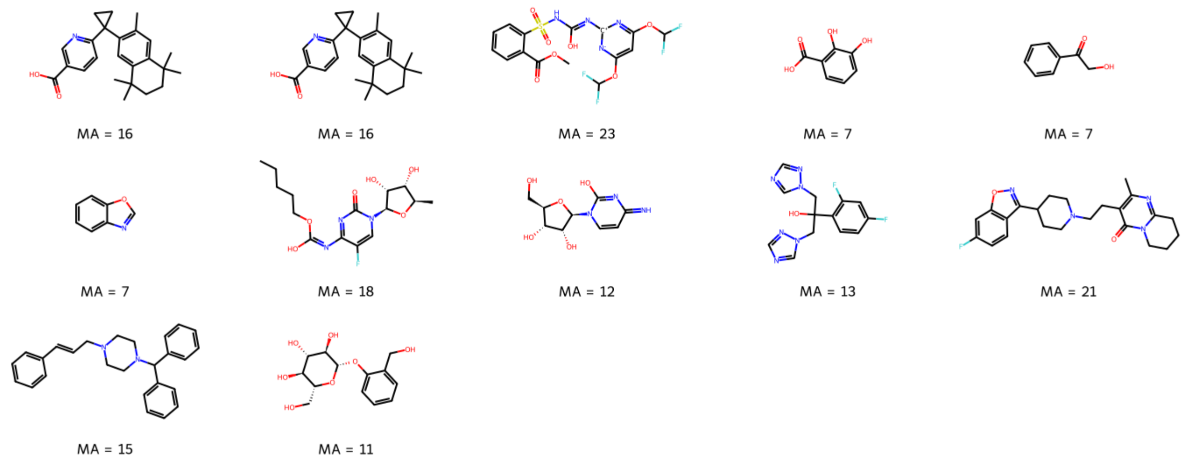

Fig. S20. Structures with calculated molecular assembly (MA) used in the DFT calculated IR study (**Part 3**)

3 Experimental Infrared Spectroscopy

All experimental IR spectra were acquired on a Thermo Scientific Nicolet iS5 with Specac Golden Gate Reflection Diamond ATR System. All samples were measured in their native state at room temperature (solid state unless liquid at room temperature). The acquired data were processed with Thermo Scientific OMNIC 8.3.103 software; using diamond attenuated total reflectance IR (64 scans, resolution 2 cm^{-1}). The spectra were processed at 50% sensitivity and 80% threshold for selecting peaks using OMNIC software (see an example of an acquired spectrum in **Fig. S21**). IR peaks in the fingerprint region (400-1500 cm^{-1}) were counted and correlated against the MA of the molecule. To reduce the error between sample screenings, the background IR spectra were recorded after every 3rd sample measurement. Linear regression fit between the experimental IR peaks number in the fingerprint region *vs.* MA agreed provided simple model (**Eq. 4**) with a Pearson's correlation coefficient 0.75:

$$\mathrm{MA} = 0.45 \times n_{\mathrm{IR\_peaks}} + 2.26 \qquad \textbf{(4)}$$

where $n_{\mathrm{IR\_peaks}}$ is the number of IR peaks in the region of 400–1500 cm^{-1}. The distribution of MA *vs.* IR-predicted MA is visualised as a histogram in **Fig. S22**. Structures of all compounds used in the study are shown in **Fig. S23** and **Fig. S24**.

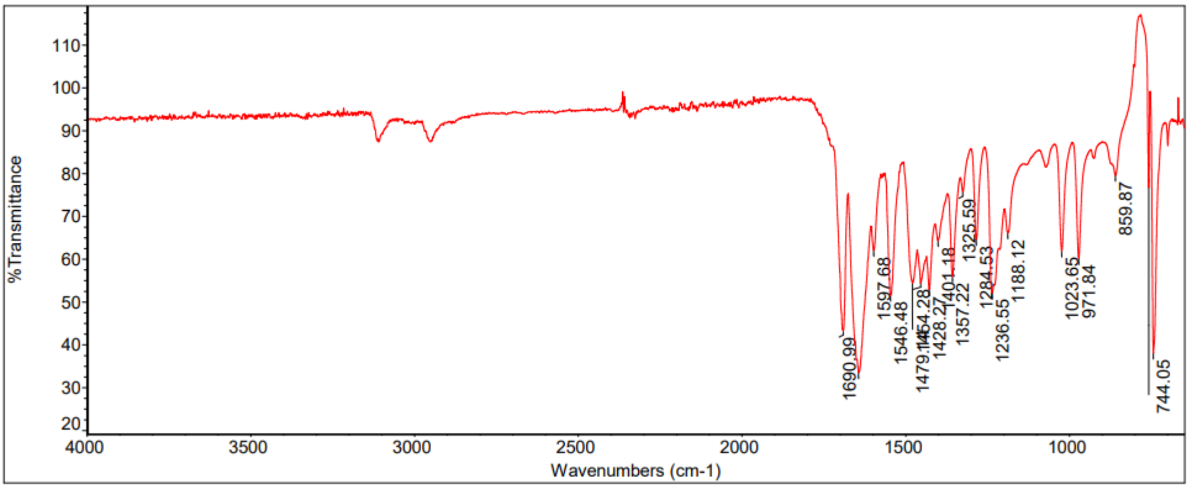

Fig. S21. Example of an experimental IR spectrum of Caffeine. Identified peaks in the fingerprint region 400–1500 cm^{-1} considered in the peak count.

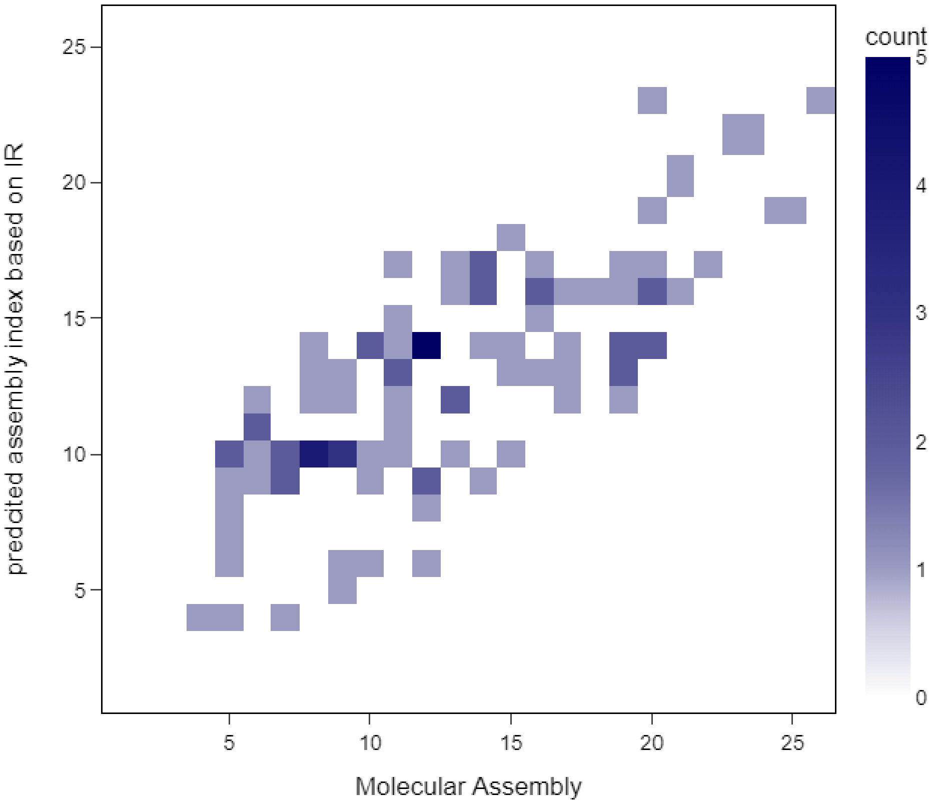

Fig. S22. Histogram of predicted MA based on the 99 experimental IR data using **Eq. 4** *vs.* MA.

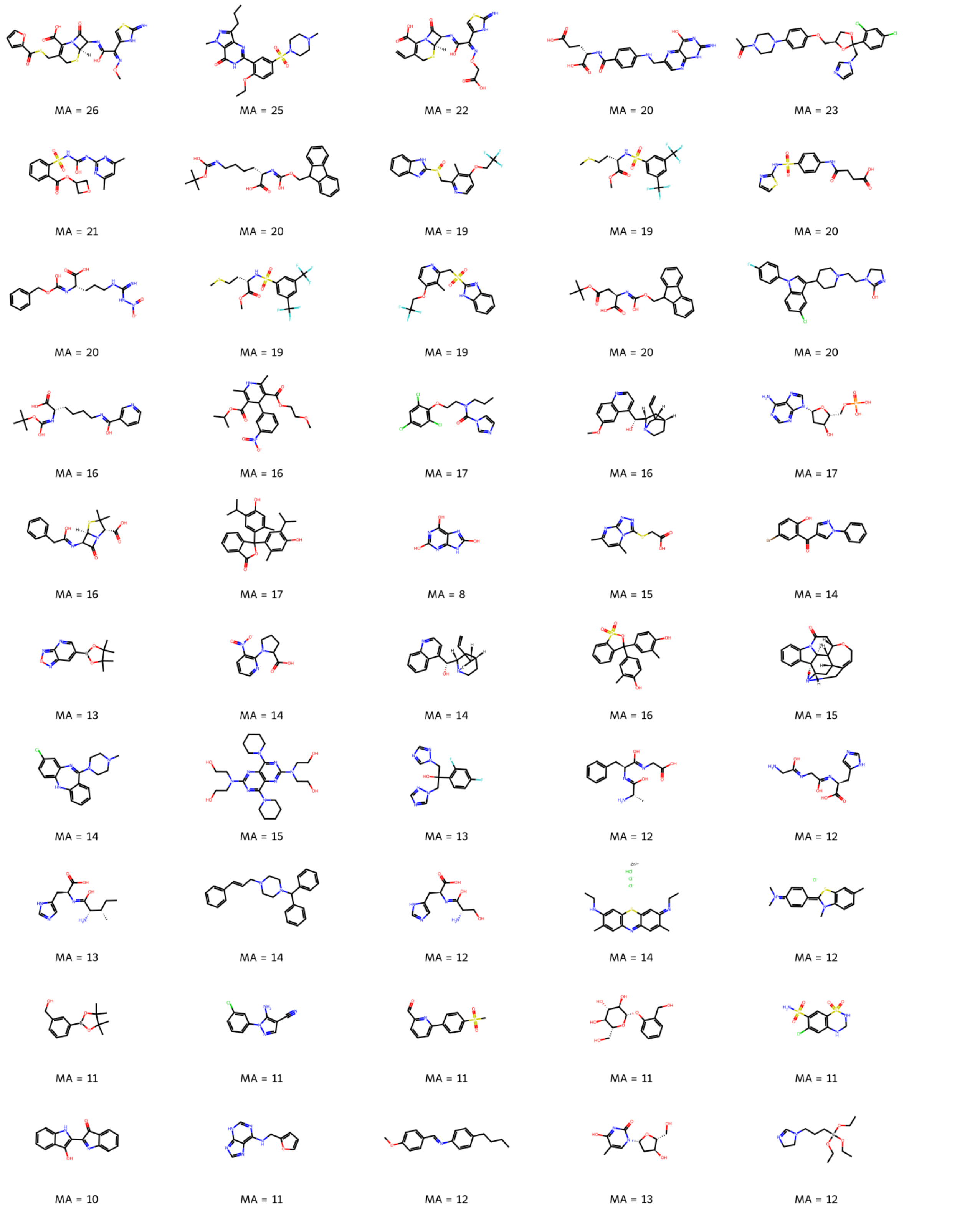

Fig. S23. Structures with calculated molecular assembly (MA) used in the experimental IR study (Part 1).

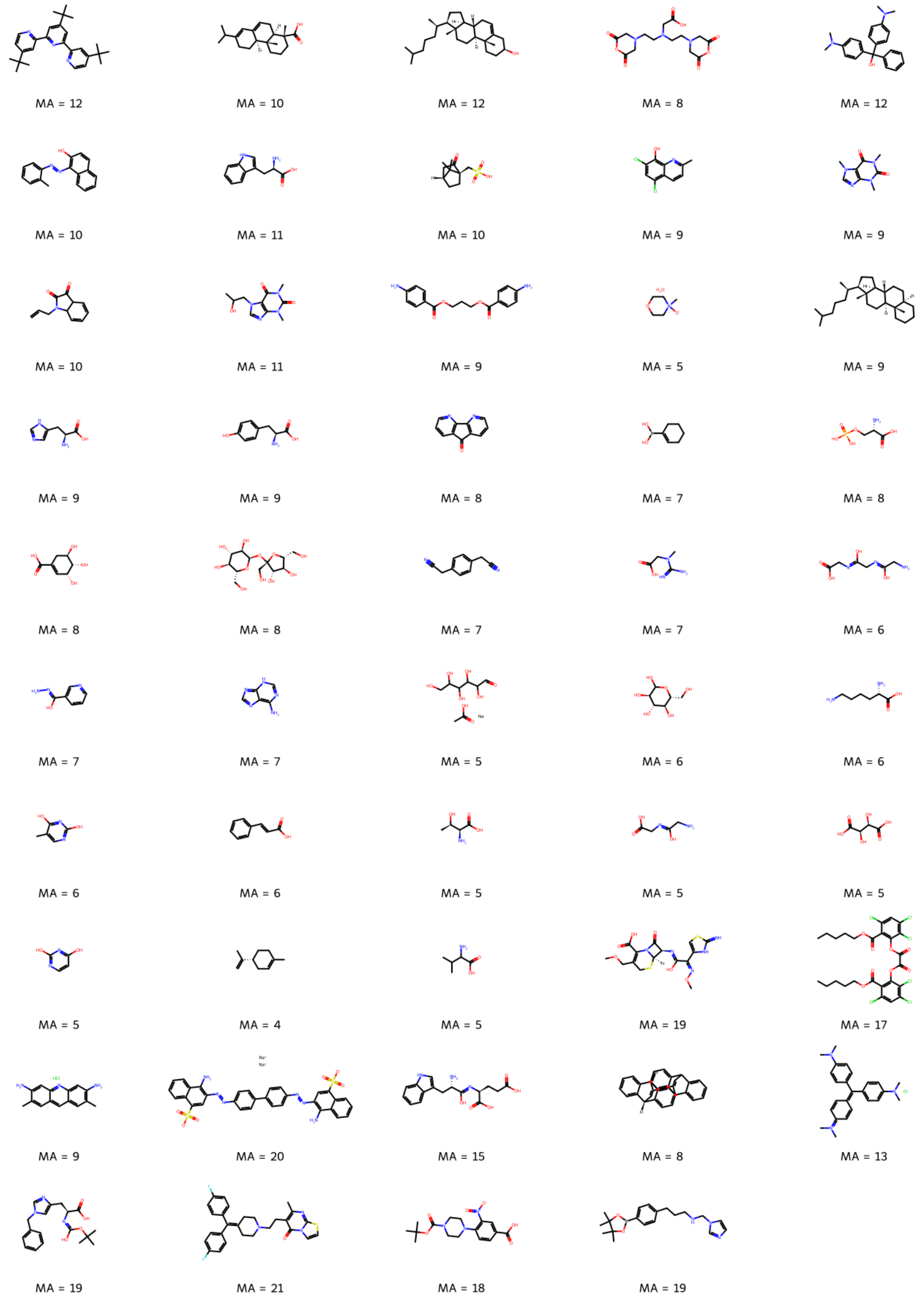

Fig. S24. Structures with calculated molecular assembly (MA) used in the experimental IR study (Part 2).

The coefficients for the simple linear function of the number of IR peaks were fitted using the *statsmodels.api.OLS* module in Python was used (**Fig. S25**).(8)

```
                  Results: Ordinary least squares
==================================================================
Model:              OLS              Adj. R-squared:     0.563
Dependent Variable: y                AIC:                528.8456
Date:               2023-02-07 17:18 BIC:                534.0358
No. Observations:   99               Log-Likelihood:     -262.42
Df Model:           1                F-statistic:        127.3
Df Residuals:       97               Prob (F-statistic): 2.33e-19
R-squared:          0.568            Scale:              11.989
---------------------------------------------------------------------
         Coef.     Std.Err.       t       P>|t|     [0.025    0.975]
---------------------------------------------------------------------
x1       0.4531      0.0402    11.2845    0.0000    0.3734    0.5328
const    2.2642      0.9862     2.2960    0.0238    0.3070    4.2215
------------------------------------------------------------------
Omnibus:               21.605       Durbin-Watson:          1.322
Prob(Omnibus):         0.000        Jarque-Bera (JB):       5.620
Skew:                  0.200        Prob(JB):               0.060
Kurtosis:              1.903        Condition No.:          70
==================================================================
```

Fig. S25. Print output from the fit of MA = $x_1 \times n_{\text{peaks}}$ + *const*. for experimental IR spectra; using *statsmodels.api.OLS* in Python.(8)

4 Experimental NMR

The investigation of NMR spectroscopy as a prediction tool for MA was experimentally examined on 101 molecules with a range of MA 3–26. Here, the same model that was used in the prediction of MA from the number of different ^{13}C resonances in the theoretical dataset was also used to predict MA from was used as developed in the theoretical NMR set (**Eq. 1**).

4.1 Sample Preparation details

All samples were prepared with 600 mL d_6-DMSO in a 5 mm Bruker 600 MHz rated NMR tube. Concentrations varied from 0.19 mM to 1.17 M due to solubility factors. Several samples (5-aminioisophthalic acid, Oxacillin Sodium Salt, Sildenafil, Triclabendazole) were analyzed *via* NMR at 5, 30 and 300 mM and have shown a minimal effect of the concentration on the extracted number of chemical environment values from spectra. Samples that did not dissolve in d_6-DMSO were dissolved in a D_2O:d_3-MeCN mixture (ratio 75:25).

4.2 NMR Experimental Parameters

The NMR used was a Bruker Ascend Aeon 600 MHz NMR spectrometer with a CP DCH 600S3 C/H-D-05 Z cryoprobe installed. All data was processed using Bruker Topspin 3.6.2 and Mestrenova 14.1.1-2451. The NMR experimental parameters are as follows: ^1H NMR(16 scans, 20 ppm spectral

width, 3.46 second acquisition time, 2.00 second relaxation delay), ^{13}C NMR(128 scans, 250 ppm spectral width, 1.73 second acquisition time, 0.80 second relaxation delay), ^{13}C DEPTQ 90 and DEPTQ 135 (64 scans,250 ppm spectral width,1.73 seconds acquisition time, 1.00 second relaxation delay), ^{1}H PSYCHE(16 scans, 12.49 ppm spectral width, 0.89 seconds acquisition time, 1.00 second relaxation delay), HSQC (8 scans, 10 ppm spectral width F2, 250 spectral width F1, 0.09 second acquisition time, 1.49 seconds relaxation delay), ^{13}C DOSY (pseudo-2D experiment scans, 200 ppm spectral width F2, 8 TD points, 1.10 second acquisition time, 8.00 second relax delay, 0.80 second diffusion time d20, 1450 µsec gradient pulse P30).

The experiments were tested on several examples to cover a range of concentrations from 5 mM to 300 mM to prove the same number of individual carbon peak types can be achieved, regardless of the concentration.

4.3 Classification of the Carbon Types

Accurate counts for all individual ^{13}C type environments were obtained using a combination of ^{13}C, DEPTQ135, DEPTQ90 and HSQC analysis to ensure maximum accuracy before unblinding samples.

To determine the degree of substitution for ^{13}C chemical environments, this was first approached using two types of ^{13}C DEPT experiments, DEPTQ 135 and DEPTQ 90, that phase carbon signals positive or negative depending on their degree of substitution. The DEPTQ experiment was chosen (not the DEPT) to detect quaternary carbons (otherwise not detected by DEPT). The 135 and 90 portion stands for the final ^{1}H tip angle of the pulse in the pulse program before acquisition.(19, 20)

First, ^{13}C DEPTQ 135 observes quaternary and CH_2 peaks as one phase and the CH and CH_3 peaks as the other. The ^{13}C DEPTQ 90 then is measured to complement the ^{13}C DEPTQ 135 with only the detection of quaternary peaks in one phase and the CH peaks in the other. Using DEPTQ135 and DEPTQ 90 together, all degrees of substitution of the carbons can be identified via NMR. The solvent (expected as quaternary if deuterated) peak must be disregarded in the two counts of carbon peaks. To verify the assignment of the degree of substitution for ^{13}C chemical environments in blind samples, ^{1}H-^{13}C HSQC was used. In the HSQC experiment, the peaks are phased as they are in DEPT with CH_2 cross peaks in one phase and CH and CH_3 in the other. As CH_2 cross peaks are detected in their phase alone, this provides an easy method of counting the number of CH_2 chemical environments from CH_2 cross peaks. Quaternary ^{13}C cross peaks are not detected in HSQC.

The herein described workflow is illustrated on quinine, and its ^{13}C NMR (**Fig. S26**), DEPTQ-90 (**Fig. S27**), DEPTQ-135 (**Fig. S28**) and ^{1}H-^{13}C HSQC (**Fig. S29**) spectra. Structures of all compounds used in the NMR study are shown in **Fig. S30** and **Fig. S31**.

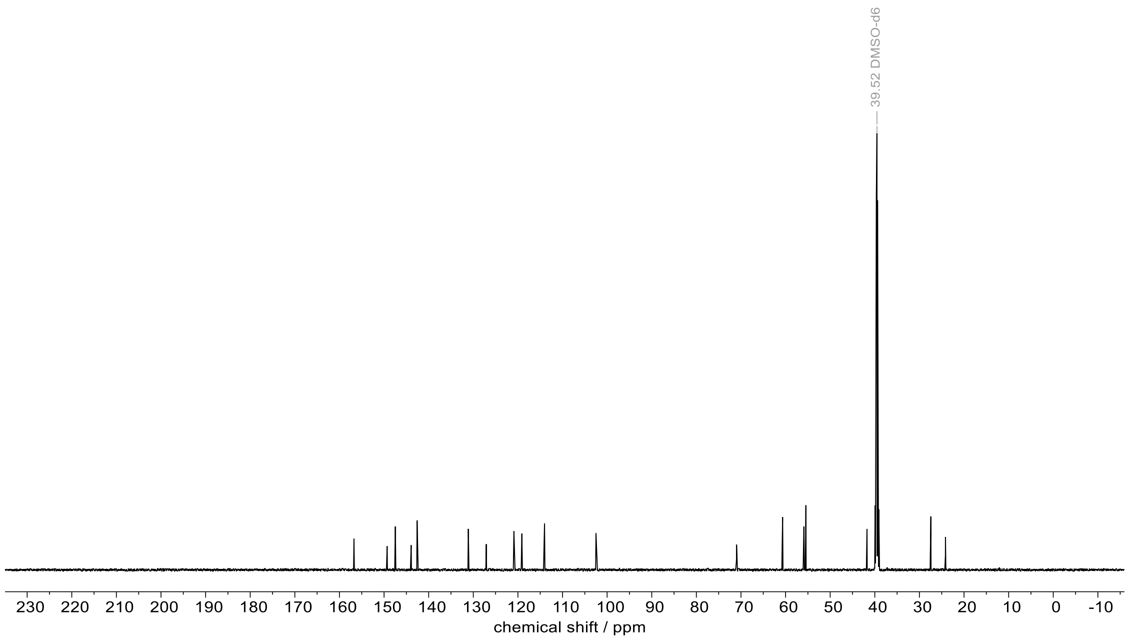

Fig. S26. ^{13}C NMR (150 MHz) of quinine.

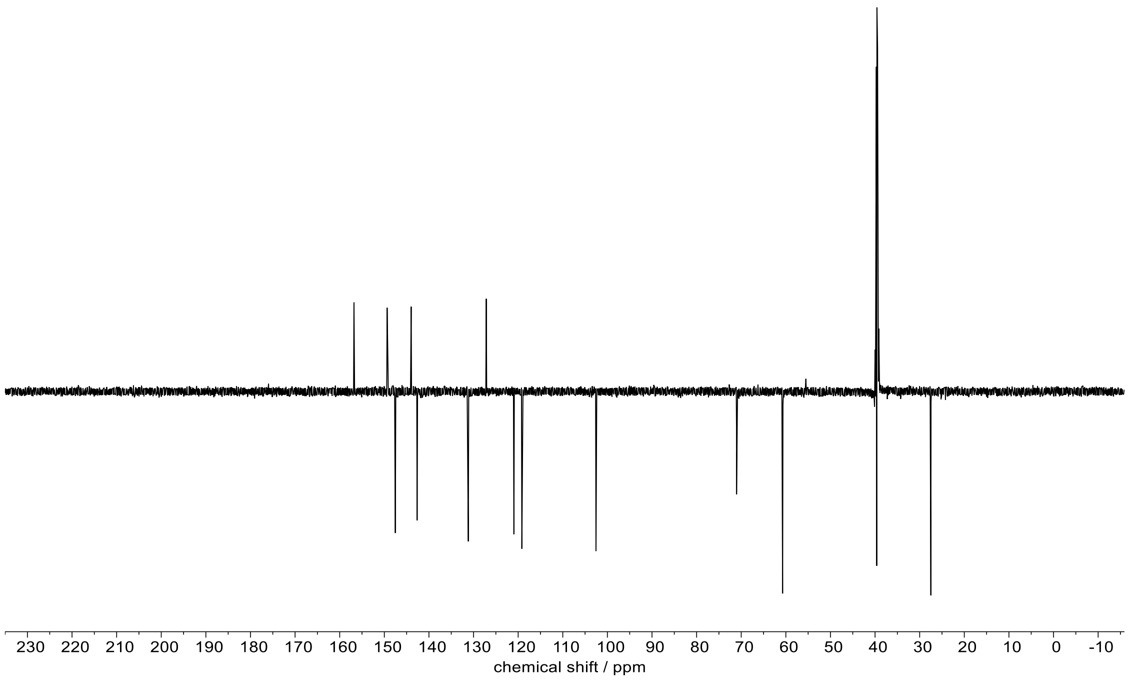

Fig. S27. DEPTQ-90 ^{13}C NMR (150 MHz) of quinine. Peaks at positive phase are C, peaks at negative are CH. The solvent peak needs to be subtracted from the C count (in this case DMSO-d6 in the positive phase).

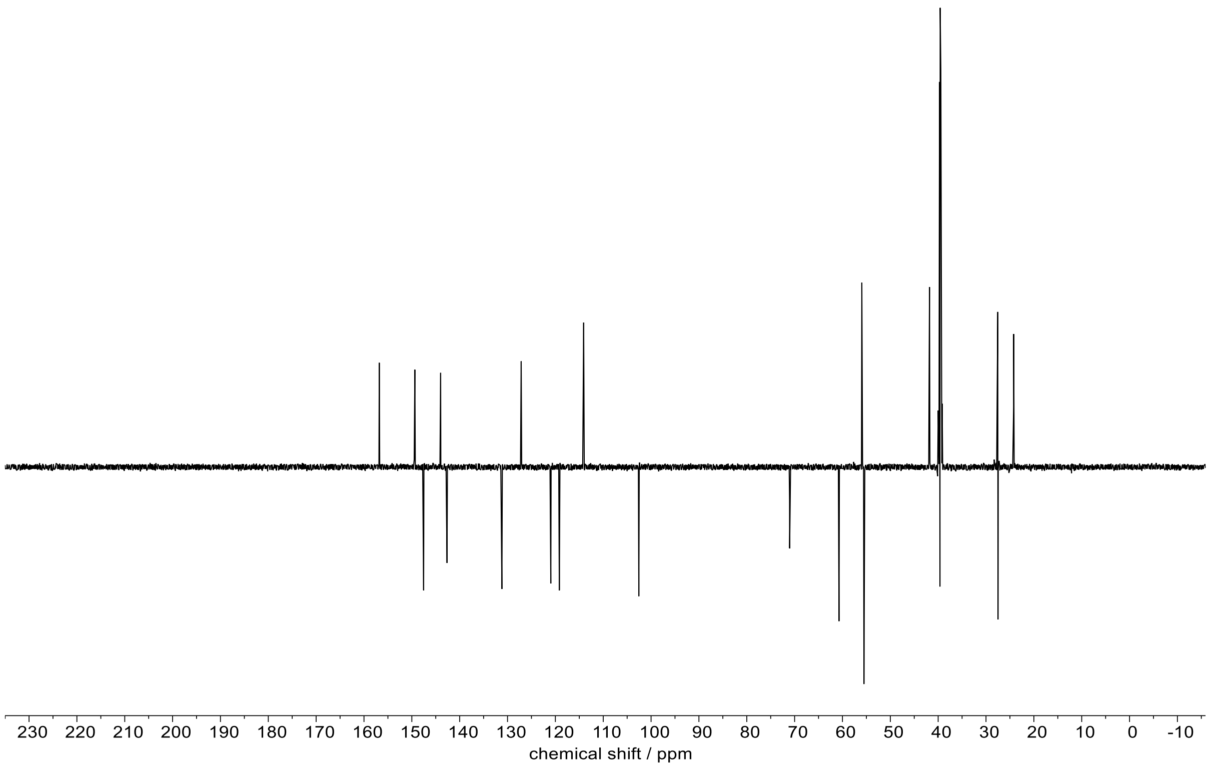

Fig. S28. DEPTQ-135 ^{13}C NMR (150 MHz) of quinine. Peaks at the positive phase are C and CH_2, and peaks at the negative as CH and CH_3.

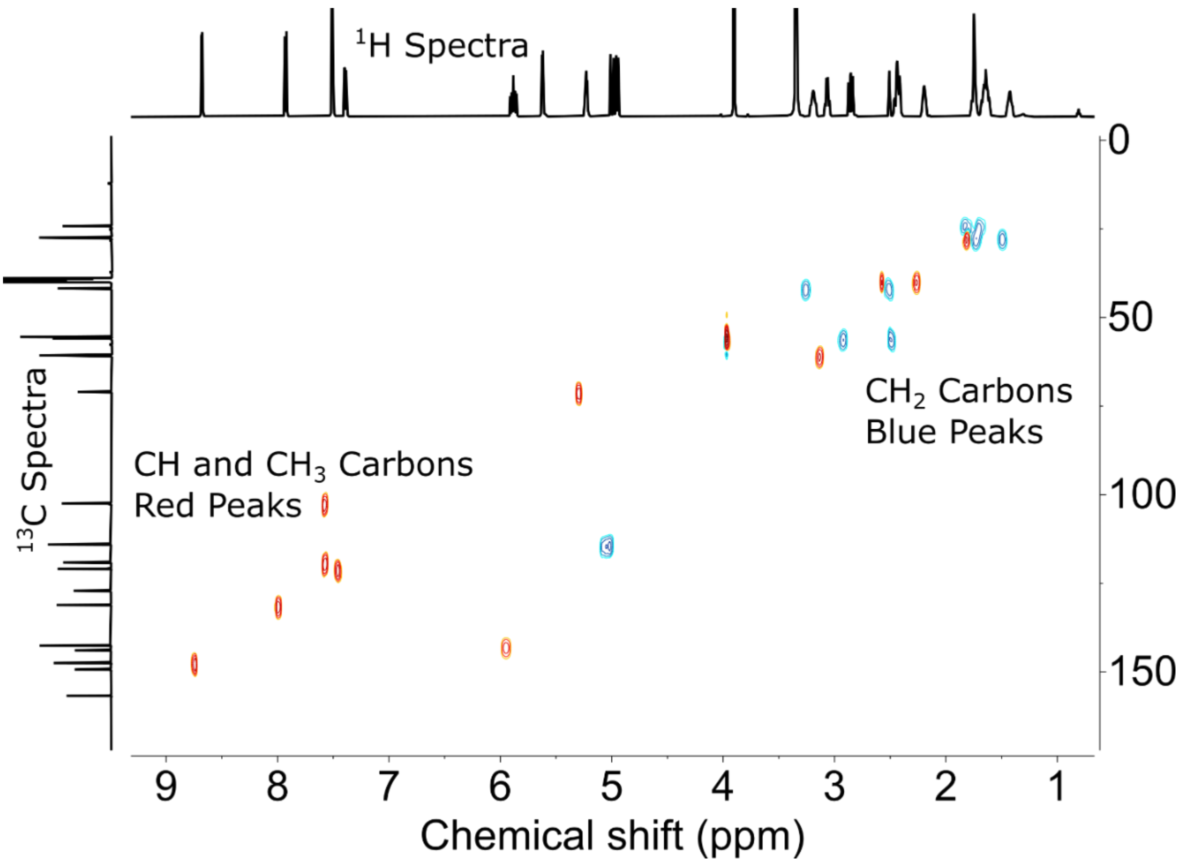

Fig. S29. ^{1}**H-**^{13}**C HSQC** Spectrum of quinine highlighting cross peaks of different phases (CH/CH_3 in red and CH_2 in blue).

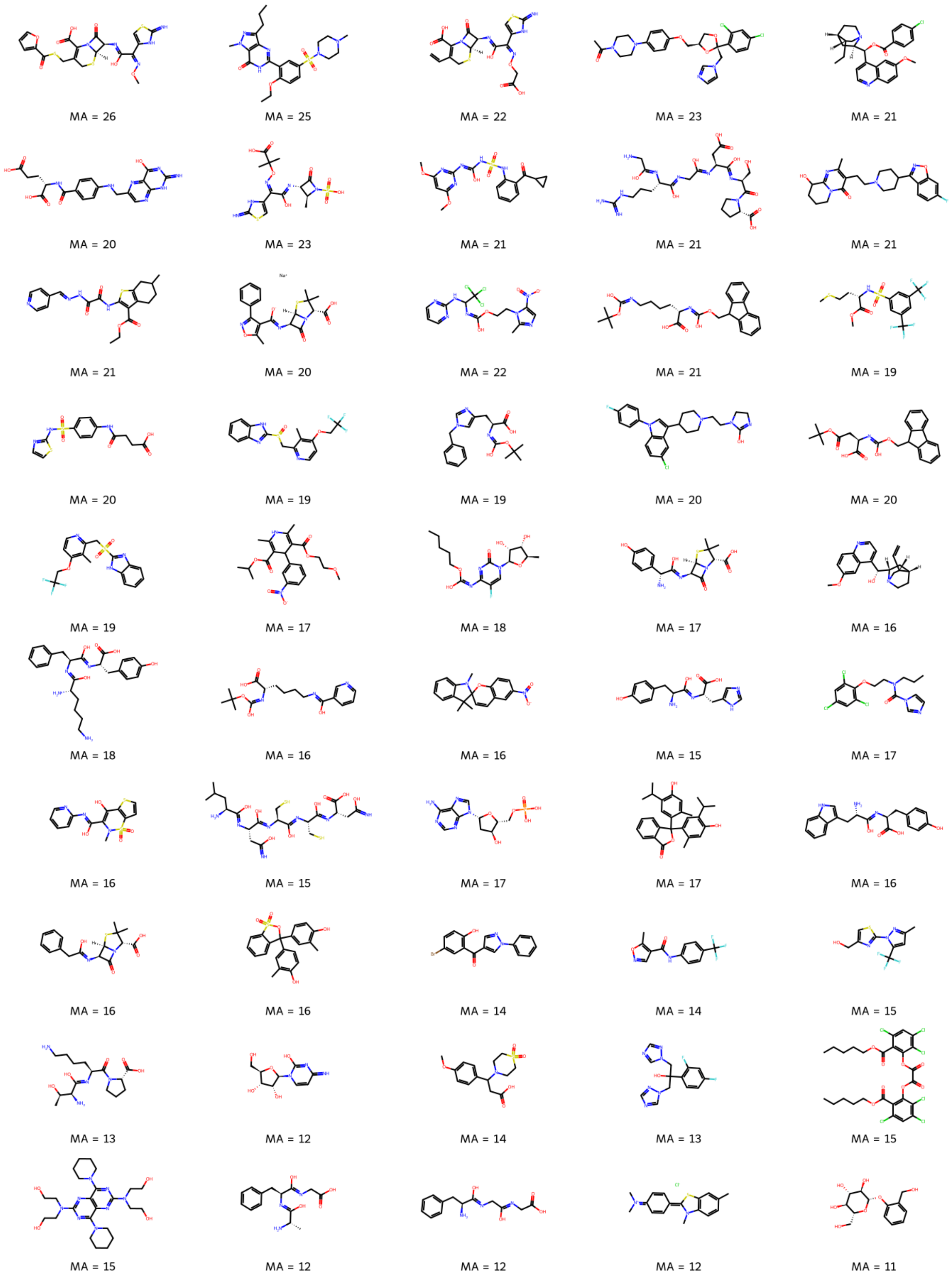

Fig. S30. Structures with calculated molecular assembly (MA) used in the experimental NMR study (part 1).

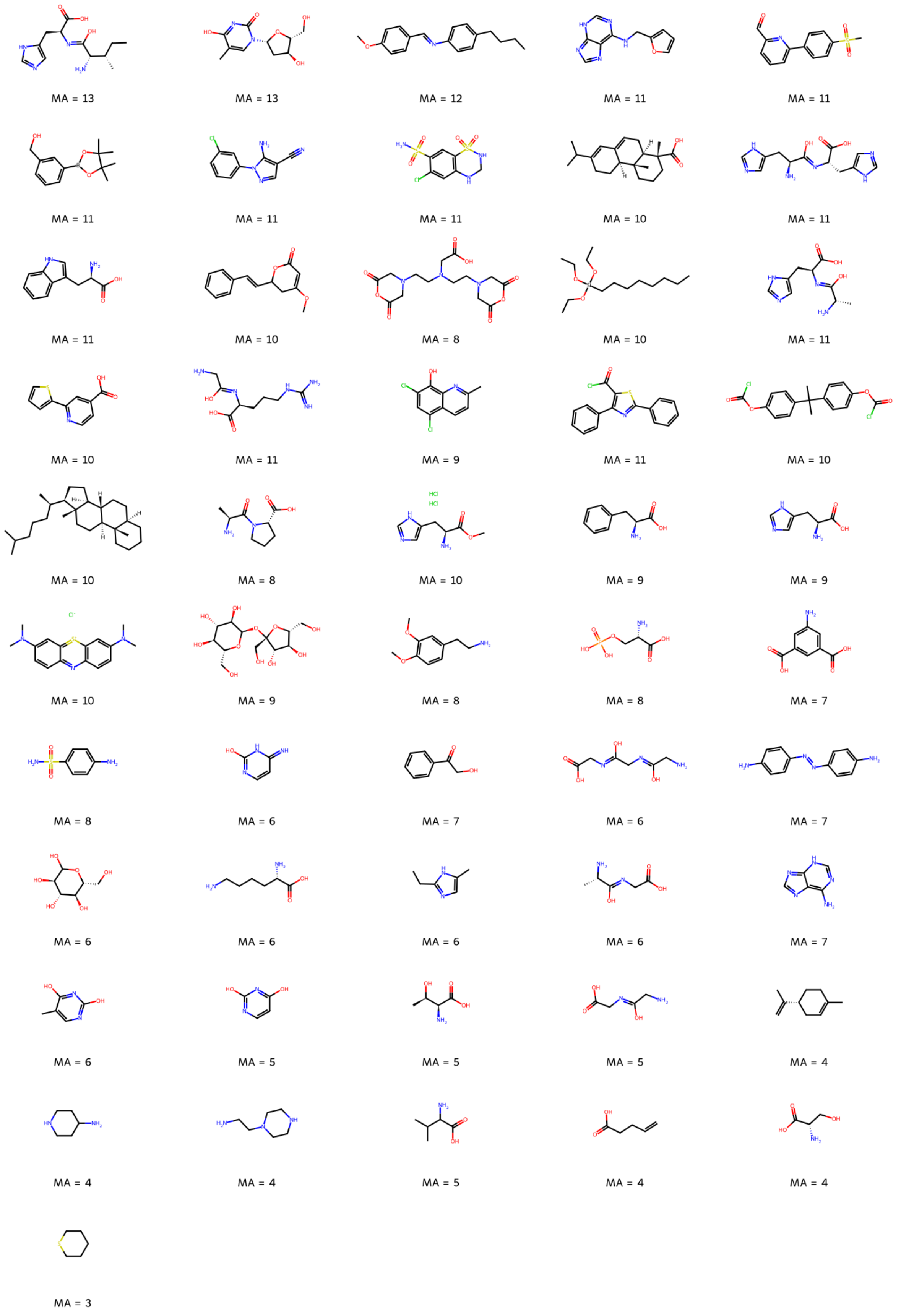

Fig. S31. Structures with calculated molecular assembly (MA) used in the experimental NMR study (part 2).

5 Combing IR and NMR data

On the set of 10,000 calculated NMR and IR spectra, we have examined our hypothesis that combined information can provide a more reliable MA prediction. We have used the models for the individual spectroscopic techniques (**Eq. 1** and **Eq. 2**) and allowed them to optimise for their relative weighting. The combined model provided a higher correlation of 0.90 using the weighted average of 0.55×NMR and 0.45×IR inferred MA (**Fig. S32**).

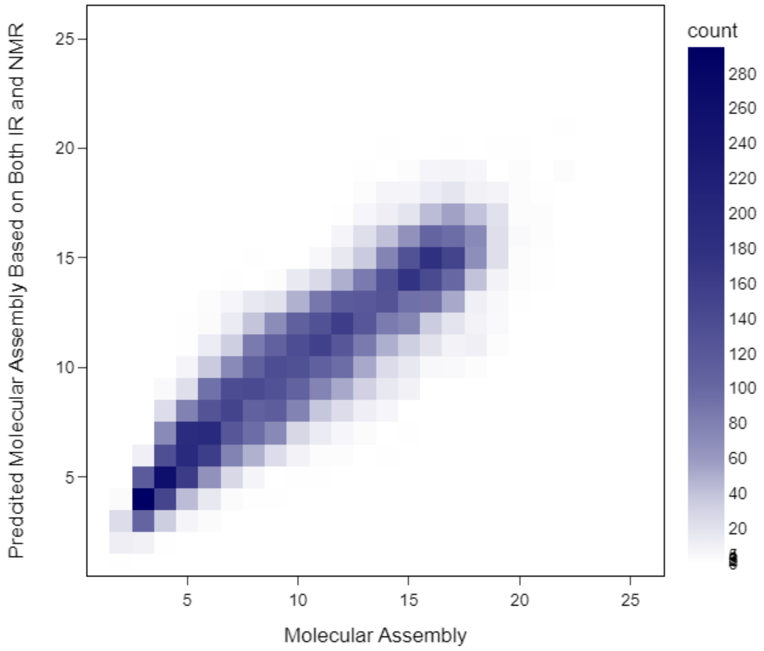

Fig. S32. Histogram of predicted MA *vs* expected MA on 10,000 compounds sample based on the fit of NMR and IR prediction using **Eq. 1** and **Eq. 2**, respectively, using a weighted average of NMR and IR of 0.55 and 0.45, respectively.

Analogously to the simulated data, the ratio for a weighted average of the models based on **Eq. 1** for NMR and the experimental model fit for IR **Eq. 4** were optimised for the experimental test sample on the available intersection of the experimental NMR and IR data, comprising 55 molecules. The weighting was 0.7 and 0.3 for the ratio of NMR and IR MA predictions, respectively, yielding a correlation of the predicted and experimental MA of 0.89 (**Fig. S33**).

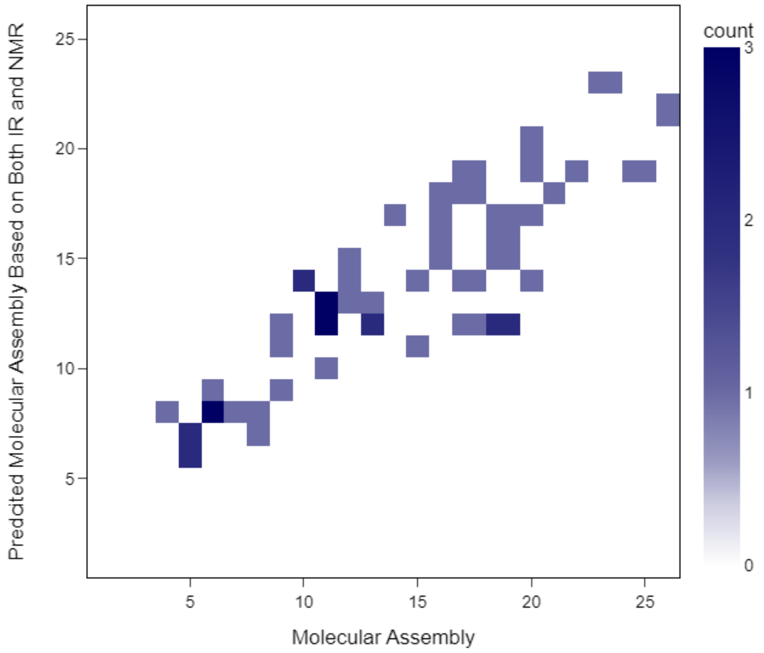

Fig. S33. Histogram of predicted MA *vs.* expected MA on 55 compounds sample based on the fit of NMR and IR prediction using **Eq. 1** and **Eq. 4**, respectively, using weighted average of NMR and IR of 0.7 and 0.3, respectively.

In **Table S1** and **S2** are summarised inferred MA from the experimental data. Note that due to experimental limitations, only for 10 compounds the MS^n data suitable for the new recursive algorithm, together with NMR and IR spectroscopies were available. In the rest, MA inference from MS was approximated by corelation of the exact mass of the compounds.

name	MA inferred from				
	MS[a)]	NMR	IR	average	MA
Ceftiofur	24.2[a)]	21.7	22.7	22.8	26
Sildenafil	21.9[a)]	19.4	19.5	20.2	25
Cefixime	20.9[a)]	19.1	17.2	19.1	22
Ketoconazole	24.5[a)]	23.3	22.2	23.4	23
Folic Acid	20.3[a)]	18.5	22.7	20.5	20
FMOC-Lys(Boc)-OH	21.6[a)]	18.3	15.9	18.6	21
N-[3,5-Bis(triflurometheyl)benzenesulfonyl]-1-methionine	20.2[a)]	11.6	14.0	15.3	19
Succinylsulfathiazole	16.3[a)]	13.2	15.9	15.1	20
Lansoprazole	16.9[a)]	17.7	16.8	17.1	19
Boc-His(Bzl)-OH	15.8[a)]	15.1	13.1	14.7	19
Sertindole	20.3[a)]	20.8	14.5	18.5	20
Fmoc-D-Asp(OtBu)-OH	18.9[a)]	16.4	18.6	18.0	20
2-(((3-Methyl-4-(2,2,2 trifluroethoxy)pyridin-2-yl)methyl)thio)-1H-benzo[d]imidazole	17.7[a)]	16.1	15.9	16.6	19
Nimodipine	26.4[a)]	19.8	17.2	21.1	16
Quinine	14.8[a)]	18.9	15.4	16.4	16
(2S)-2[(tert-Butoxycarbonyl)amino]-6-[(3-pyridinylcarbonyl)amino] hexanoic acid	16.1[a)]	15.6	15.9	15.8	16
Prochloraz	17.2[a)]	13.5	16.3	15.7	17
2'-Deoxyadenosine 5'-monophosphate	20.3	11.4	12.2	14.7	17
Thymolphthalein	19.8[a)]	19.1	14.5	17.8	17
Penicillin G	15.3 [a)]	14.7	15.9	15.3	16
Cresol Red	17.6 [a)]	19.4	13.1	16.7	16
(5-Bromo-2-hydroxy-phenyl)-(1-phenyl-1H-pyrazol-4-yl) Ketone	21.5	16.5	16.8	18.2	14
Fluconazole	14.0 [a)]	12.1	16.8	14.3	13
Bis(2-carbopentyloxy-3,5,6-trichlorophenyl) oxalate	44.3	15.0	12.7	24.0	15
Dipyridamole	32.5	9.3	14.0	18.6	13

Table S1. Inferred MA from the experimental data, average prediction and expected value. a) Values inferred from the exact mass of the compounds.

name	MA inferred from				MA
	MS[a)]	NMR	IR	average	
H-Ala-Phe-Gly-OH	13.4[a)]	12.7	13.6	13.2	12
Thioflavin T	14.6[a)]	14.8	14.5	14.6	12
D-(-)-Salicin	13.0[a)]	13.3	10.4	12.2	11
H-Ile-His-OH	12.2[a)]	12.4	11.8	12.1	13
Thymidine	11.0[a)]	10.9	15.9	12.6	13
N-(-4-methoxybenzylidene)-4-butylaniline	16.7	13.9	14.0	14.9	12
Kinetin	9.7[a)]	12.1	16.8	12.8	11
6-[4-(Methylsulfonyl)phenyl]-2-pyridine carboxaldehyde	15.7	12.5	12.7	13.6	11
3-(Hydroxymethyl)Phenylboronic acid Pinacol ester	10.6[a)]	10.2	15.4	12.1	11
5-Amino-1-(3-chlorophenyl)-1H-pyrazole-4-carbonitrile	12.1	12.7	11.3	12.1	11
Hydrochlorothiazide	17.4	9.7	12.2	13.1	11
Abietic Acid	13.8[a)]	17.7	6.3	12.6	10
D-Tryptophan	9.2[a)]	12.9	13.6	11.9	11
Diethylenetriaminepentaacetic dianhydride	16.4[a)]	7.4	10.0	11.2	8
5,7-Dichloro-8-hydroxy-2-methyl-quinoline	13.1	12.7	10.4	12.1	9
5-alpha-Cholestane	17.1[a)]	18.0	5.4	13.5	10
L-Histidine	6.9[a)]	7.8	12.2	9.0	9
Sucrose	15.7[a)]	11.8	9.5	12.3	9
O-Phospho-L-Serine	8.3[a)]	4.9	12.2	8.5	8
H-Gly-Gly-Gly-OH	8.5[a)]	8.0	10.9	9.1	6
Glucose	8.1[a)]	6.8	11.8	8.9	6
L-Lysine	6.5[a)]	6.9	11.3	8.2	6
Adenine	5.9[a)]	7.7	9.5	7.7	7
Thymine	5.5[a)]	7.2	10.0	7.6	6
Uracil	4.9[a)]	6.4	7.2	6.2	5
L-Threonine	5.2[a)]	5.3	9.1	6.5	5
GlyGly	5.8[a)]	6.1	10.4	7.4	5
(R)-(+)-Limonene	6.0[a)]	9.5	3.6	6.4	4
DL-Valine	5.1[a)]	5.6	5.9	5.5	5

Table S2. Inferred MA from the experimental data, average prediction and expected value. a) Values inferred from the exact mass of the compounds.

6 Mixture Analysis

6.1 NMR

To deconvolute the mixture *via* NMR as a proof of concept, the mixture of two compounds was examined using ^{13}C DOSY. The experimental setup of the ^{13}C DOSY was 200 ppm spectral width, 8 TD points, 1.10 second acquisition time, 8.00 second relax delay, 0.80 second diffusion time d20, 1450 µsec gradient pulse P30 using the ^{13}C DOSY-stebpgppg1s routine with 256 scans. An example of quinine and 5-aminoisopthalic acid is presented in **Fig. S34**.

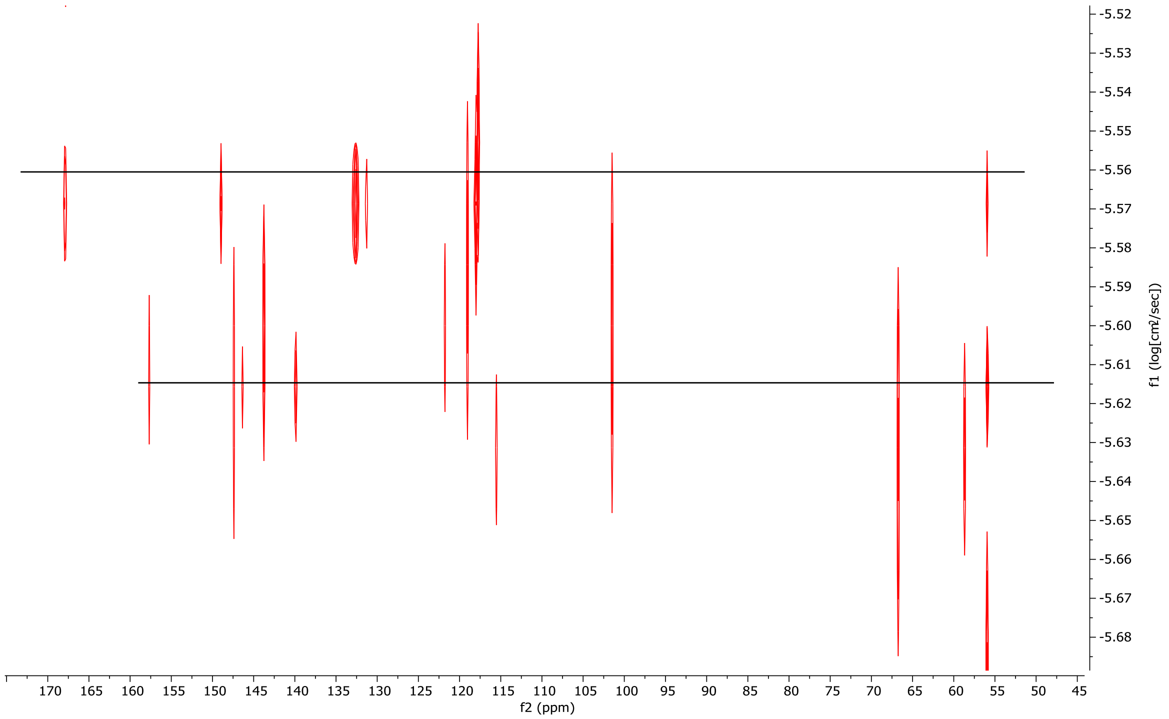

Fig. S34. ^{13}C DOSY of quinine and 5-aminoisophthalic acid mixture. Two horizontal lines guide the separation of the ^{13}C signals.

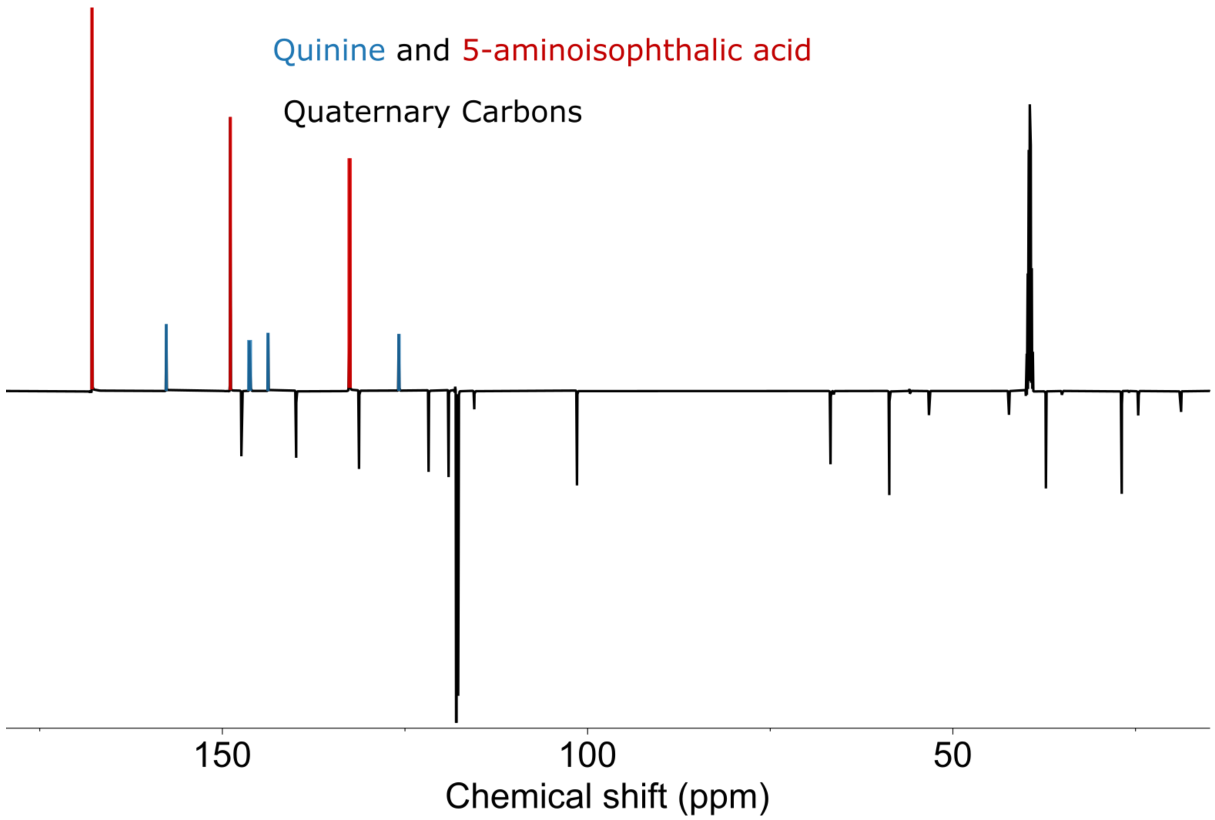

Fig. S35. DEPTQ 90 of Quinine (Cq in blue) and 5-aminoisophthalic acid (Cq in red)

7 Mass Spectrometry

7.1 Theoretical Calculations

To study the relation between MA and molecular weight (MW), a large database (PubChem) was sampled and for nearly 19 million molecules the MA was calculated using assemblyGo algorithm (as described previously) with a short timeout of 10 seconds. As the algorithm incrementally decreases MA over the duration of the calculation, and the initial values are close to the naïve MA, it could be expected that with a shorter timeout, a larger error will be observed for larger molecules. Therefore, we eventually considered only molecules with MW below 500 g·mol^{-1}. The number of molecules in that dataset was ~16.7 million. General trends of the MA and MW relation could be characterised with a linear function, as a first-order approximation, as MA = 0.0468×MW – 0.4070. The upper limit was approximated as a linear function fitted on the 99 percentile of the MA values per MW bin as MA_{max} = 0.0547×MW + 0.9458 (**Fig. S36**).

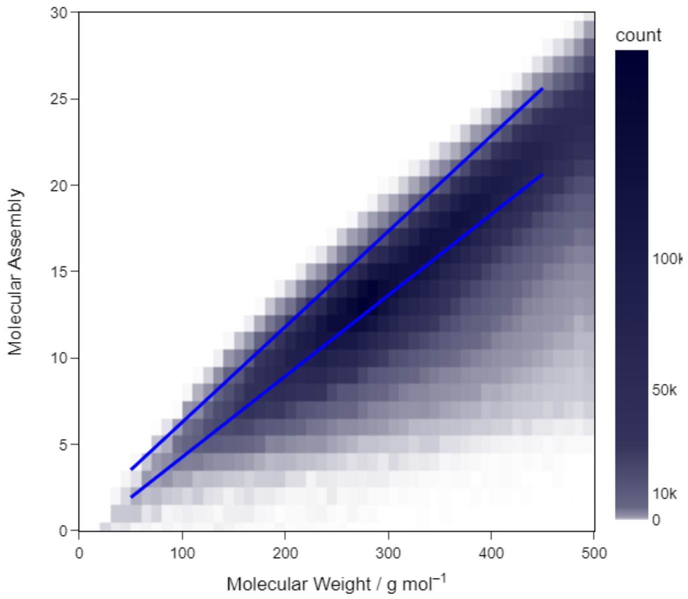

Fig. S36 Histogram of MA *vs*. MW of 16.7 million compounds sampled from PubChem database calculated using 10 s timeout. The upper limit is characterised by A_{max} = 0.0547×MW + 0.9458; the average MW-to-MA relation as MA = 0.0468×MW – 0.4070

```
                    Results: Ordinary least squares
======================================================================
Model:              OLS              Adj. R-squared:     0.789
Dependent Variable: y                AIC:                71982652.2227
Date:               2023-11-01 11:19 BIC:                71982681.4870
No. Observations:   16720274         Log-Likelihood:     -3.5991e+07
Df Model:           1                F-statistic:        6.255e+07
Df Residuals:       16720272         Prob (F-statistic): 0.00
R-squared:          0.789            Scale:              4.3372
-------------------------------------------------------------------------
          Coef.     Std.Err.        t        P>|t|      [0.025     0.975]
-------------------------------------------------------------------------
x1        0.0468      0.0000    7908.7996    0.0000     0.0468     0.0468
const    -0.4070      0.0020    -200.3950    0.0000    -0.4110    -0.4031
----------------------------------------------------------------------
Omnibus:             2930137.758     Durbin-Watson:        1.870
Prob(Omnibus):       0.000           Jarque-Bera (JB):     7013689.075
Skew:                -0.994          Prob(JB):             0.000
Kurtosis:            5.473           Condition No.:        1368
======================================================================
```

Fig. S37. Print output from the fit of MA = x_1×MW + *const.* on the whole dataset; using *statsmodels.api.OLS* in Python.(8)

```
                 Results: Ordinary least squares
=================================================================
Model:              OLS              Adj. R-squared:     0.994
Dependent Variable: y                AIC:                95.8801
Date:               2023-11-01 11:19 BIC:                99.6637
No. Observations:   49               Log-Likelihood:     -45.940
Df Model:           1                F-statistic:        7370.
Df Residuals:       47               Prob (F-statistic): 2.57e-53
R-squared:          0.994            Scale:              0.39808
-------------------------------------------------------------------
         Coef.     Std.Err.       t       P>|t|     [0.025    0.975]
-------------------------------------------------------------------
x1       0.0547      0.0006    85.8476    0.0000    0.0534    0.0560
const    0.9458      0.1858     5.0893    0.0000    0.5719    1.3197
-----------------------------------------------------------------
Omnibus:              20.644       Durbin-Watson:         0.734
Prob(Omnibus):        0.000        Jarque-Bera (JB):      33.993
Skew:                 -1.290       Prob(JB):              0.000
Kurtosis:             6.162        Condition No.:         601
=================================================================
```

Fig. S38 Print output from the fit of MA = x_1×MW + *const.* on the 99 percentile per MW bin; using *statsmodels.api.OLS* in Python.(8)

To characterise the typical MA distribution of specific MW, skew-normal distribution provided a good fit. The fit was performed for every MW bin and the extracted parameters: skewness *a*, location *loc* and *scale* were used to fit the general trend. Simplified prediction of the *a*, *loc* and *scale* parameters for specific MW were fitted as: *a* = –0.0083×MW + 0.1117 (**Fig. S39**); *loc* = 0.0539×MW – 0.4061 (**Fig. S40**); *scale* = 0.0074×MW + 0.5108 (**Fig. S41**).

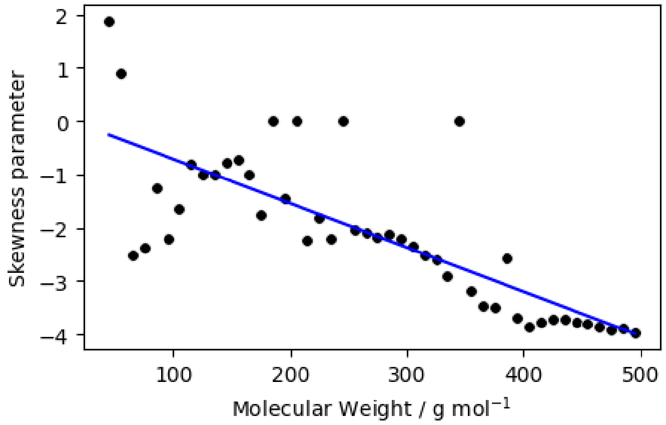

Fig. S39 General trend of the skewness parameter *vs*. MW, fitted as: *a* = –0.0083×MW + 0.111

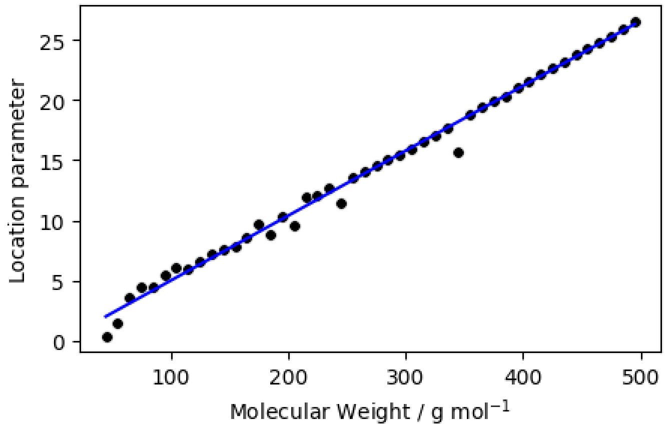

Fig. S40 General trend of the location parameter *vs*. MW, fitted as: *loc* = 0.0539×MW – 0.4061.

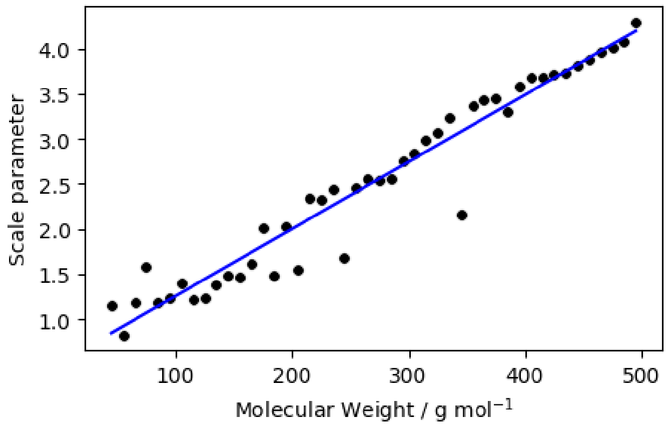

Fig. S41 General trend of the scale parameter *vs*. MW, fitted as *scale* = 0.0074×MW + 0.5108.

7.2 Recursive MA algorithm

Examining the distribution of MA versus molecular weight (MW) reveals that the uncertainty in MA is reduced at low MW. When multi-level fragmentation data is available *via* tandem mass spectrometry, a recursive logic can take advantage of the tighter uncertainty bounds at low molecular weight to yield an improved MA estimate. Our recursive MA algorithm, represented as the mathematical function *MA* operating on a molecule or fragment *f*, hinges on the following operating principles, summarized in **Fig. S42**.

1. Tabulated monoisotopic masses for period table elements. Fragments exactly matching an isotope trigger a base case: $MA(f) = 0$.
2. A second base case is used when *f* has no child ions and cannot be identified as an element. An upper bound that can be calculated solely based on the molecular weight of a molecule or fragment (discussed below).
3. The recursive case is used when at least one child ion is present. For each child ion *c*, $MA(c)$ along with the MA of its associated complement mass $MA(c') = MA(f - c)$ is calculated. Note that the complement ion may not be observed.
4. If requested by the user, the algorithm can extend the search for child ions to the current MS level by examining pairs of ions i_1 and i_2 among *M*'s siblings that are complementary with respect to *M, i.e.* $i_1 + i_2 = M$. This functionality is essential when higher level MS data is not available.

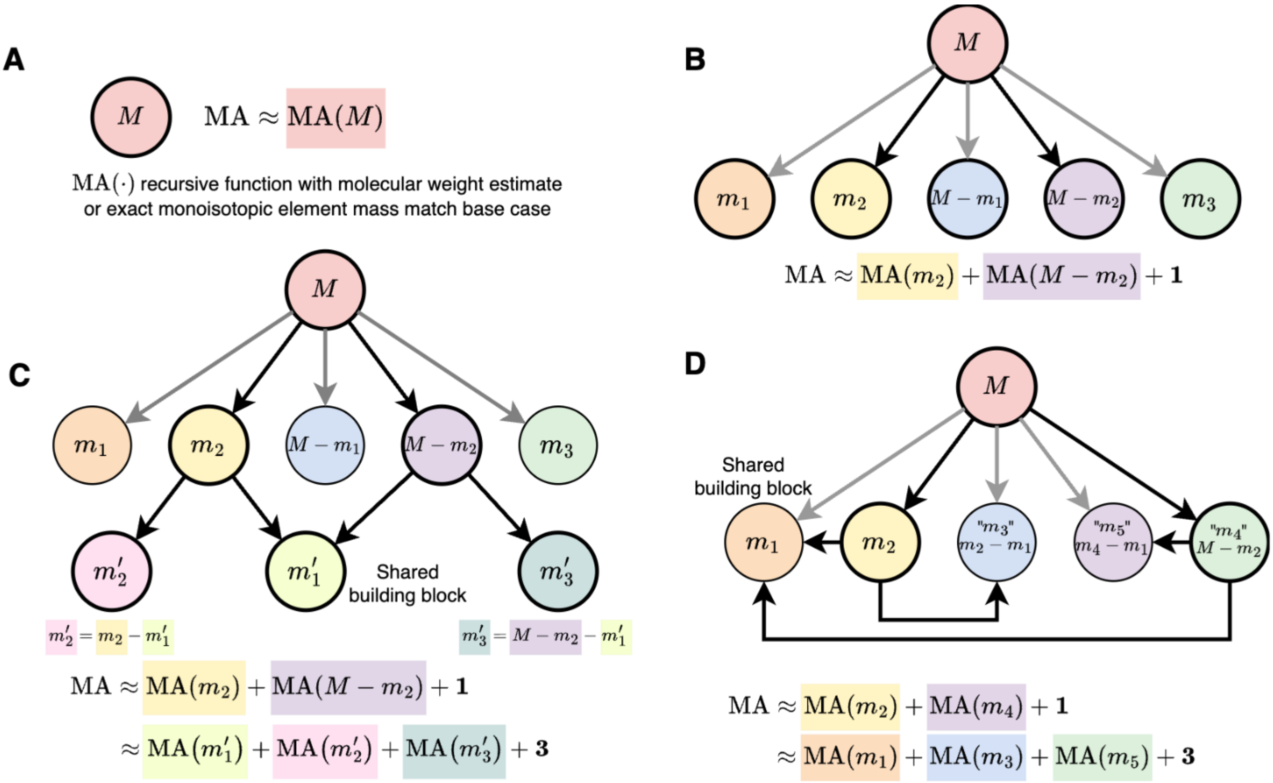

Fig. S42 . Scenarios encountered during execution of the recursive MA algorithm. (A) Base case: for a molecule or fragment with no associated child ions, MA is estimated using molecular weight, either via an exact match to the monoisotopic mass of an element (MA = 0) or the statistical MA ~ MW relationship. (B) When child ions are present, the algorithm recursively evaluates the possibility of forming *M* from every pair of child ions .

The molecular weight estimate base case is informed by the empirical relationship between MW and maximum (worst-case) MA, depicted in **Fig. S43**, where each point represents the 99th MA percentile within a given mass bucket. Bucket boundaries follow logarithmic spacing to give equal priority to different mass scales. This empirical distribution of points can be fit to a piecewise linear function of the following approximate form.

$$\mathrm{MA(MW)} = \begin{cases} 0, & x < 19 \\ \dfrac{(\mathrm{MW} - 19)}{13.5}, & x \geq 0 \end{cases}$$

It can be shown that this empirical fit closely follows a form mandated by assembly theory. This derivation is based on two fundamental assumptions.

1. The MA of fundamental building blocks is 0.
2. In the absence of structural reuse (worst-case or maximum MA), fragmenting a molecule into roughly equal parts gives the following relationship (constant 1 representing one composition step).

$$\text{MA}(\text{MW}) = \text{MA}\left(\frac{\text{MW}}{2}\right) + \text{MA}\left(\frac{\text{MW}}{2}\right) + 1$$
$$= 2\text{MA}\left(\frac{\text{MW}}{2}\right) + 1$$

Recursive application of this rule *N* times gives

$$\text{MA}(\text{MW}) = 2^{\text{MA}\left(\frac{\text{MW}}{2^N}\right)} + \sum_{n=0}^{N-1} 2^n$$
$$= 2^N \text{MA}\left(\frac{\text{MW}}{2^N}\right) + 2^N - 1.$$

When subdivision reaches the level of any building block with molecular weight MW_0, i.e. $\frac{\text{MW}}{2^N} = \text{MW}_0$, given $\text{MA}(\text{MW}_0) = 0$ we obtain

$$\text{MA}(\text{MW}) = 2^N \text{MA}(\text{MW}_0) + 2^N - 1$$
$$= 2^N - 1$$
$$= \frac{\text{MW} - \text{MW}_0}{\text{MW}_0}.$$

In light of this derivation, the empirical formula seems to indicate that typical values of MW_0 lie between 13 and 19, in line with the composition of typical organic compounds from the elements boron to fluorine.

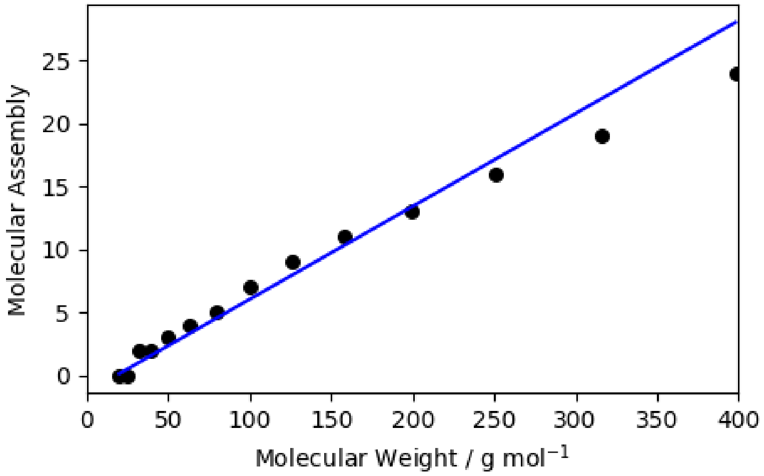

Fig. S43 Fit of the piecewise linear function of molecular weight to logarithmically-binned 99 percentile MA values, as an upper limit to MA(MW).

7.3 Experimental Mass Spectrometry

For the experimental multi-level fragmentation mass spectrometry, stock solutions were prepared using 10 mg of the sample per 10 mL of acetonitrile and further diluted using 250 µL of stock solution and 9.75 mL of acetonitrile yielding a final sample concentration of approx. 8×10^{-5} M. These samples were filtered through a 0.45 µm nylon syringe filter prior to MS analysis. Samples were analysed for 6 minutes by direct injection using a Thermo Scientific Orbitrap Fusion Lumos Tribrid mass spectrometer with a HESI ion source applying a +5.0 kV voltage. The ion transfer tube temperature was set to 290 °C and the vaporiser temperature was set to 250 °C. Samples were analysed *via* two different Single Ion Monitoring (SIM) methods investigating firstly a stepped MS^3 procedure and secondly an MS^5 procedure. The stepped MS^3 procedure involved fragmenting the target MS^1 analyte *via* HCD at 6 discrete energy levels (15, 25, 35, 45, 55, 65%, normalised values as implemented in the machine) before collating them into 2 MS^2 spectra (15, 25, 35%) and (45, 55, 65%). The MS^2 spectra were each analysed via a data-dependent acquisition (DDA) method where the 40 most abundant ions from each were selected for MS^3 fragmentation. Dynamic exclusion of ions was set for 120 seconds after the ion had been selected six times in 60 seconds. The resolution of the SIM scan was 120000 and the MS^2 and MS^3 fragmentation spectra were set at 30000 with an isolation window of 5 ppm. The MS^5 procedure achieved ion fragmentation *via* HCD set to 35% with an isolation window of 10 ppm. The spectra were analysed via a DDA method where the 40 most abundant MS^2 ions were selected for MS^3 fragmentation, the 30 most abundant MS^3 ions were selected for MS^4 fragmentation, and the 20 most abundant MS^4 ions were selected for MS^5 fragmentation. The dynamic exclusion and resolution remained the same as the stepped MS^3 method with the MS^4 and MS^5 also set at 30000.

All molecules with the MW 300 ± 5 $g\cdot mol^{-1}$ used in the multi-level fragmentation mass spectrometry are depicted in **Fig. S44**.

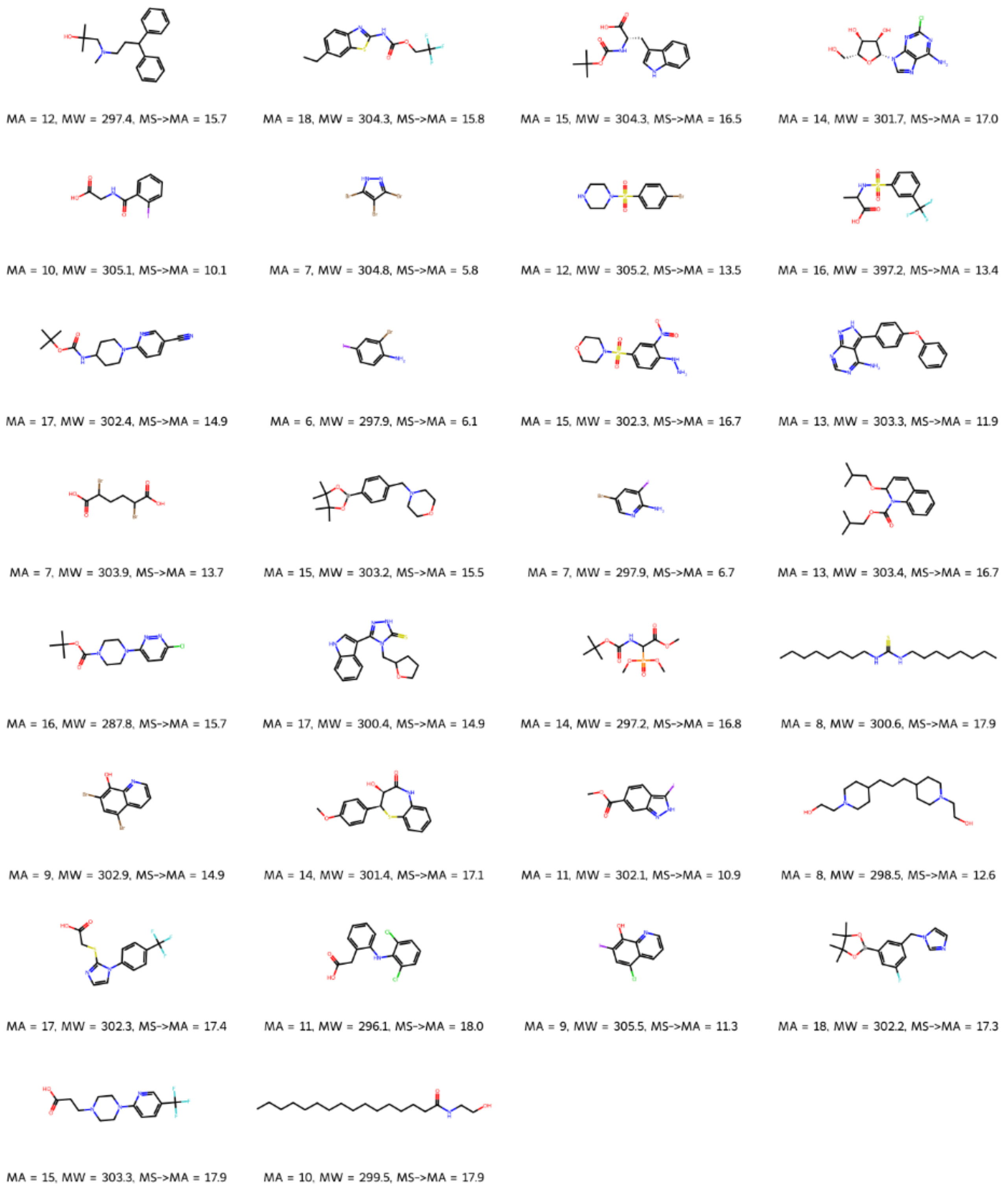

Fig. S44 30 Molecules with up to MS5 data with a similar mass of 300 ±5 g·mol^{-1} and various MA from 6 to 18.

8 References

1. S. M. Marshall, *et al.*, Identifying molecules as biosignatures with assembly theory and mass spectrometry. *Nat Commun* **12**, 3033 (2021).

2. G. Rücker, C. Rücker, Automatic Enumeration of All Connected Subgraphs. *MATCH Comm. in Math. in Comp. Chem.*, 145–149.

3. B. D. McKay, A. Piperno, Practical graph isomorphism, II. *Journal of Symbolic Computation* **60**, 94–112 (2014).

4. Y. Liu, *et al.*, Exploring and mapping chemical space with molecular assembly trees. *Sci. Adv.* **7**, eabj2465 (2021).

5. S. M. Marshall, D. G. Moore, A. R. G. Murray, S. I. Walker, L. Cronin, Formalising the Pathways to Life Using Assembly Spaces. *Entropy* **24**, 884 (2022).

6. S. Kuhn, N. E. Schlörer, Facilitating quality control for spectra assignments of small organic molecules: nmrshiftdb2 - a free in-house NMR database with integrated LIMS for academic service laboratories: Lab administration, spectra assignment aid and local database. *Magn. Reson. Chem.* **53**, 582–589 (2015).

7. G. Landrum, *et al.*, rdkit/rdkit: 2022_09_4 (Q3 2022) Release (2023) https:/doi.org/10.5281/ZENODO.591637 (February 8, 2023).

8. S. Seabold, J. Perktold, Statsmodels: Econometric and Statistical Modeling with Python in (2010), pp. 92–96.

9. C. Bannwarth, *et al.*, Extended tight-binding quantum chemistry methods. *WIREs Comput Mol Sci* **11** (2021).

10. K. M. Jablonka, L. Patiny, B. Smit, Making Molecules Vibrate: Interactive Web Environment for the Teaching of Infrared Spectroscopy. *J. Chem. Educ.* **99**, 561–569 (2022).

11. , Python API for the extended tight binding program.

12. S. K. Padamati, *et al.*, Transient Formation and Reactivity of a High-Valent Nickel(IV) Oxido Complex. *J. Am. Chem. Soc.* **139**, 8718–8724 (2017).

13. G. te Velde, *et al.*, Chemistry with ADF. *J. Comput. Chem.* **22**, 931–967 (2001).

14. M. Swart, F. M. Bickelhaupt, QUILD: QUantum-regions interconnected by local descriptions. *J. Comput. Chem.* **29**, 724–734 (2008).

15. A. D. Becke, Density-functional exchange-energy approximation with correct asymptotic behavior. *Phys. Rev. A* **38**, 3098–3100 (1988).

16. J. P. Perdew, Density-functional approximation for the correlation energy of the inhomogeneous electron gas. *Phys. Rev. B* **33**, 8822–8824 (1986).

17. S. Grimme, J. Antony, S. Ehrlich, H. Krieg, A consistent and accurate *ab initio* parametrization of density functional dispersion correction (DFT-D) for the 94 elements H-Pu. *The Journal of Chemical Physics* **132**, 154104 (2010).

18. A. Klamt, G. Schüürmann, COSMO: a new approach to dielectric screening in solvents with explicit expressions for the screening energy and its gradient. *J. Chem. Soc., Perkin Trans. 2*, 799–805 (1993).

19. R. Burger, P. Bigler, DEPTQ: Distorsionless Enhancement by Polarization Transfer Including the Detection of Quaternary Nuclei. *Journal of Magnetic Resonance* **135**, 529–534 (1998).

20. P. Bigler, R. Kümmerle, W. Bermel, Multiplicity editing including quaternary carbons: improved performance for the13C-DEPTQ pulse sequence. *Magn. Reson. Chem.* **45**, 469–472 (2007).
