# Supplementary material for: Investigating and Quantifying Molecular Complexity Using Assembly Theory and Spectroscopy
Source: ACS Cent Sci. 2024 Apr 18;10(5):1054–64. doi: 10.1021/acscentsci.4c00120 (PMC11117308; doi:10.1021/acscentsci.4c00120)
Supplement: Supplementary file 2 — oc4c00120_si_002.pdf [file oc4c00120_si_002.pdf]

oc-2024-001208.R1

Name: Peer Review Information for "Investigating & Quantifying Molecular Complexity using Assembly Theory and Spectroscopy"

First Round of Reviewer Comments

Reviewer: 1

Comments to the Author

This contribution to ACS Central Science from the Cronin group is Glasgow continues a theme of measuring and using molecular complexity as an agnostic biosignature. The Cronin group previously put forth a method of using tandem-MS to measure pathway assembly, now referred to a molecular assembly (MA). MA relies on mapping a chemical structure onto the least number of bonds patterns that could be used, in a repetitive fashion, to construct a molecule. MA is entirely a Cronin group measurement and is thus a novel vantage point from which to determine molecular complexity, to complement a variety of other historical approaches. Previous, the group had devised a tandem-MS method to measure MA, but in the paper improves the experimental approach by combining tanden-MS, with the fingerprint region of an IR, along with carbon count in 13C-NMR spectroscopy. This reviewer read the paper with great interest and believes it should be accepted with some revision for improvement to a general audience, and then a discussion of possible caveats.

1) All the figures in the paper are clear and make sense except for Figure 2. Part a discusses “queue”, “pathways”, and “jobs”, and these terms are not clear as to their meaning from the text. Further, parts b and c really are not clear. “Remnant”, “duplicates … 1, 2, 3”, etc. What? Then “naïve MA”, “approximate MA”, “exact MA”, and “split branch”. What? These terms are primarily introduced in the figure caption, and not discussed in the bulk of the text. This reviewer really could not figure out what this all meant.

2) Much of what is presented here as an experimental approach is meant to complement the recent paper in Nature from this group. Both here, and in the Nature paper, copy number is of critical importance. Totally agree!

But this begs the question of how the method could be used in practice on a large mixture of random complex molecules that might arise from an abiotic source. In this paper, one mixture is analyzed (and frankly, it is a bit of a red herring in this paper and could be saved for a later paper where mixtures are the focus). In order words, this reviewer suspects that when there is enough quantity of material to get an NMR, IR, or tandem MS that is composed of random mixtures of junk (e.g. a few mgs of totally random abiotic polymeric goo), coincidental overlap of peaks in NMR, IR, and MS would give a reasonably large experimental MA. This may likely be encountered in a real

NASA mission. In other words, plenty of junk to get signals, but it is all just "junk". A discussion of this concern is beyond the scope of this paper, so this reviewer is simply posing this question to the Cronin group.

Summary, this paper can be accepted if the explanation of Figure 2 is improved. It is a worthy contribution of the continuing molecular assembly theories from the Cronin group for biosignatures beyond earth.

Reviewer: 2

Comments to the Author

The manuscript presents approaches to experimentally estimate the molecular complexity of organic compounds using common laboratory spectroscopic techniques like nuclear magnetic resonance (NMR), infrared (IR) spectroscopy, and tandem mass spectrometry (MS/MS). It is important to acknowledge the extreme novelty in this – this is the first effort to my knowledge to ground a formal measure of complexity in molecular systems with its empirical measurement, and indeed to corroborate that grounding across a multimodal suite of techniques.

Molecular complexity is quantified using the Assembly Index, which is based on the shortest number of recursively constructed steps to build a molecule from simple building blocks. This length of this shortest pathway defines the Molecular Assembly index (MA), which is the proposed mathematical measure of complexity that forms the foundation of this work.

Computational modelling on 10,000 molecules shows the number of peaks in IR spectra and types of carbon atoms in NMR spectra both correlate well with the MA. This was shown to be validated for both experimental techniques using samples of 99 (IR) and 101 (NMR) compounds. A recursive algorithm using MS/MS fragmentation data to construct a hierarchy of molecular fragments was also shown to estimate MA with good accuracy, with experimental results confirmed for 101 compounds. A multimodal suite of techniques was then shown to improve MA prediction as compared to individual techniques and also that MA can be measured for mixtures using NMR to distinguish components. Overall, the work is very detailed and thorough and clearly demonstrates spectroscopic techniques can reliably estimate molecular complexity in terms of the MA.

What is exciting about this approach is can be implemented without needing complete structural elucidation. This enables using MA as a metric to explore complex chemical spaces and identify signatures of evolutionary processes even in systems where we do not know the exact chemical composition, e.g. I can see a lot of utility for this in identifying the complexity of uncharacterized

metabolites (e.g., as in recent studies of underground metabolism), in studying geochemical environments to fingerprint life, on unculturable organisms etc etc .. the list goes on.

The reason I find this particularly exciting is that as a complexity theorist I know all too well that most of the measures we use to characterize complexity are not empirically validated, and indeed are often not even empirically validatable. Having an approach like MA, that is rigorously tied to experimental measurements we can do allows the possibility of bridging chemical complexity studies to the kinds of frontiers I note above.

I therefore am enthusiastic for this manuscript to be published in ACS Central Science as I am sure it will be of broad interest to your readership. Prior to publication I do have a few minor suggestions that might help improve the overall readability of the manuscript. I think this could become a very important reference point for the field so the suggestions I make in that light are to make the manuscript as clear to a broad readership as possible. They are based on my own reading of the manuscript and what might have made it easier for me to understand the work clearly more quickly, but they are not strictly necessary as the paper is overall already well written and I could readily follow it.

Figure 1 – the two panels (A) and (B) show an example molecular assembly graph and the corresponding spectra with NMR, IR and mass spec, respectively. This figure might be more illuminating for the reader if an additional panel were included that show how the two map, e.g., including another panel with a mock up to set the idea of linear fits and linear regression based on carbon types that show the transformation from spectra to MA value.

Figure 2 Panel B is nice, but the depiction of the duplicate and remnant fragments is better depicted in the supplement (Figure S1) where the duplicate and remnant are colored differently, and the steps are clearly elucidated. I like the structure of the table in B but I might pull more of how the ideas is presented from S1 because it is so clear there how it works.

p. 9 line 40 remove "the" in front of "assembly theory"

Different compound sets are used for each of the three techniques with slightly different MA ranges. Obviously these are chosen as experimental reference sets for the different experimental techniques for a reason, but it would be good to write a sentence in each experimental section motivating the choice of compound set. For example the 101 compounds used for C13 NMR have a range of MA of 3-26, and I assume these where chosen to cover a diversity of compounds with

carbons having different numbers of constraints but this should be made clear (particularly so if I am not right in my guess about why this set is chosen). Same for IR and mass spec datasets.

p. 19 line 9 – "exploring the extent of" is missing what it is referring to

Author's Response to Peer Review Comments:

From: Professor Leroy (Lee) Cronin BSc. (Hons) DPhil. FRSC CCHEM FRSE
Regius Professor of Chemistry, University of Glasgow, Advanced Research Centre, Level 5, Digital Chemistry, 11 Chapel Lane, Glasgow, G11 6EW
Email: lee.cronin@glasgow.ac.uk; http//www.croninlab.com

Deputy Editor,
ACS Central Science

16th March 2024

Dear Editor,

Thank you for your email regarding our manuscript oc-2024-001208, ***'Investigating & Quantifying Molecular Complexity using Assembly Theory and Spectroscopy'*** by Michael Jirasek, Abhishek Sharma, Jessica R. Bame, Nicola Bell, Stuart M. Marshall, Cole Mathis, Alasdair Macleod, Geoffrey J. T. Cooper, Marcel Swart, Rosa Mollfulleda, Leroy Cronin to be considered for publication in *ACS Central Science.*

We are excited by the referee reviews have been able to address all their comments, and the editorial comments in this revision. The point by point replies are as follows:

Q: *ABSTRACT: Please make sure the word count does not exceed 200 words*.

A: Abstract has been shortened to bellow 200 words.

Q: *REF 42: Please format properly, and provide the full information*

A: We have changed reference 42 from the web to a relevant paper as the website is not working anymore.

Q: *GENERAL REF FORMATTING: Periodical references should contain authors' surnames followed by initials, article title, journal abbreviation, year, volume number, and page range. Refs with more than 10 authors should list the first 10 and then be followed by "et al." Web sources must include access date.*

A: We have formatted the references to the required style, the web link was replaced and we have added access date to a reference to the github repository.

Q: *TOC MISSING: Provide a TOC image per journal guidelines (3.25 in. × 1.75 in. (8.25 cm × 4.45 cm) ; on the last page of the Manuscript) with the heading "TOC Graphic" above the graphic. Make sure to designate the file as "Graphic for Manuscript."*

A: TOC graphic was added to the required place.

Q: *SYNOPSIS MISSING: The synopsis should be no more than 200 characters (including spaces) and should reasonably correlate with the TOC graphic. The synopsis is intended to explain the importance of the article to a broader readership across the sciences. Please place your synopsis in the manuscript file after the TOC graphic, and label it as "Synopsis."*

A: We have added synopsis to the required place.

Q: *SI PG#S: The supporting information pages must be numbered consecutively, starting with page S1.*

A: Page numbering in the supporting information has been updated to include the 'S'.

Reviewer: 1

Q: *All the figures in the paper are clear and make sense except for Figure 2. Part a discusses "queue", "pathways", and "jobs", and these terms are not clear as to their meaning from the text. Further, parts b and c really are not clear. "Remnant", "duplicates … 1, 2, 3", etc. What? Then "naïve MA", "approximate MA", "exact MA", and "split branch". What? These terms are primarily introduced in the figure caption, and not discussed in the bulk of the text. This reviewer really could not figure out what this all meant.*

A: We have included more discussion about the AssemblyGo algorithm and differences from the previous approach used. We also moved some text discussing details of the algorithm in front of the Figure 2, to make it more clear for the reader.

Reviewer: 2

Q: *Figure 1 – the two panels (A) and (B) show an example molecular assembly graph and the corresponding spectra with NMR, IR and mass spec, respectively. This figure might be more illuminating for the reader if an additional panel were included that show how the two map, e.g.,*

including another panel with a mock up to set the idea of linear fits and linear regression based on carbon types that show the transformation from spectra to MA value.

A: Authors thank the reviewer for the suggestion. However, we believe the main purpose of **Figure 1** is to illustrate the concept of molecular assembly space and to demonstrate spectroscopic features of a single molecule. The linear regression fit comes after the analysis over the large sample of molecules, on which the principle of inference is shown in detail in the figures later in the manuscript. We have changed the figure legend to point the reader to figures 3-6.

Additionally, we have provided a TOC, which shows the conceptual workflow inferring MA from unknown samples using spectroscopies and regression analysis on large dataset.

Q: Figure 2 Panel B is nice, but the depiction of the duplicate and remnant fragments is better depicted in the supplement (Figure S1) where the duplicate and remnant are colored differently, and the steps are clearly elucidated. I like the structure of the table in B but I might pull more of how the ideas is presented from S1 because it is so clear there how it works.

A: We have made an edit to the ms to point the reader to the S1 figure if they are interested.

Q: *p. 9 line 40 remove "the" in front of "assembly theory"*

A: We have removed the word as recommended.

Q: *Different compound set s are used for each of the three techniques with slightly different MA ranges. Obviously these are chosen as experimental reference sets for the different experimental techniques for a reason, but it would be good to write a sentence in each experimental section motivating the choice of compound set. For example the 101 compounds used for C13 NMR have a range of MA of 3-26, and I assume these where chosen to cover a diversity of compounds with carbons having different numbers of constraints but this should be made clear (particularly so if I am not right in my guess about why this set is chosen). Same for IR and mass spec datasets.*

A: We have added short comments for reasoning what molecules were chosen for NMR, IR and MS.

Q: *p. 19 line 9 – "exploring the extent of" is missing what it is referring to*

A: We have updated the text in the respective part as: "extent of life".

Thanks again for considering our manuscript and hope to hear from you soon.

Best wishes,

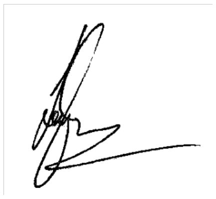

Lee Cronin
